# Supplementary material for: Finding Children with High Risk of Non-Vaccination in 92 Low- and Middle-Income Countries: A Decision Tree Approach
Source: Vaccines (Basel). 2021 Jun 13;9(6):646. doi: 10.3390/vaccines9060646 (PMC8231774; doi:10.3390/vaccines9060646)
Supplement: Supplementary file 1 [file vaccines-09-00646-s001.zip › vaccines-1241213-supplementary.pdf]

Supplementary materials

Section A

In order to take into consideration the number of children aged 12-23 months living in each country, individual sample weights were adjusted using the following equation:

$$w_{ij,adj} = \left(\frac{w_{ij}}{\sum_i w_{ij}}\right)\left(\frac{pop_j}{\sum_j pop_j}\right)N$$

where:
i indicates a child and j a country
$w_{ij,adj}$ is the adjusted individual sample weight
w_{ij} is the original sample weight
pop_j is the population of children aged 12-23 months in the country j
N is the total number of children in the sample

Section B

Table S1. – National zero-dose prevalence and confidence interval.

Country	Year	Source	Zero dose prevalence	CI 95%
Afghanistan	2015	DHS	13.1%	(11.4 - 15.0%)
Algeria	2012	MICS	1.6%	(1.0 - 2.4%)
Angola	2015	DHS	19.8%	(17.7 - 22.1%)
Armenia	2015	DHS	0.6%	(0.2 - 1.7%)
Bangladesh	2014	DHS	2.0%	(1.4 - 2.9%)
Belize	2015	MICS	1.6%	(0.5 - 4.9%)
Benin	2017	DHS	11.0%	(9.3 - 12.8%)
Bosnia and Herzegovina	2011	MICS	0.3%	(0.1 - 1.3%)
Burkina Faso	2010	DHS	1.8%	(1.2 - 2.7%)
Burundi	2016	DHS	0.3%	(0.2 - 0.7%)
Cambodia	2014	DHS	2.4%	(1.6 - 3.6%)
Cameroon	2014	MICS	3.9%	(2.8 - 5.3%)
CAR	2010	MICS	9.3%	(7.8 - 11.1%)
Chad	2014	DHS	18.5%	(16.5 - 20.7%)
Colombia	2010	DHS	1.7%	(1.2 - 2.4%)
Comoros	2012	DHS	10.7%	(8.2 - 13.8%)
Congo Brazzaville	2014	MICS	6.3%	(5.0 - 7.9%)
Congo Democratic Republic	2017	MICS	20.5%	(17.9 - 23.3%)
Costa Rica	2011	MICS	0.1%	(0.0 - 0.8%)
Côte d'Ivoire	2016	MICS	11.9%	(9.9 - 14.2%)
Cuba	2014	MICS	0.3%	(0.1 - 0.7%)
Dominican Republic	2014	MICS	4.0%	(3.2 - 5.1%)
Egypt	2014	DHS	0.0%	(0.0 - 0.2%)
El Salvador	2014	MICS	0.4%	(0.1 - 1.9%)
Eswatini	2014	MICS	1.6%	(0.8 - 3.4%)
Ethiopia	2016	DHS	16.2%	(13.5 - 19.3%)
Gabon	2012	DHS	4.8%	(3.3 - 6.9%)
Gambia	2018	MICS	1.5%	(1.0 - 2.3%)
Ghana	2017	MICS	3.2%	(2.1 - 5.0%)

Country	Year	Source	Zero dose prevalence	CI 95%
Guatemala	2014	DHS	0.6%	(0.3 - 1.1%)
Guinea	2018	DHS	23.0%	(19.8 - 26.4%)
Guinea Bissau	2014	MICS	3.3%	(2.4 - 4.5%)
Guyana	2014	MICS	2.8%	(1.5 - 5.3%)
Haiti	2016	DHS	9.8%	(7.5 - 12.5%)
Honduras	2011	DHS	0.4%	(0.2 - 1.0%)
India	2015	DHS	6.1%	(5.7 - 6.4%)
Indonesia	2017	DHS	7.0%	(5.9 - 8.1%)
Iraq	2018	MICS	3.0%	(2.4 - 3.9%)
Jamaica	2011	MICS	0.5%	(0.1 - 2.0%)
Jordan	2017	DHS	6.9%	(5.2 - 9.1%)
Kazakhstan	2015	MICS	1.6%	(1.0 - 2.6%)
Kenya	2014	DHS	1.7%	(1.2 - 2.5%)
Kiribati	2018	MICS	38.2%	(33.4 - 43.4%)
Kosovo	2013	MICS	1.0%	(0.3 - 3.0%)
Kyrgyzstan	2018	MICS	2.4%	(1.2 - 4.6%)
Laos	2017	MICS	13.0%	(11.3 - 14.9%)
Lesotho	2018	MICS	2.5%	(1.3 - 4.6%)
Liberia	2013	DHS	1.7%	(1.0 - 2.7%)
Madagascar	2018	MICS	18.7%	(16.6 - 21.0%)
Malawi	2015	DHS	1.6%	(1.1 - 2.3%)
Maldives	2016	DHS	8.1%	(5.7 - 11.5%)
Mali	2018	DHS	14.5%	(11.9 - 17.4%)
Mauritania	2015	MICS	9.7%	(7.8 - 11.9%)
Mexico	2015	MICS	5.6%	(3.7 - 8.3%)
Moldova	2012	MICS	0.0%	-
Mongolia	2018	MICS	1.3%	(0.7 - 2.4%)
Montenegro	2013	MICS	1.4%	(0.4 - 5.5%)
Mozambique	2015	DHS	5.1%	(3.2 - 8.0%)
Myanmar	2015	DHS	7.9%	(5.4 - 11.3%)
Namibia	2013	DHS	4.4%	(2.9 - 6.7%)
Nepal	2016	DHS	0.8%	(0.4 - 1.8%)
Niger	2012	DHS	4.4%	(3.4 - 5.6%)
Nigeria	2018	DHS	19.5%	(17.9 - 21.3%)
North Macedonia	2011	MICS	2.1%	(0.8 - 5.4%)
Pakistan	2017	DHS	3.7%	(2.6 - 5.3%)
Panama	2013	MICS	1.6%	(0.8 - 3.1%)
Papua New Guinea	2016	DHS	24.2%	(20.9 - 27.9%)
Paraguay	2016	MICS	2.6%	(1.7 - 3.9%)
Peru	2018	DHS	1.1%	(0.7 - 1.6%)
Philippines	2017	DHS	9.5%	(7.9 - 11.4%)
Rwanda	2014	DHS	0.7%	(0.4 - 1.3%)
São Tomé and Príncipe	2014	MICS	1.9%	(0.9 - 4.0%)
Senegal	2018	DHS	2.4%	(1.5 - 3.8%)
Serbia	2014	MICS	0.6%	(0.2 - 1.8%)
Sierra Leone	2017	MICS	2.7%	(1.9 - 3.7%)
South Africa	2016	DHS	5.3%	(3.4 - 8.0%)
South Sudan	2010	MICS	57.6%	(54.1 - 61.0%)
State of Palestine	2014	MICS	0.1%	(0.0 - 0.5%)

Country	Year	Source	Zero dose prevalence	CI 95%
Sudan	2014	MICS	12.0%	(10.0 - 14.4%)
Tajikistan	2017	DHS	3.5%	(2.6 - 4.7%)
Tanzania	2015	DHS	2.3%	(1.6 - 3.4%)
Thailand	2015	MICS	3.3%	(1.9 - 5.7%)
Timor-Leste	2016	DHS	19.2%	(16.7 - 22.0%)
Togo	2017	MICS	4.6%	(3.2 - 6.7%)
Tunisia	2018	MICS	0.3%	(0.1 - 1.3%)
Turkmenistan	2015	MICS	0.1%	(0.0 - 0.9%)
Uganda	2016	DHS	1.5%	(1.0 - 2.1%)
Ukraine	2012	MICS	3.4%	(2.2 - 5.2%)
Vietnam	2013	MICS	1.5%	(0.9 - 2.7%)
Yemen	2013	DHS	16.3%	(14.6 - 18.1%)
Zambia	2018	DHS	1.3%	(0.8 - 2.1%)
Zimbabwe	2019	MICS	5.1%	(3.6 - 7.2%)

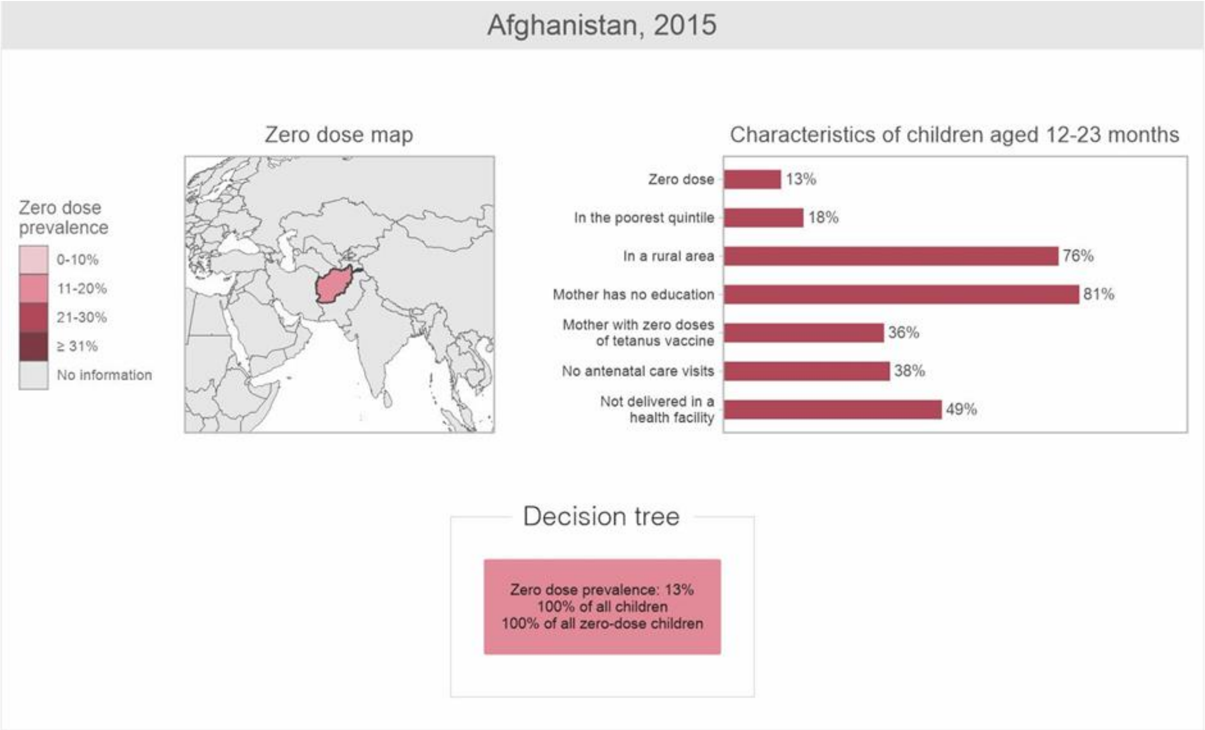

Figure S1. – Afghanistan’s country profile.

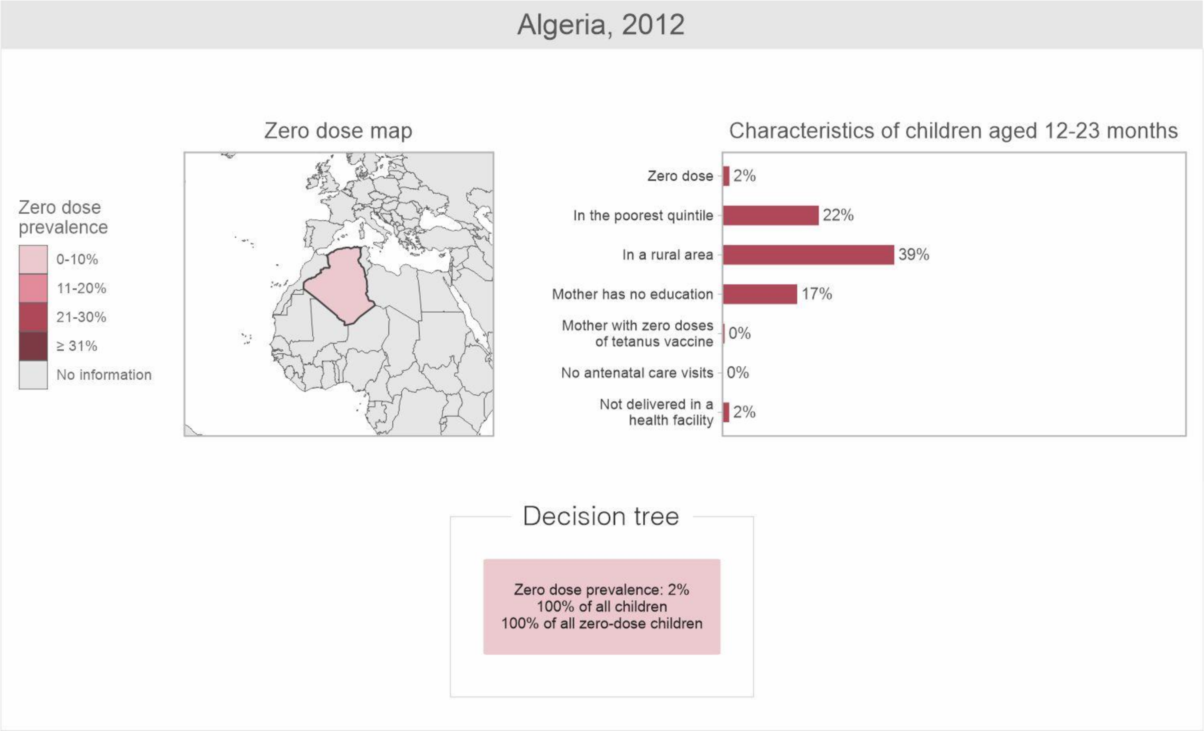

Figure S2. – Algeria's country profile.

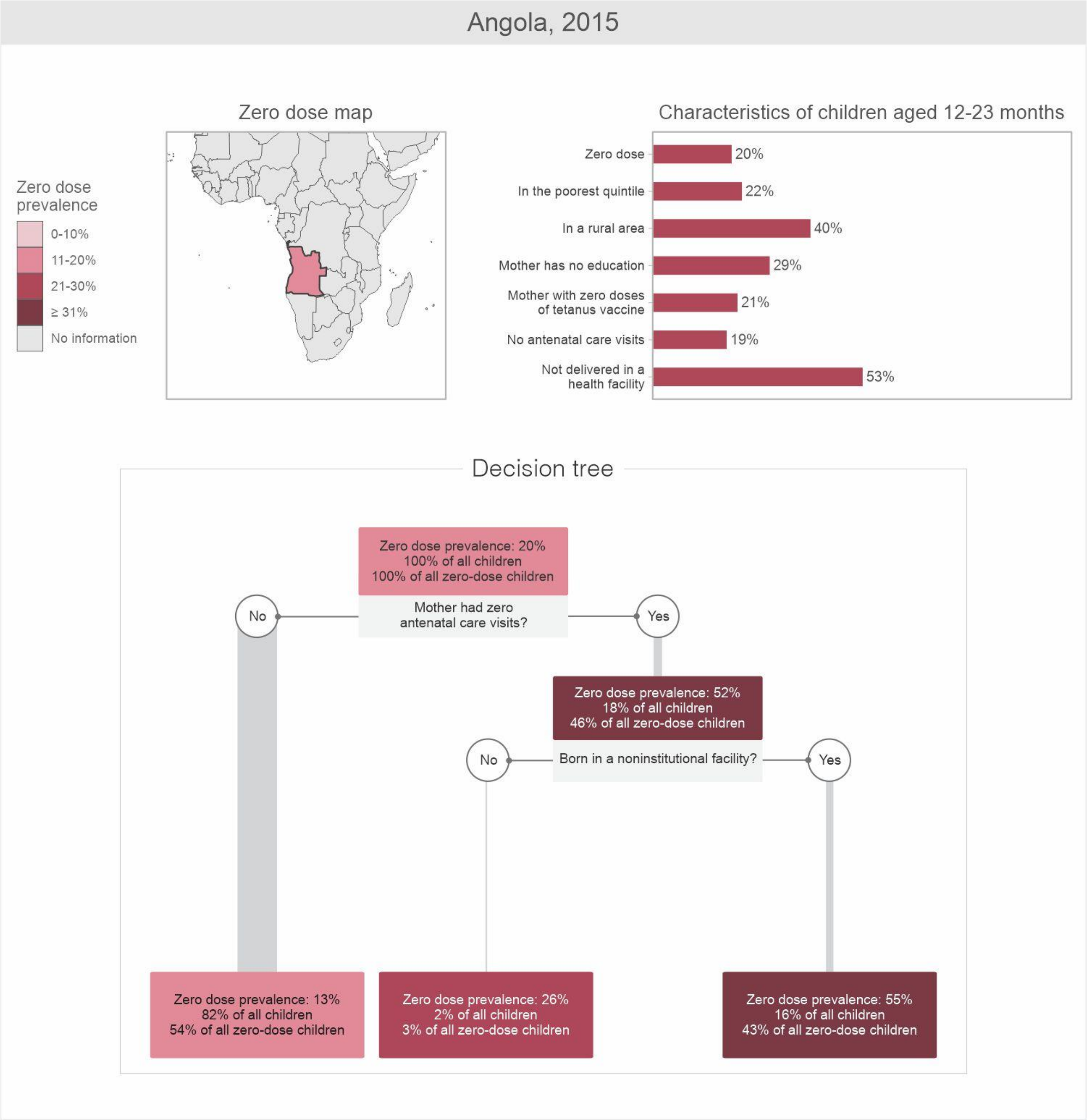

Figure S3. – Angola's country profile.

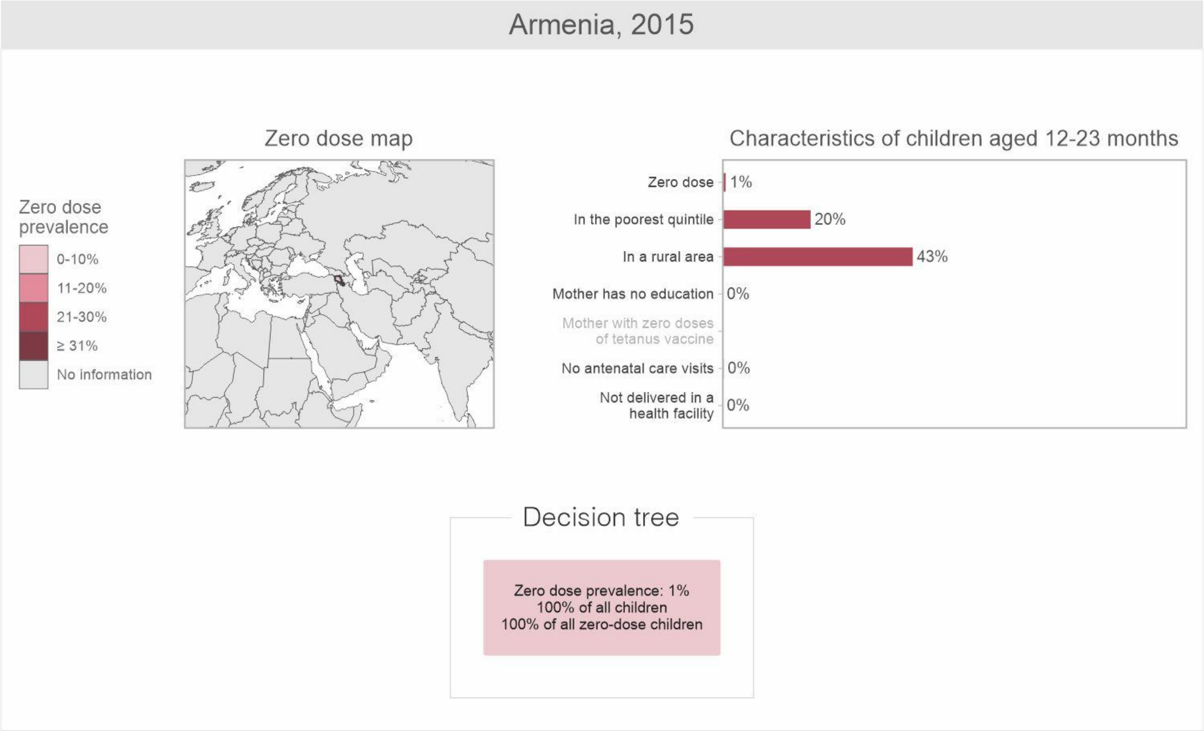

Figure S4. – Armenia's country profile.

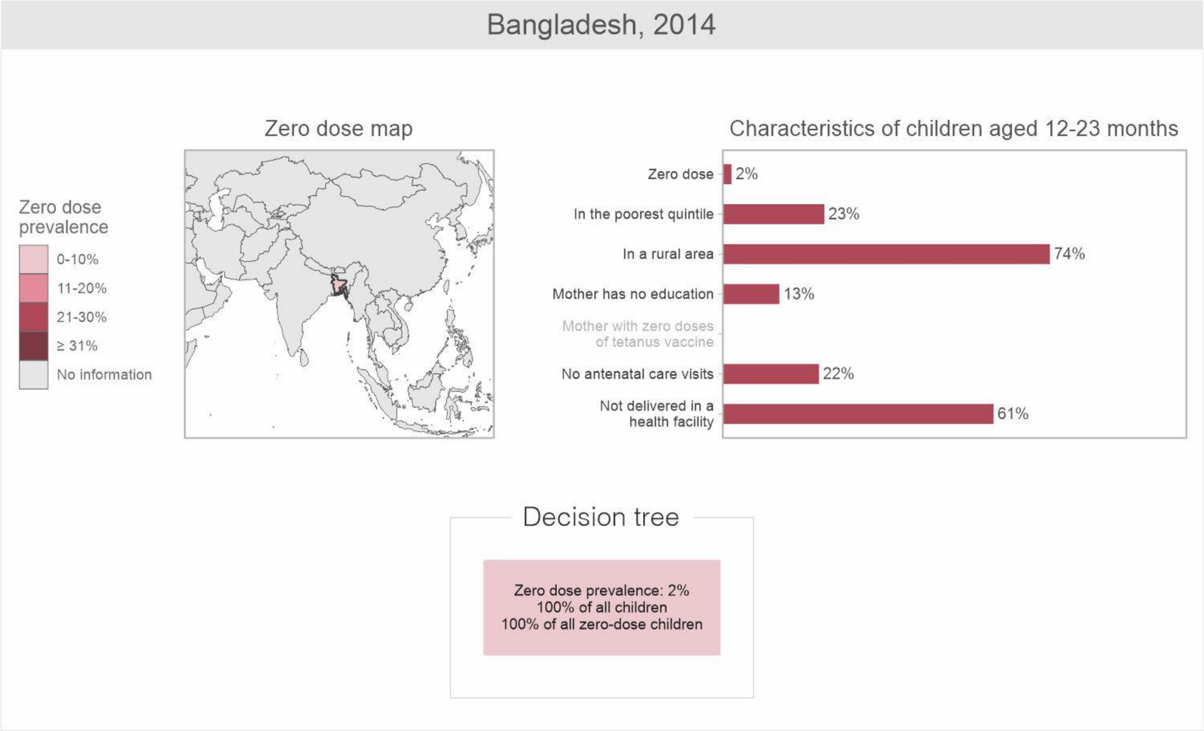

Figure S5. – Bangladesh's country profile.

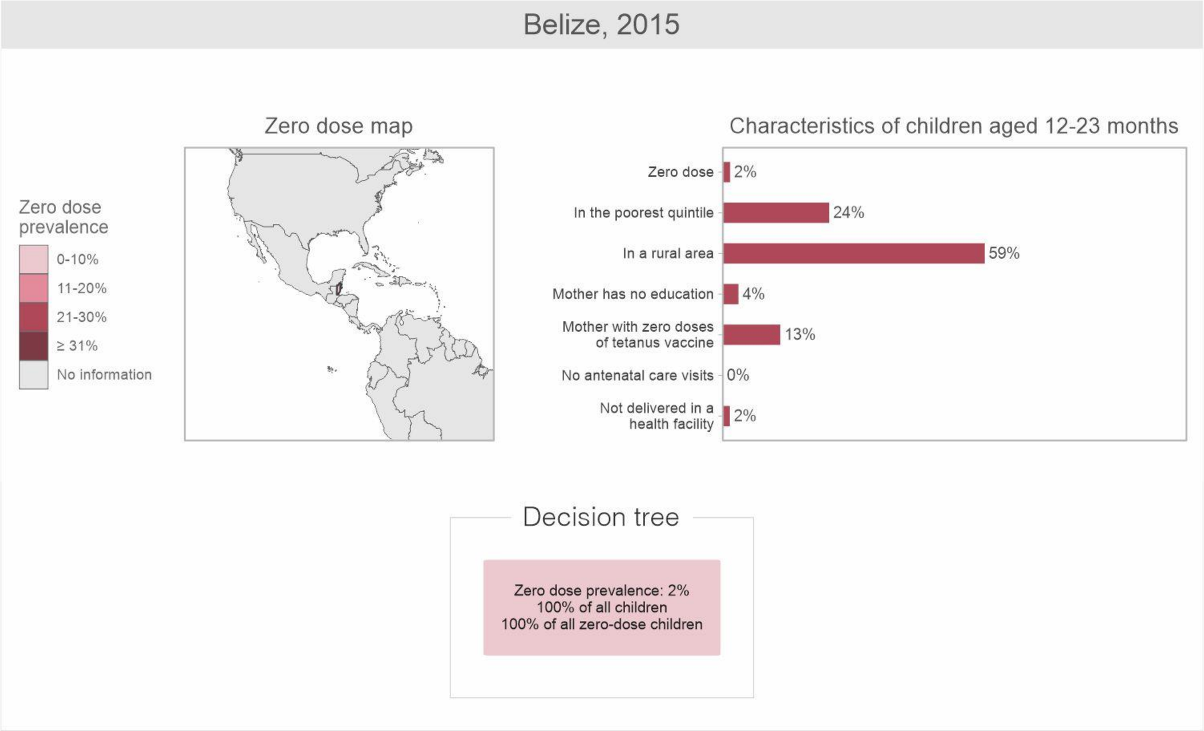

Figure S6. – Belize's country profile.

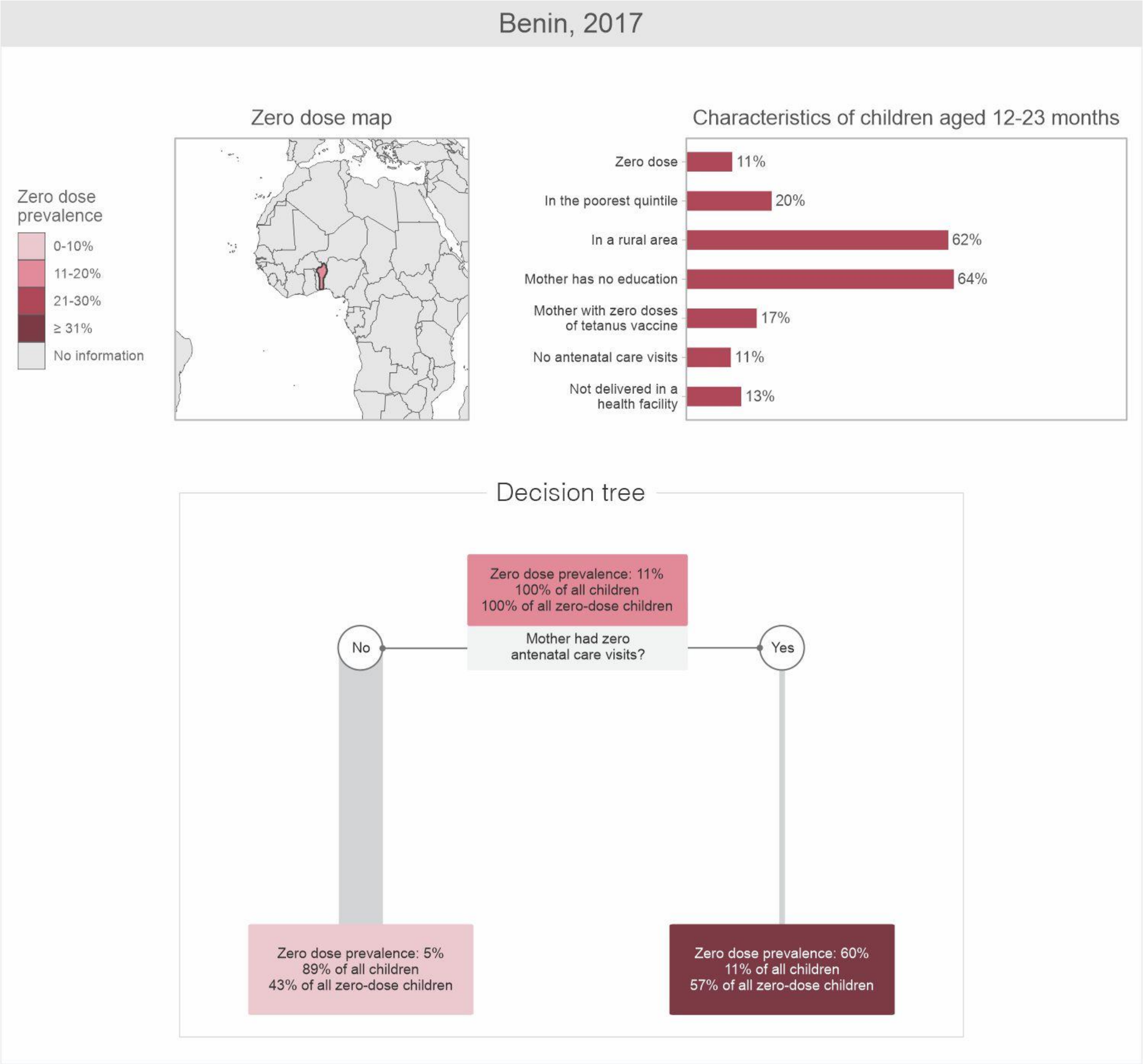

Figure S7. – Benin's country profile.

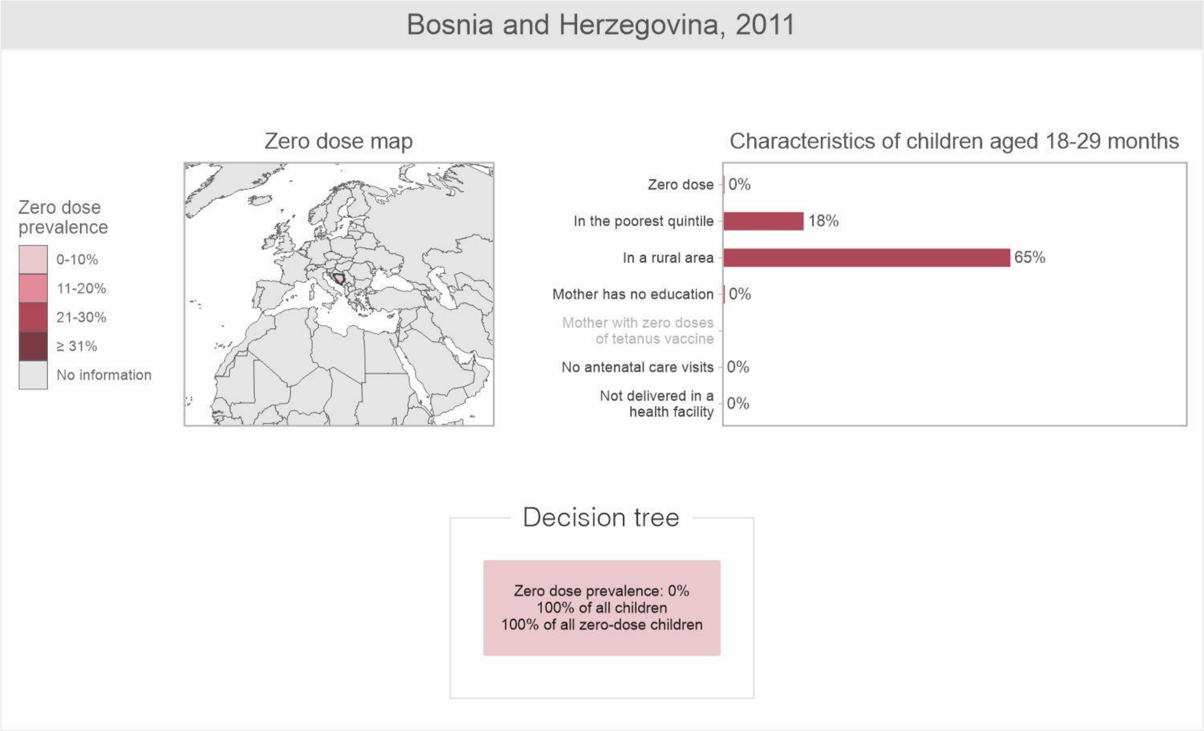

Figure S8. – Bosnia and Herzegovina's country profile.

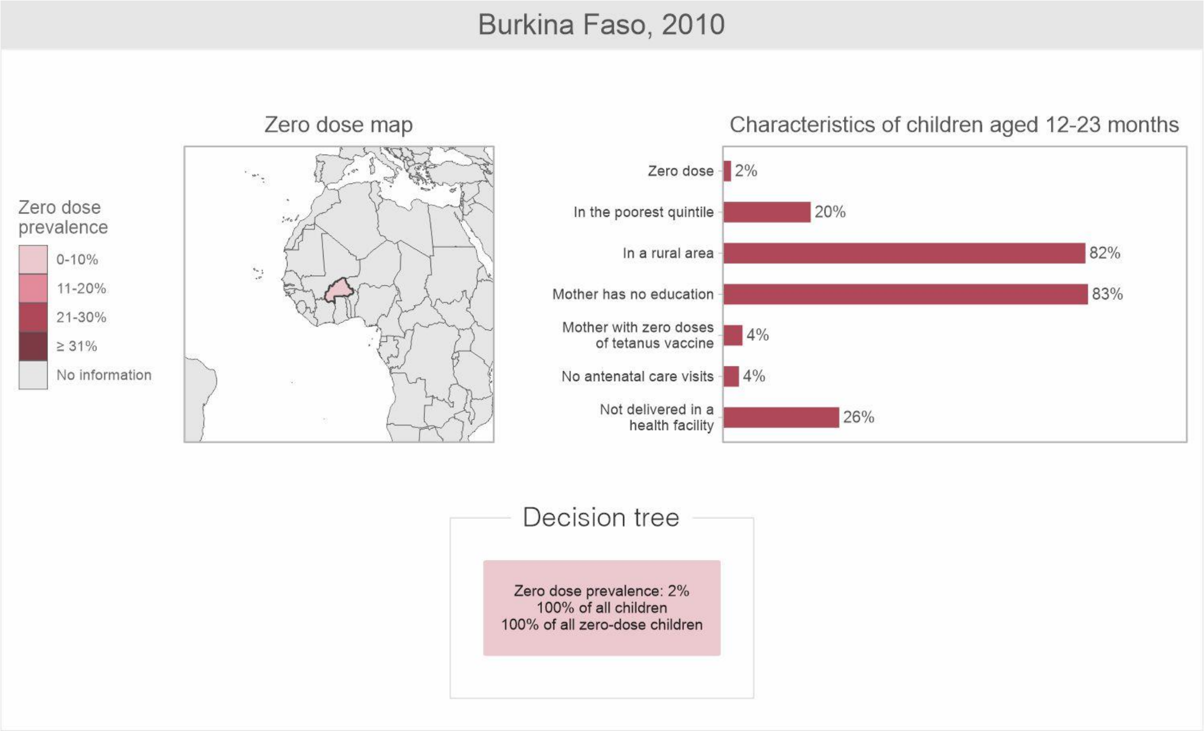

Figure S9. – Burkina Faso's country profile.

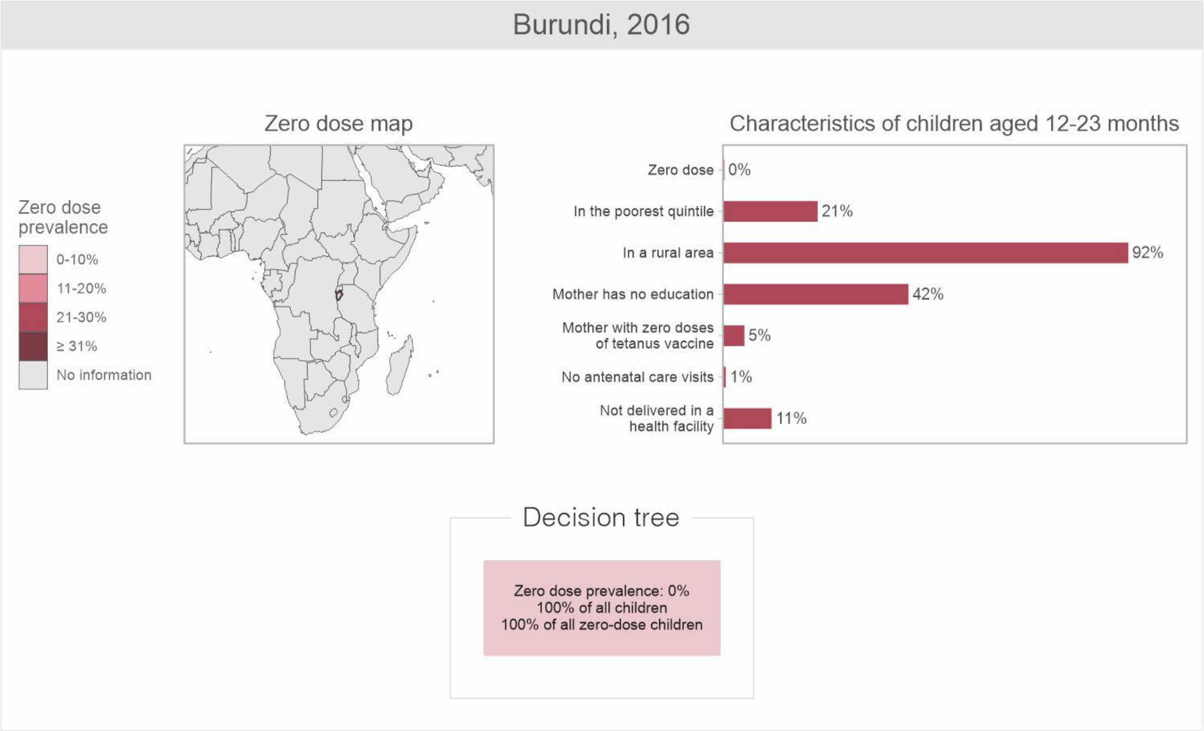

Figure S10. – Burundi's country profile.

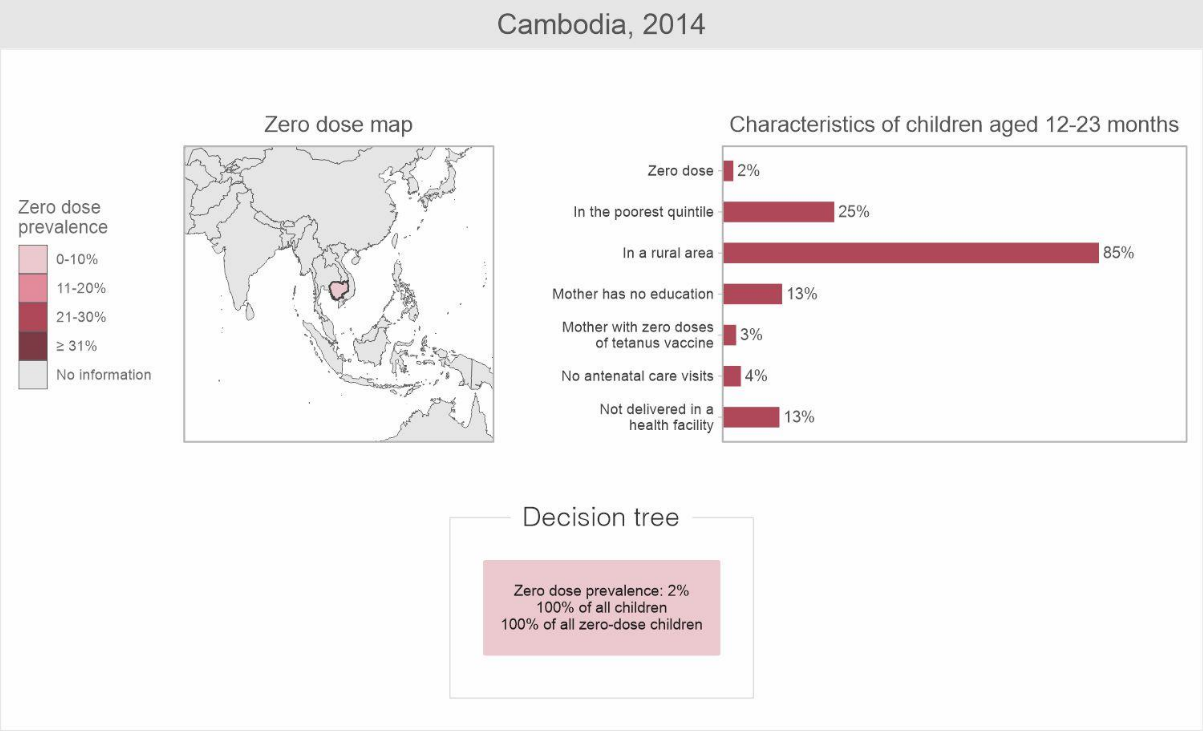

Figure S11. – Cambodia's country profile.

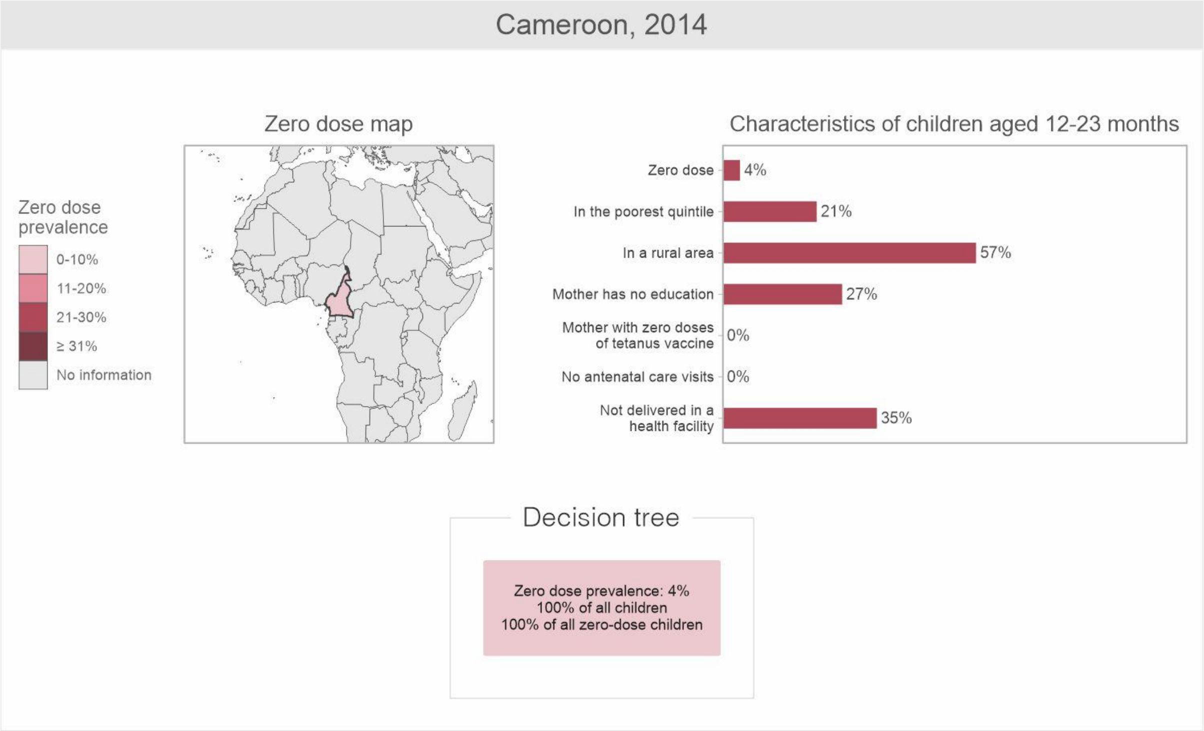

Figure S12. – Cameroon's country profile.

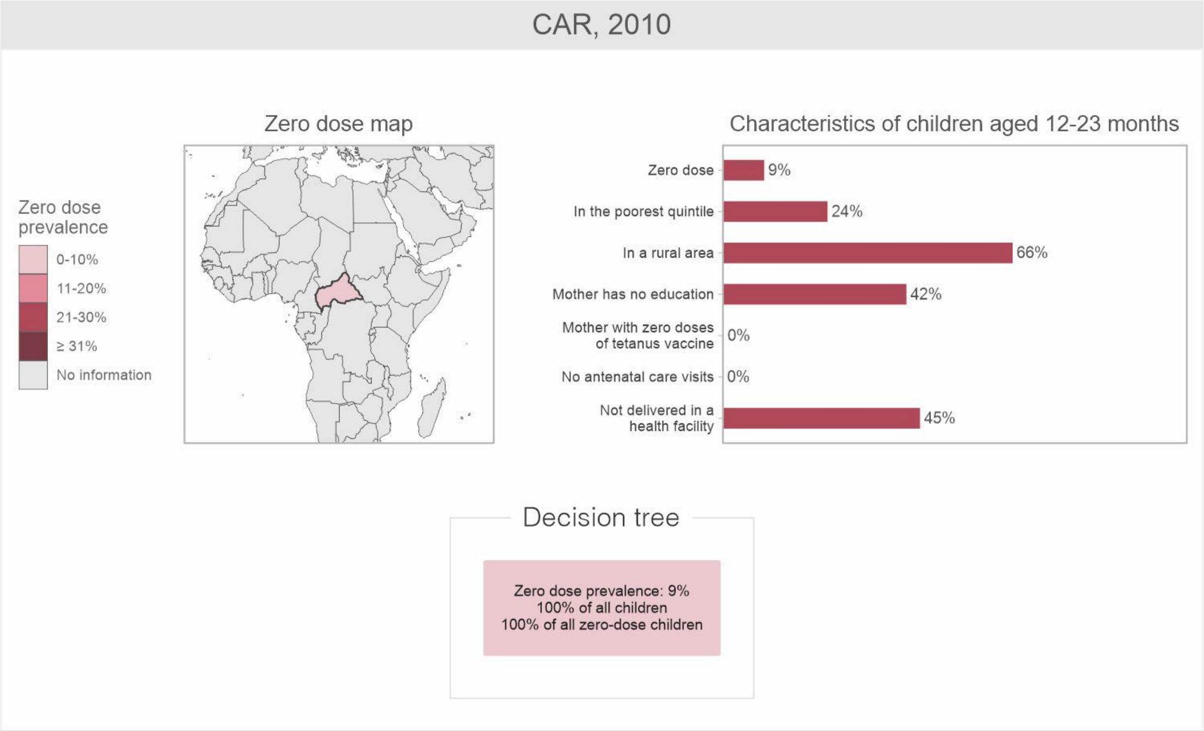

Figure S13. – Central African Republic's country profile.

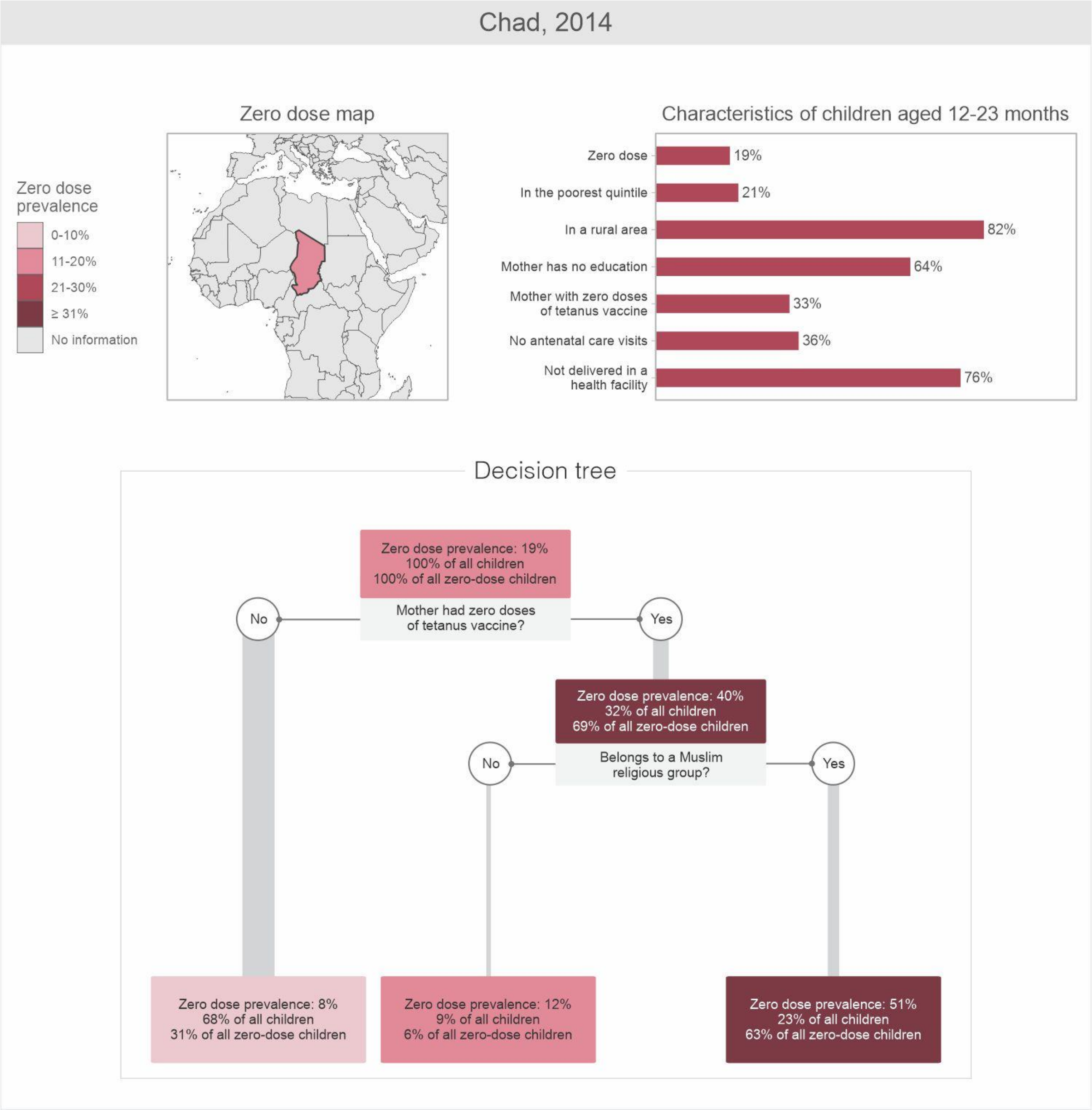

Figure S14. – Chad's country profile.

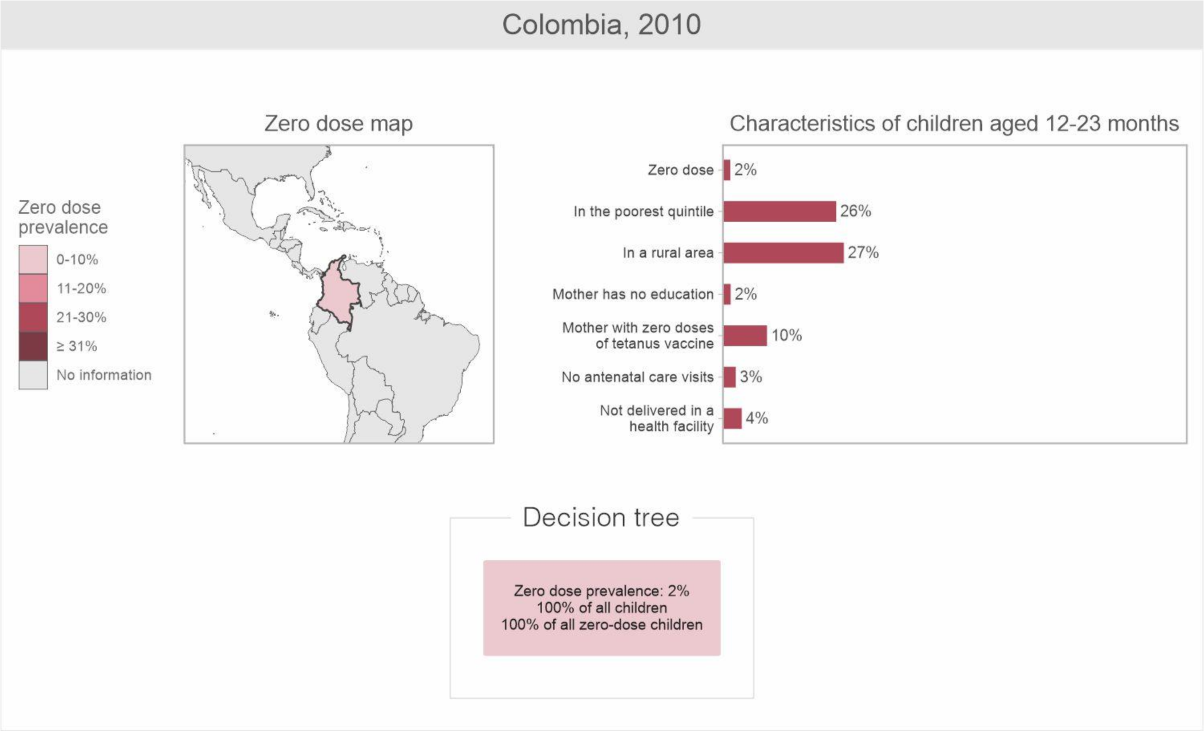

Figure S15. – Colombia's country profile.

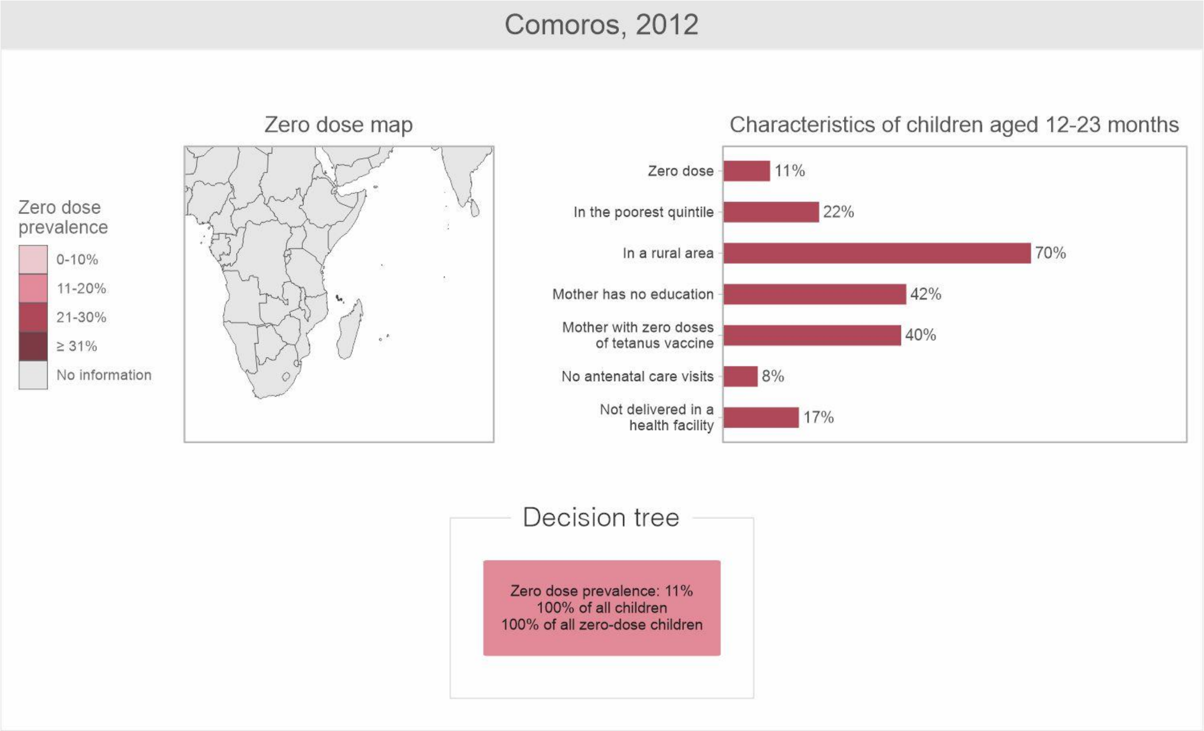

Figure S16. – Comoro's country profile.

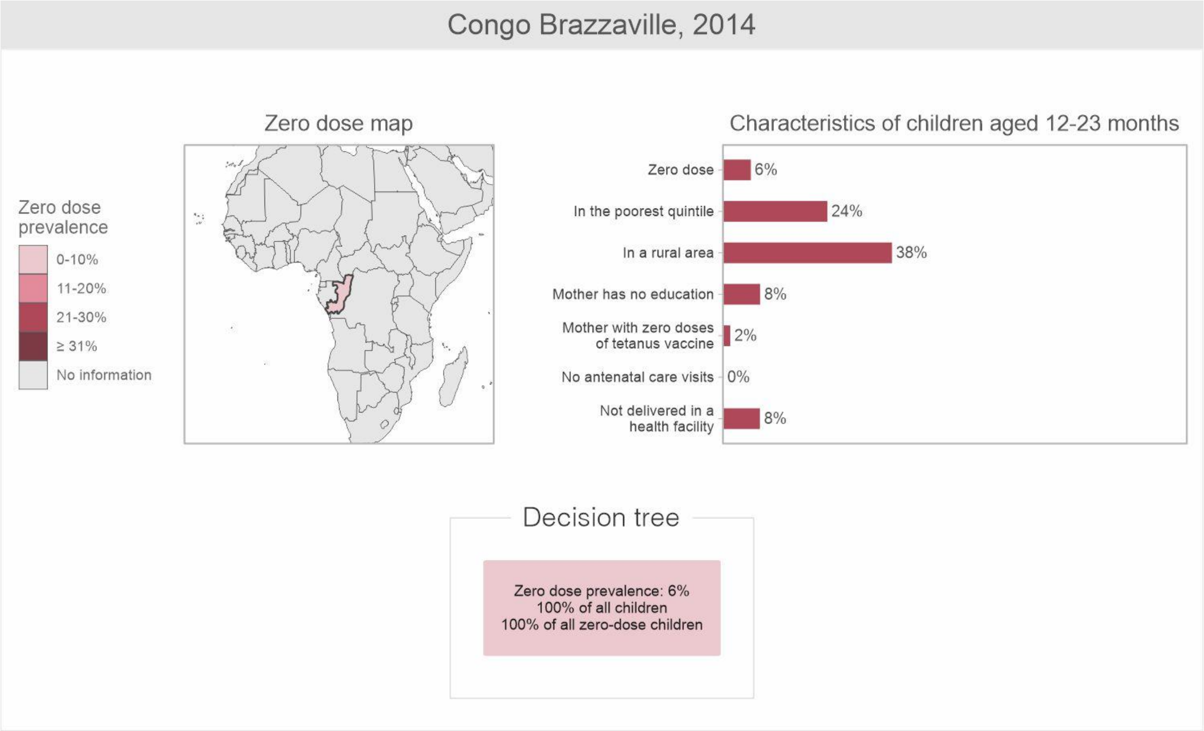

Figure S17. – Congo Brazzaville's country profile.

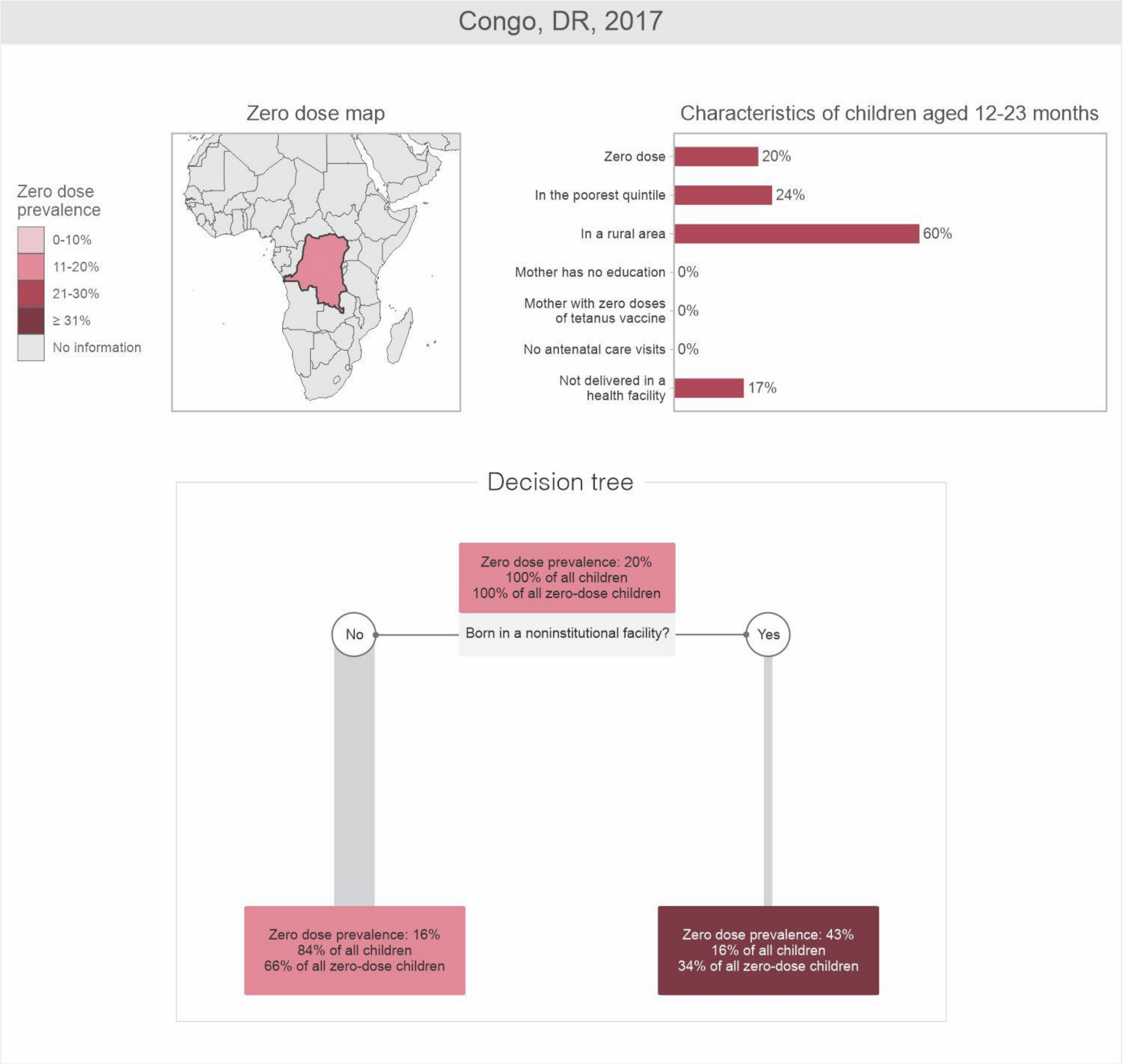

Figure S18. – Congo, Democratic Republic's country profile.

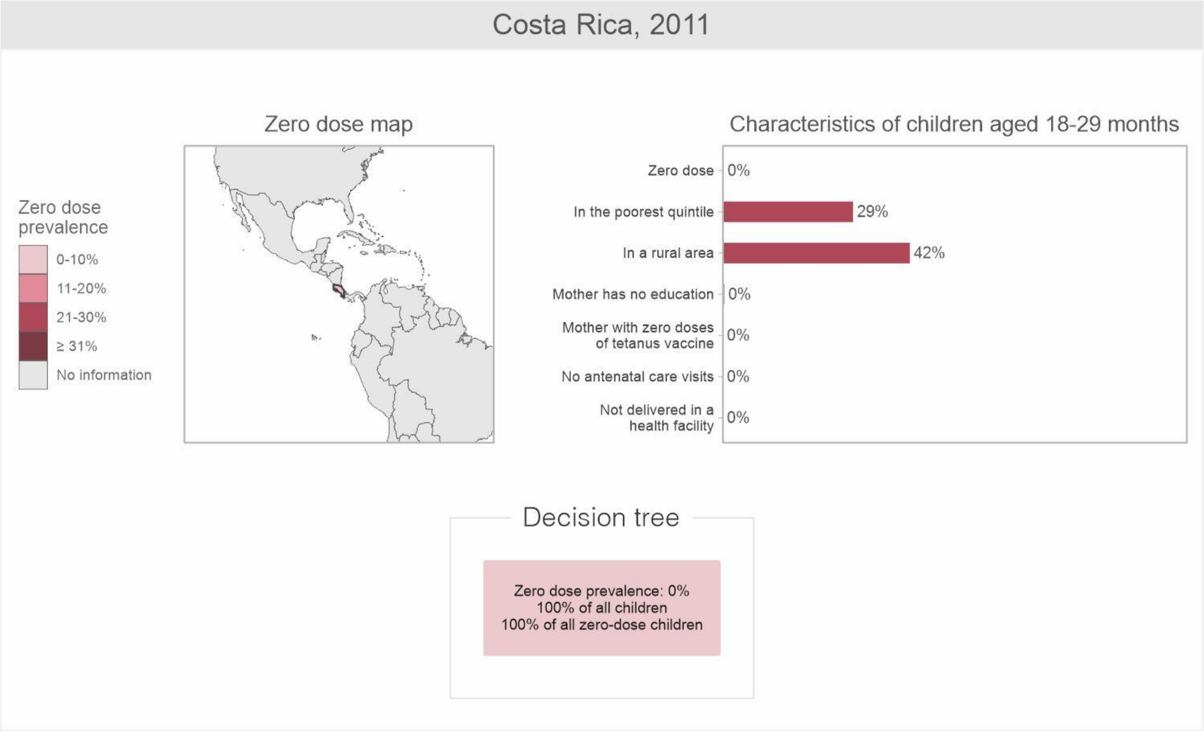

Figure S19. – Costa Rica's country profile.

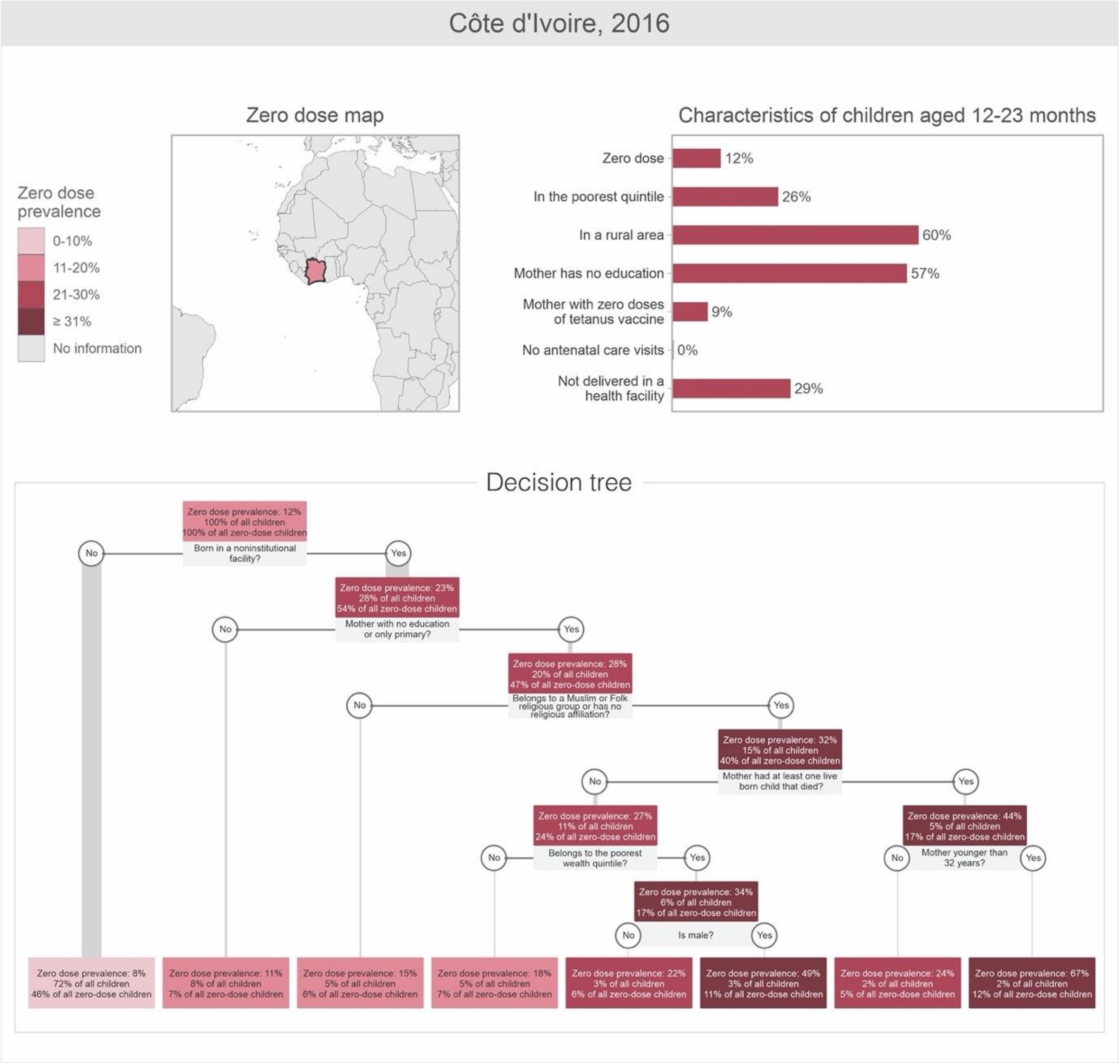

Figure S20. – Côte d'Ivoire country profile.

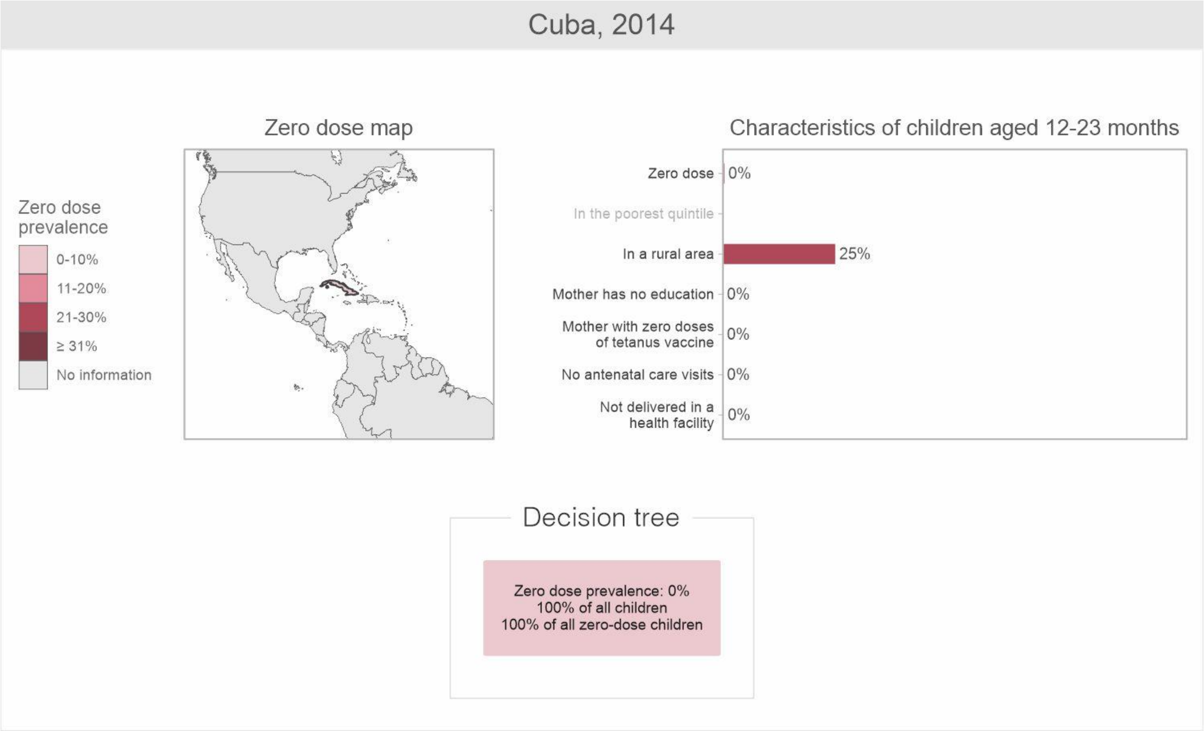

Figure S21. – Cuba's country profile.

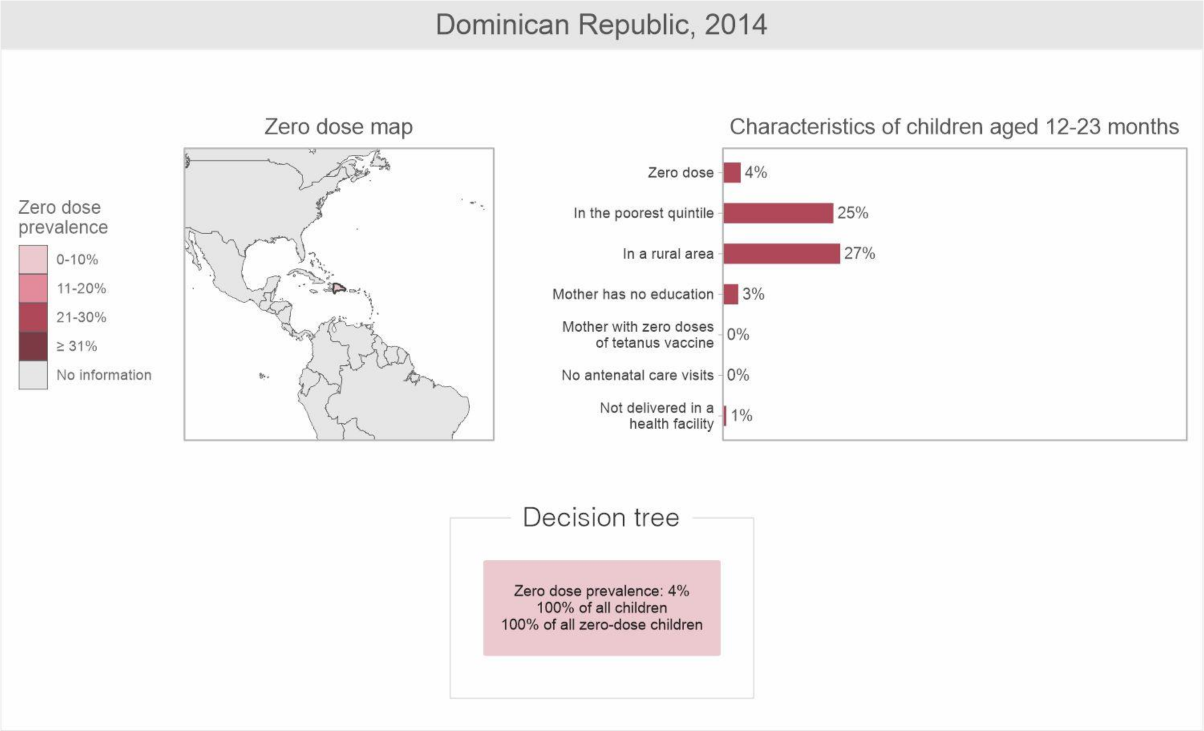

Figure S22. – Dominican Republic's country profile.

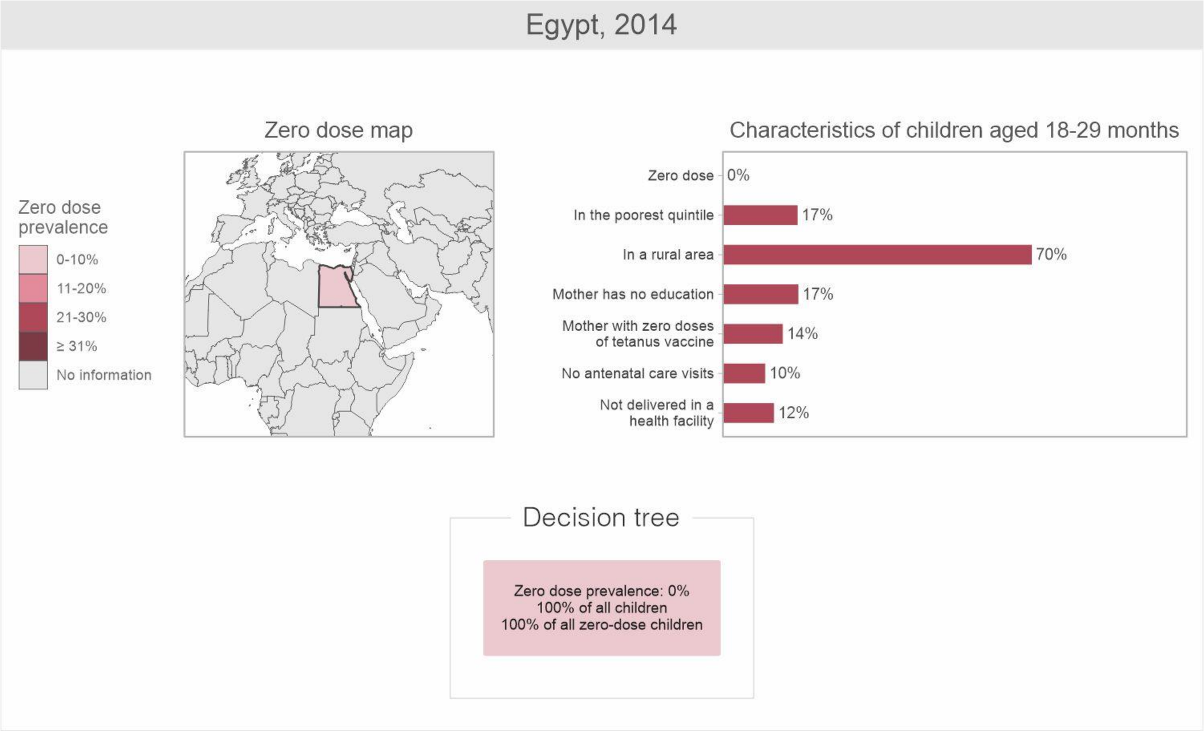

Figure S23. – Egypt's country profile.

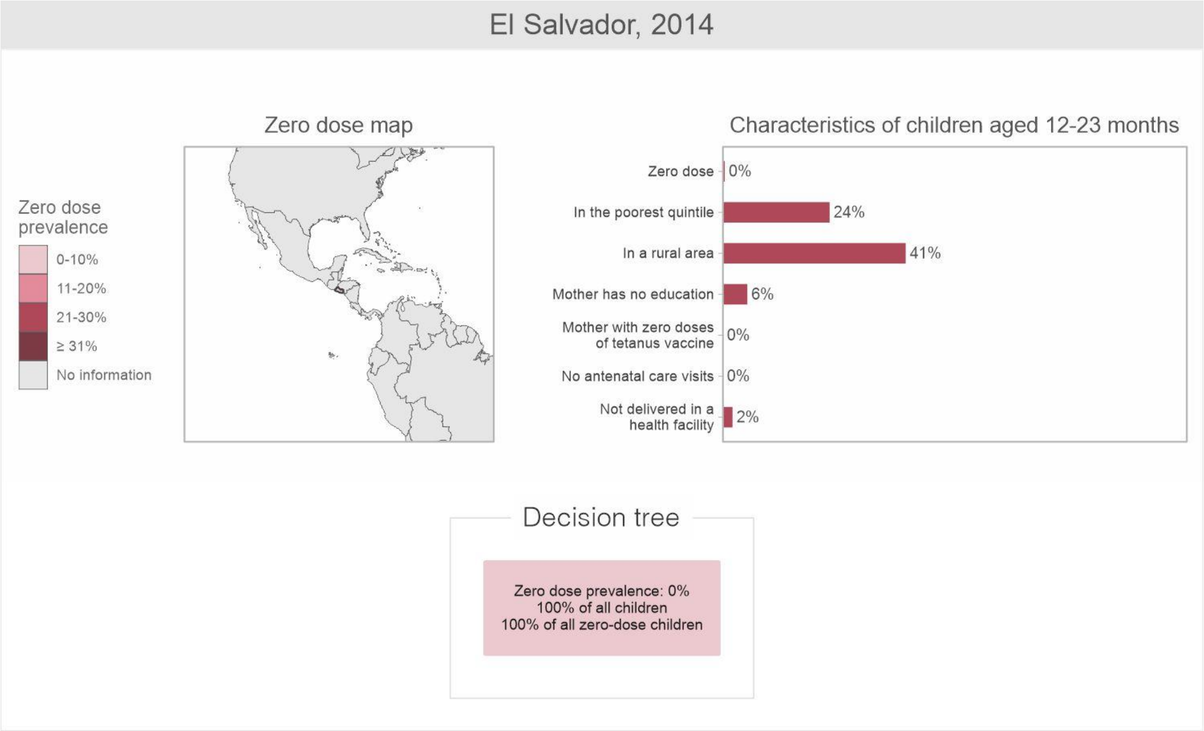

Figure S24. – El Salvador's country profile.

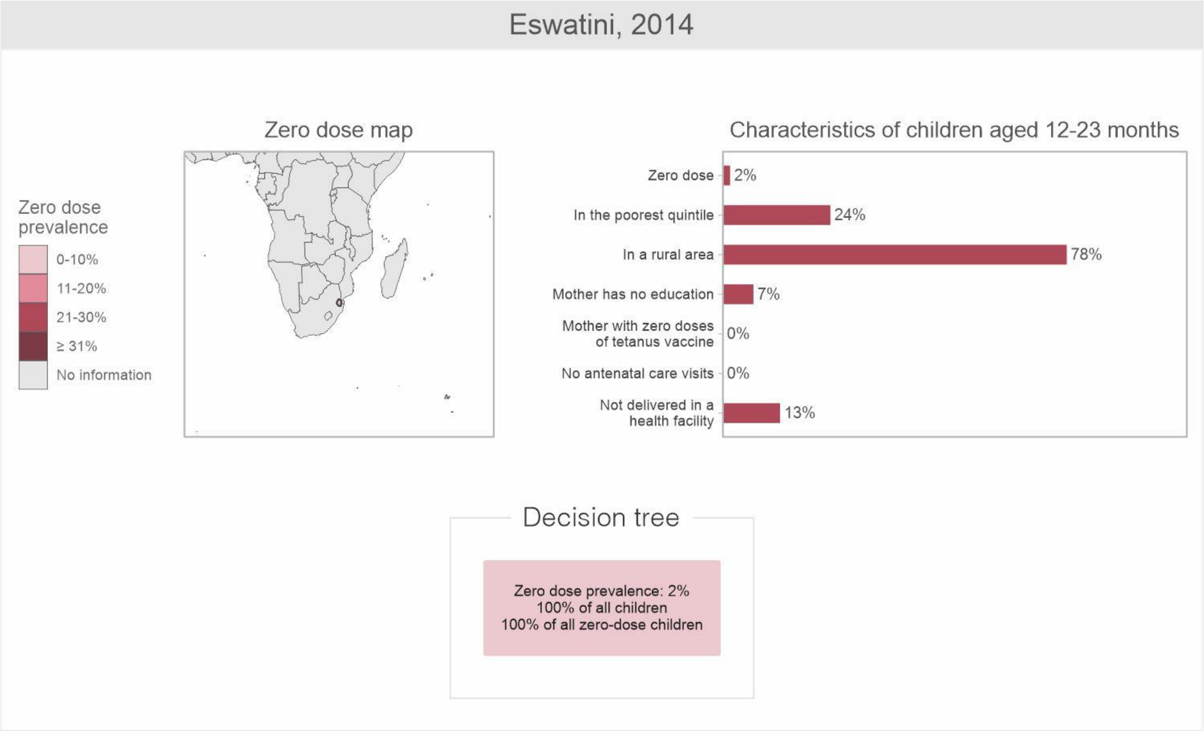

Figure S25. – Eswatini's country profile.

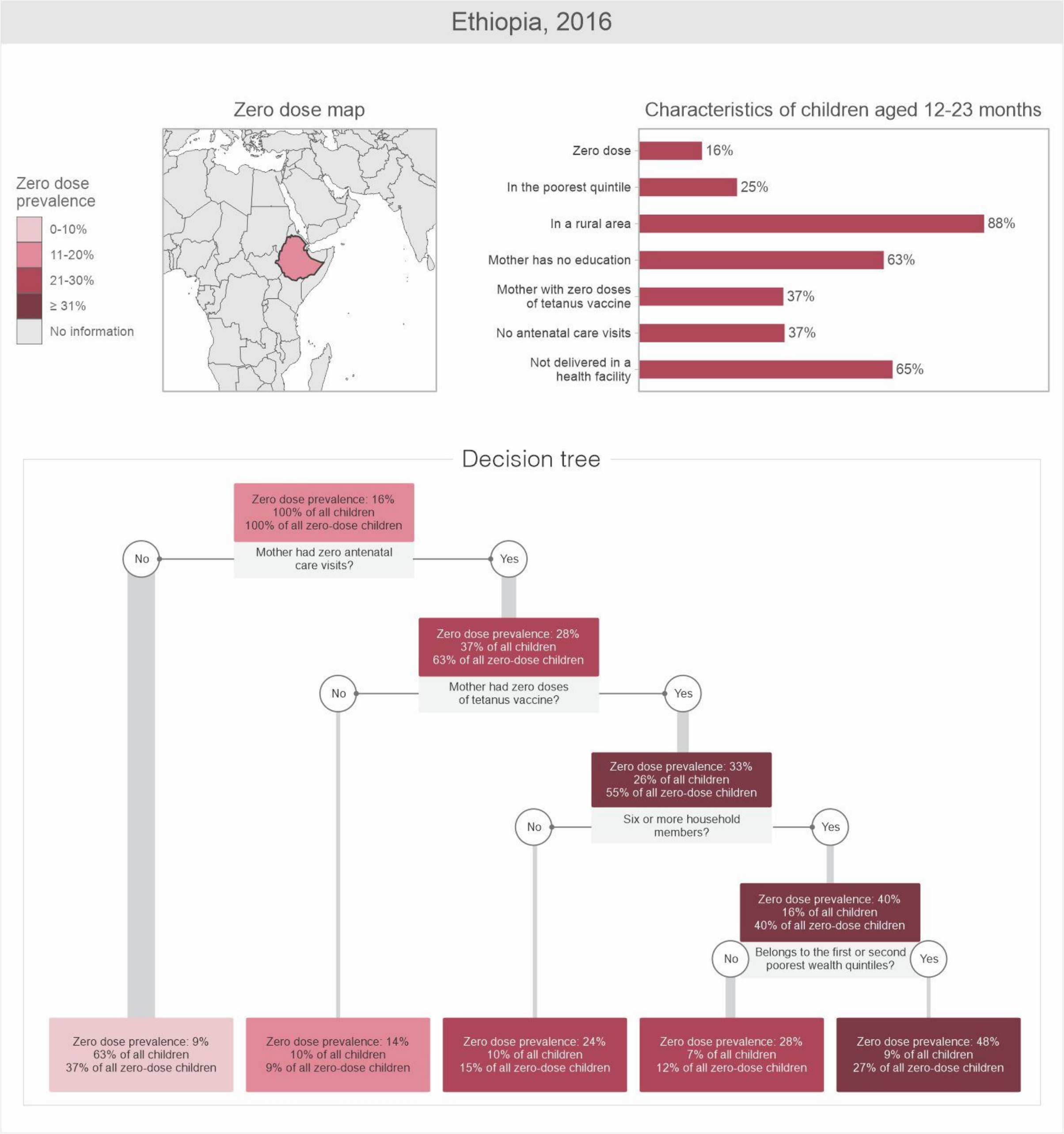

Figure S26. – Ethiopia's country profile.

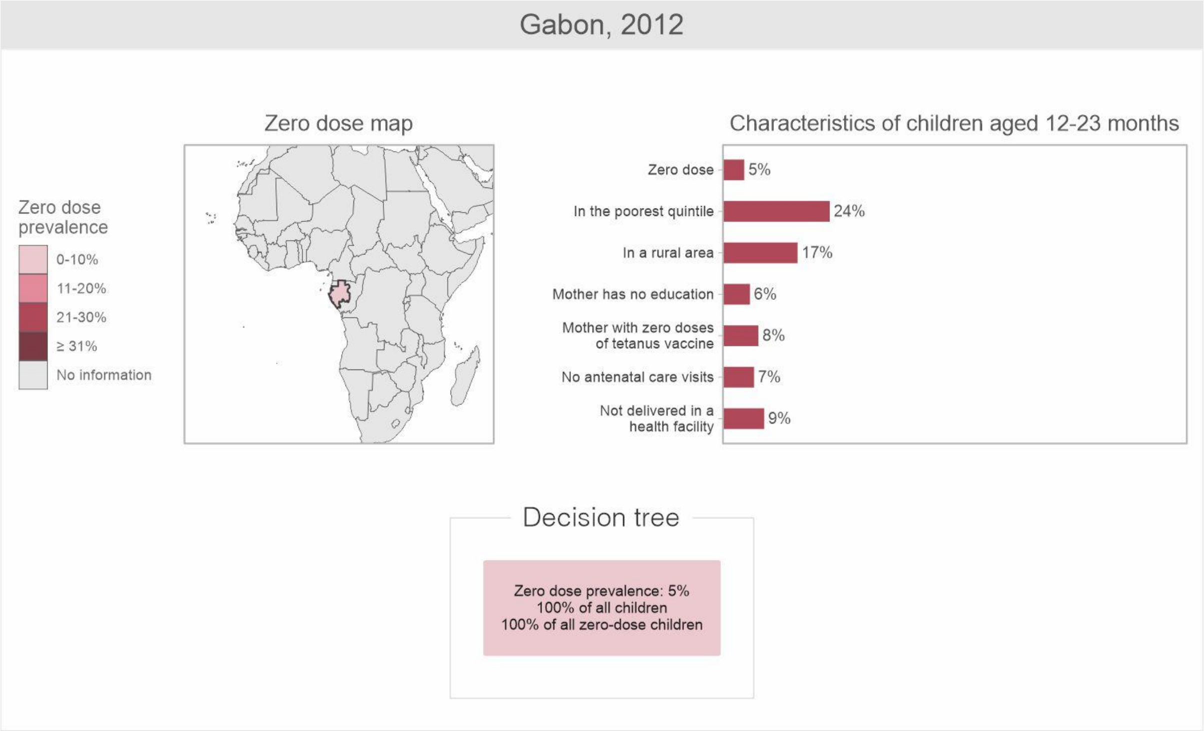

Figure S27. – Gabon's country profile.

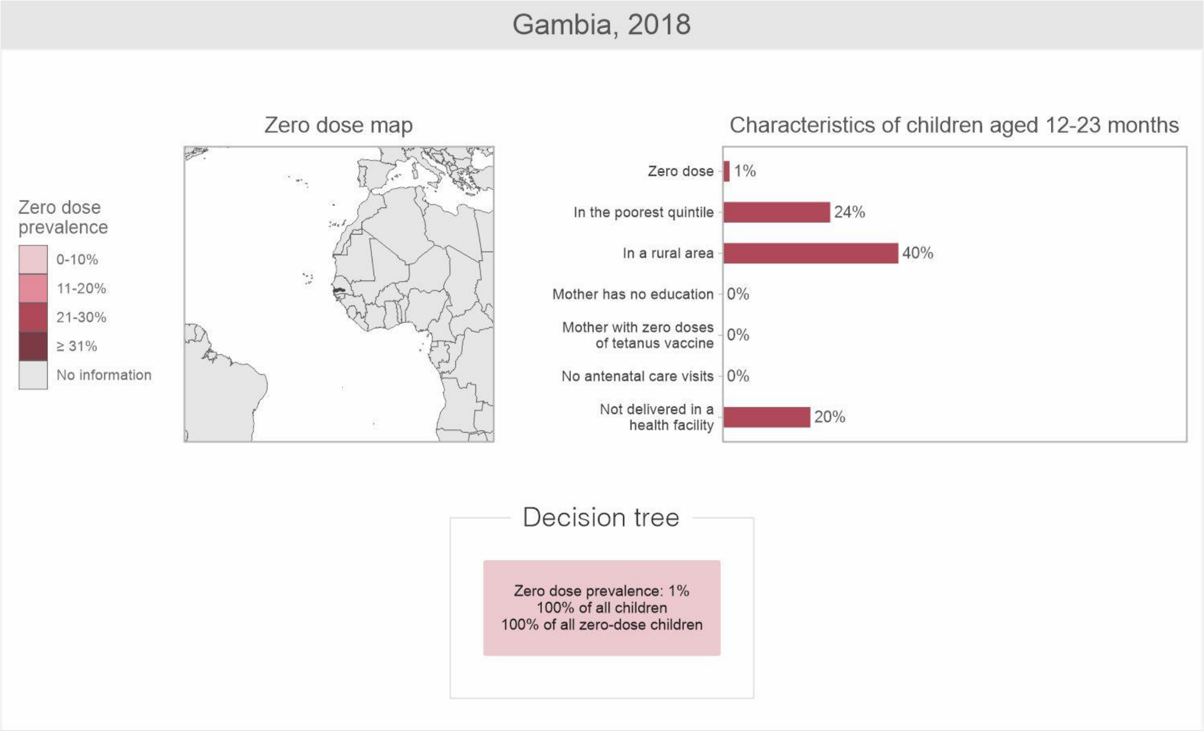

Figure S28. – Gambia's country profile.

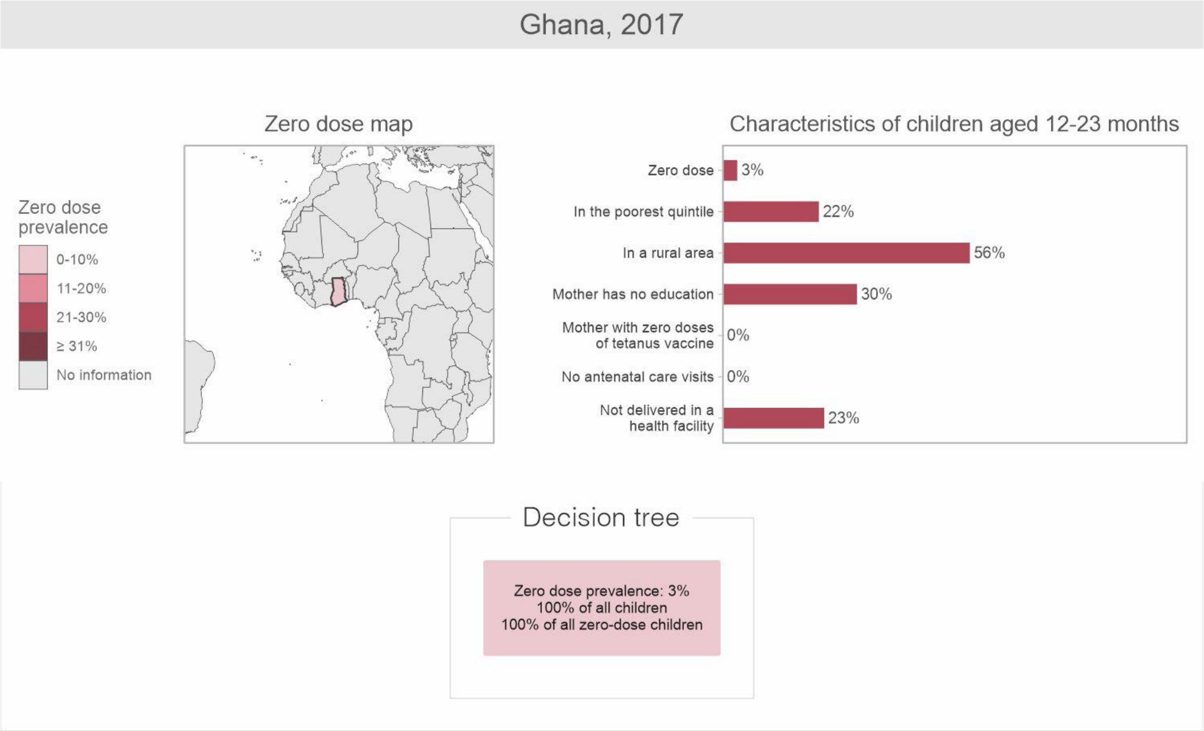

Figure S29. – Ghana's country profile.

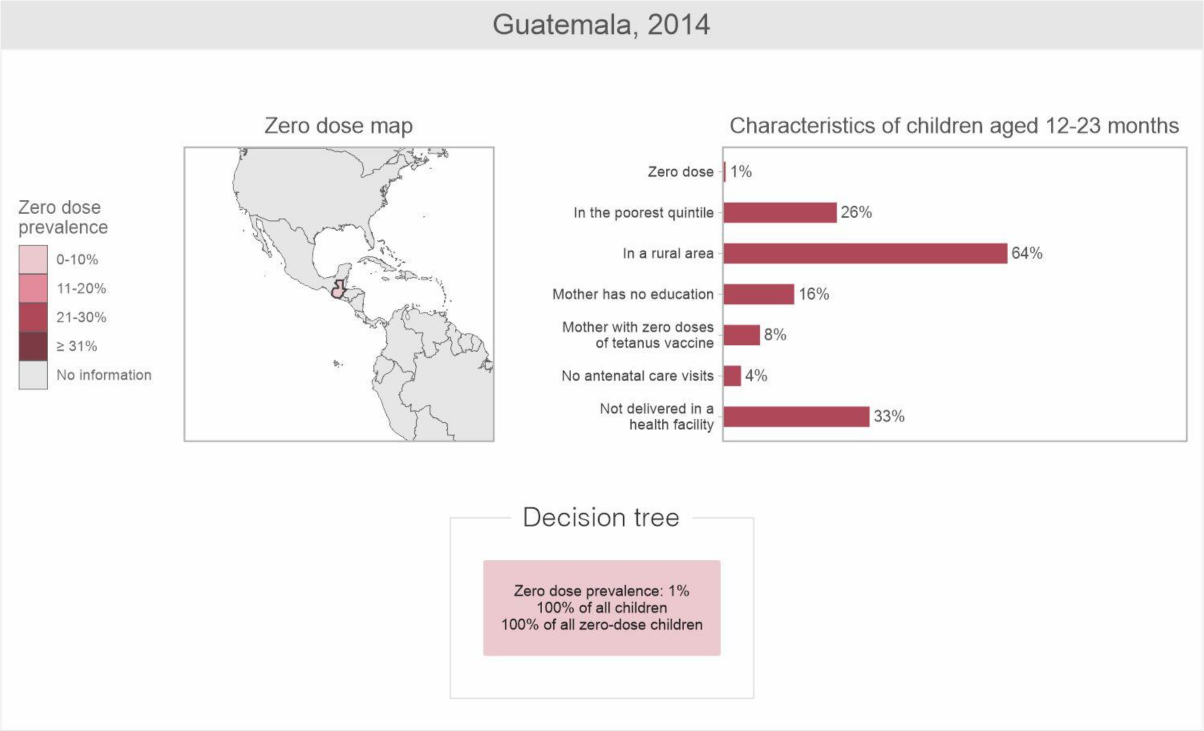

Figure S30. – Guatemala's country profile.

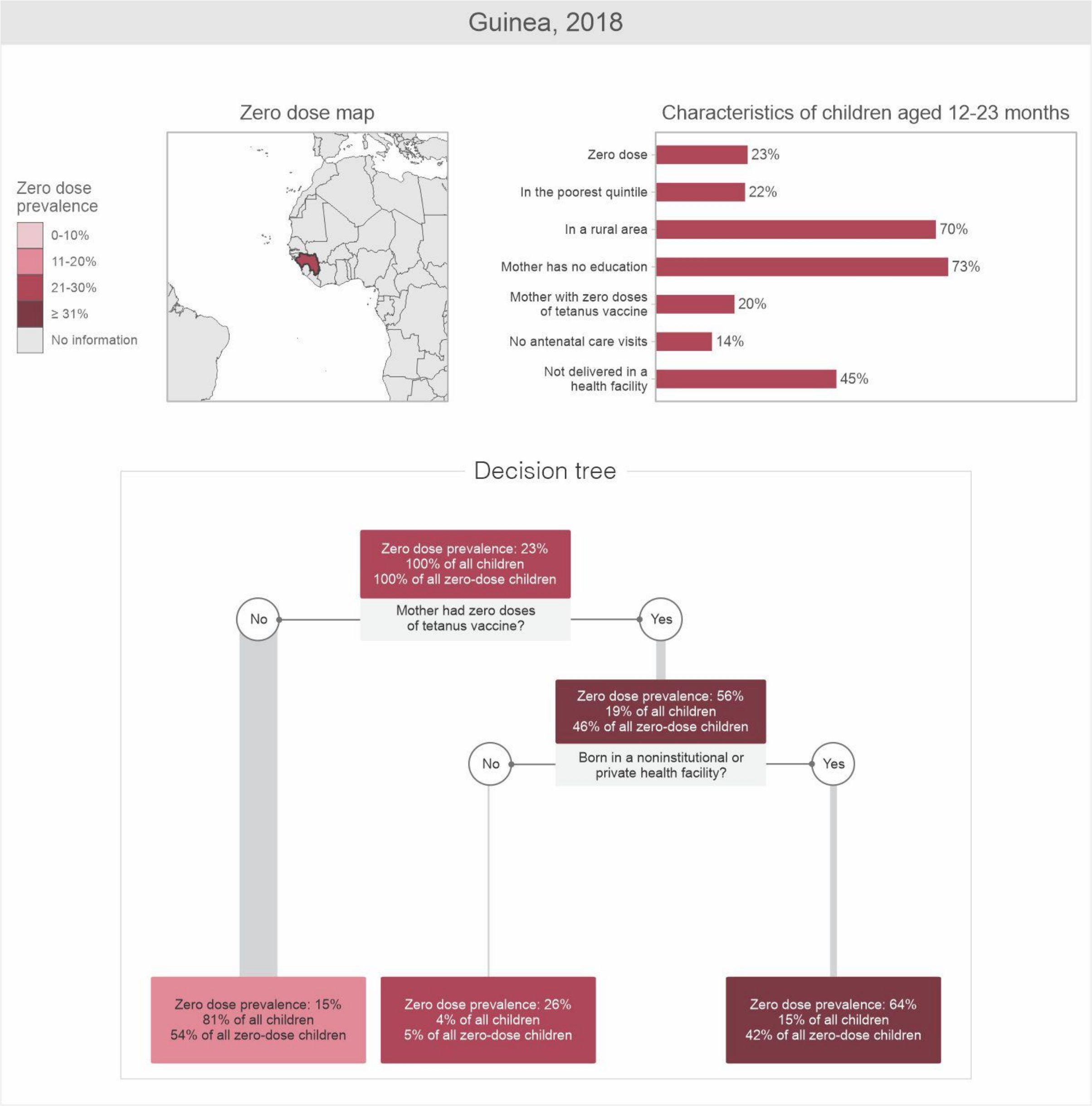

Figure S31. – Guinea's country profile.

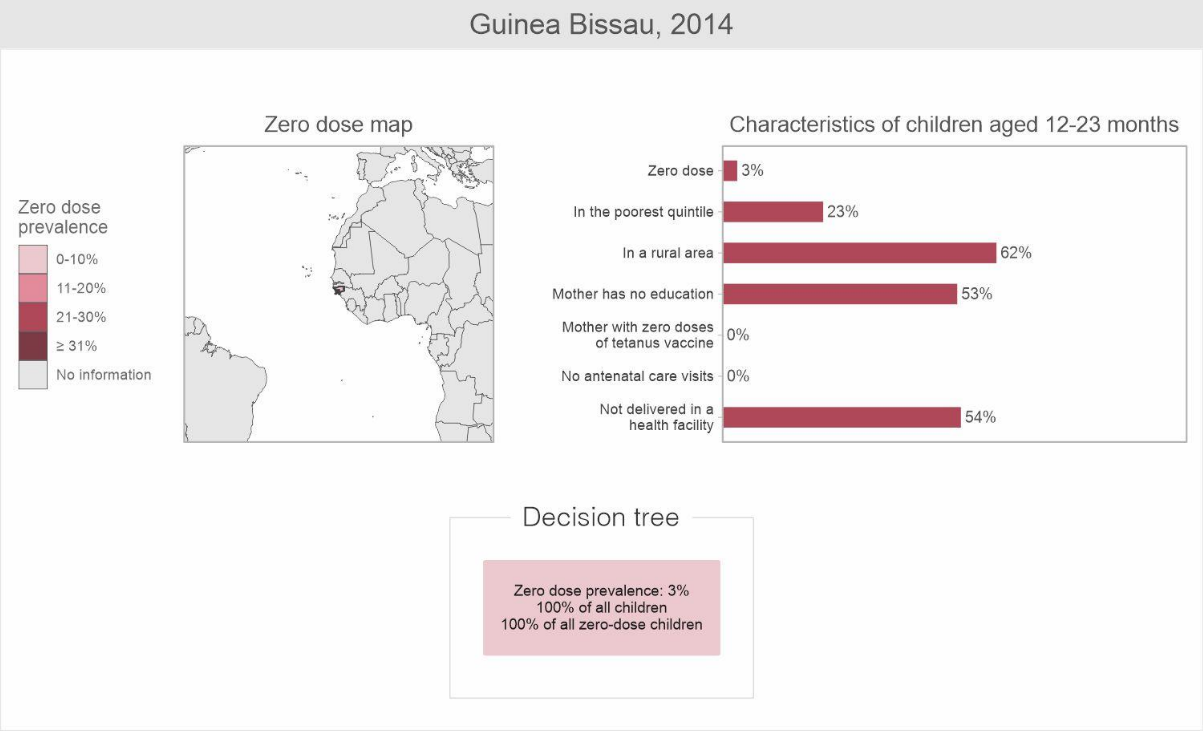

Figure S32. – Guinea Bissau's country profile.

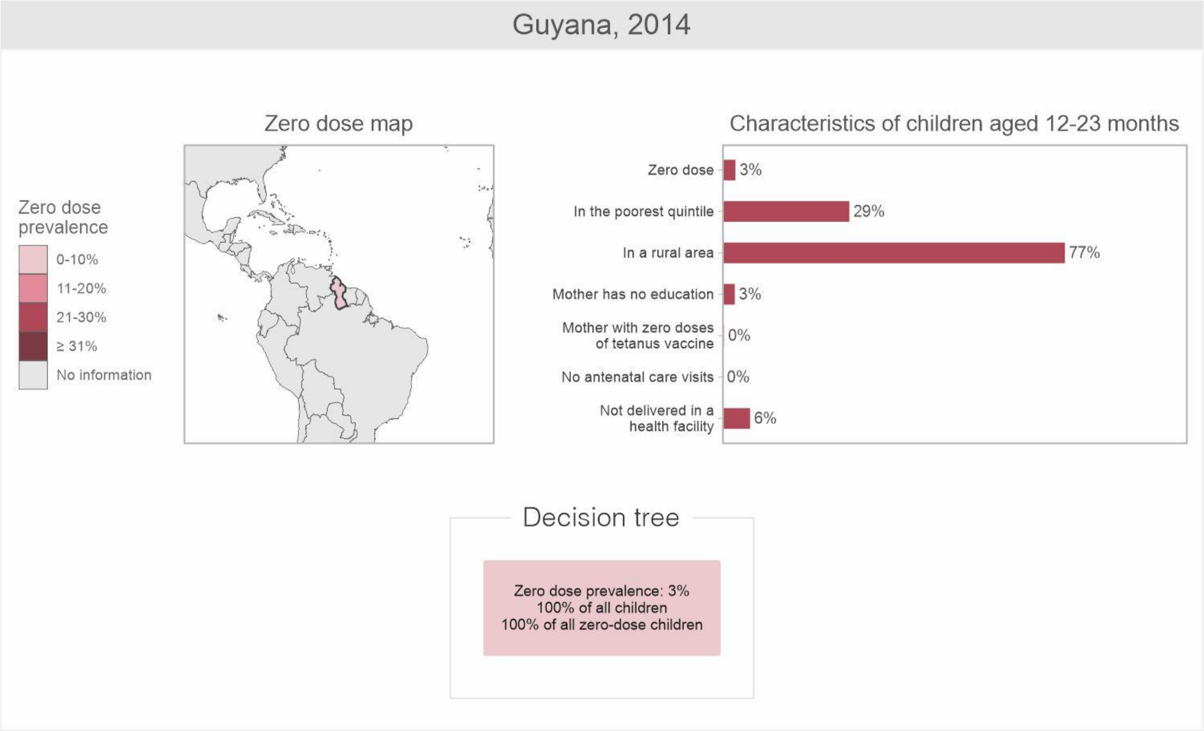

Figure S33. – Guyana's country profile.

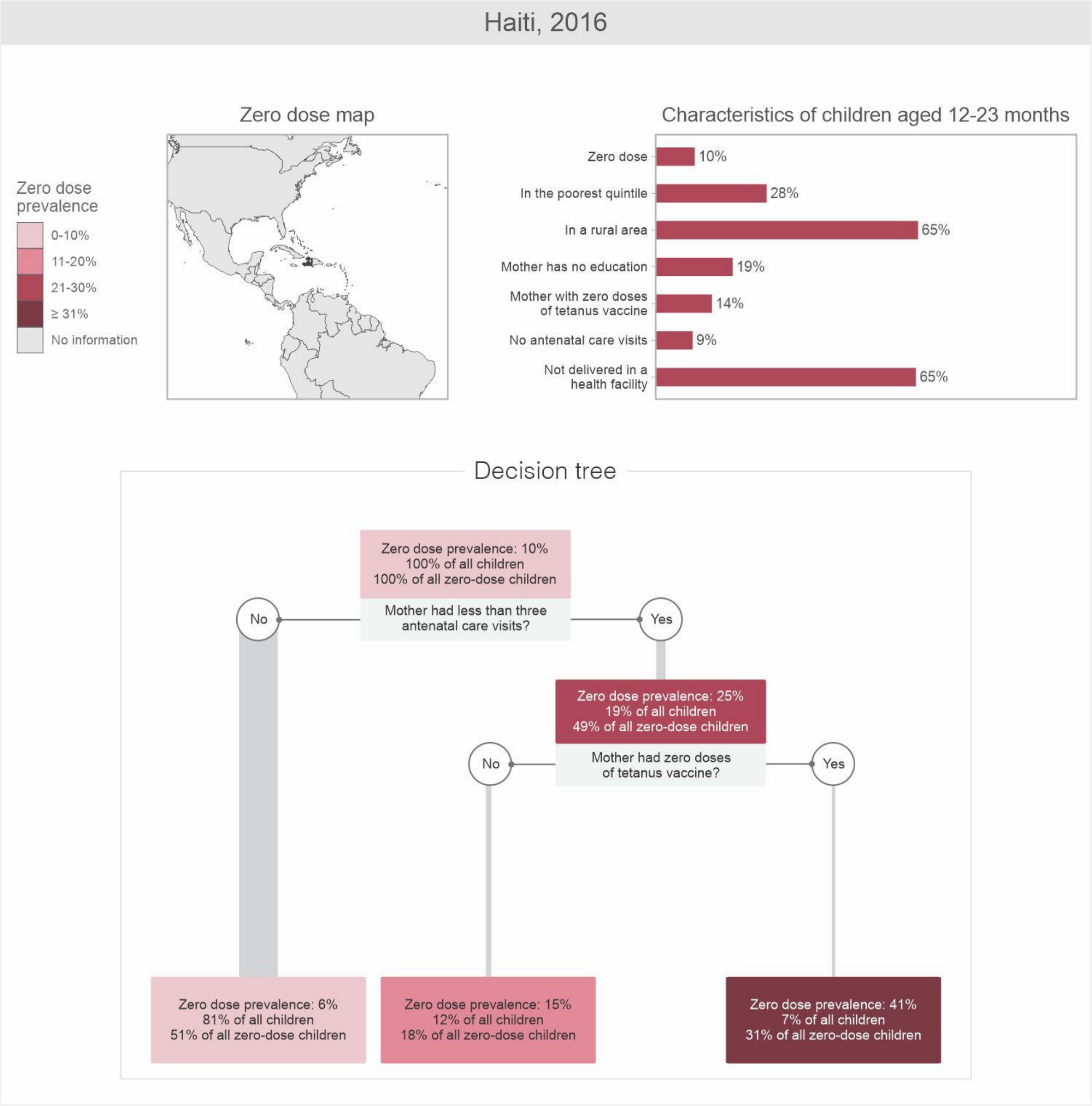

Figure S34. – Haiti's country profile.

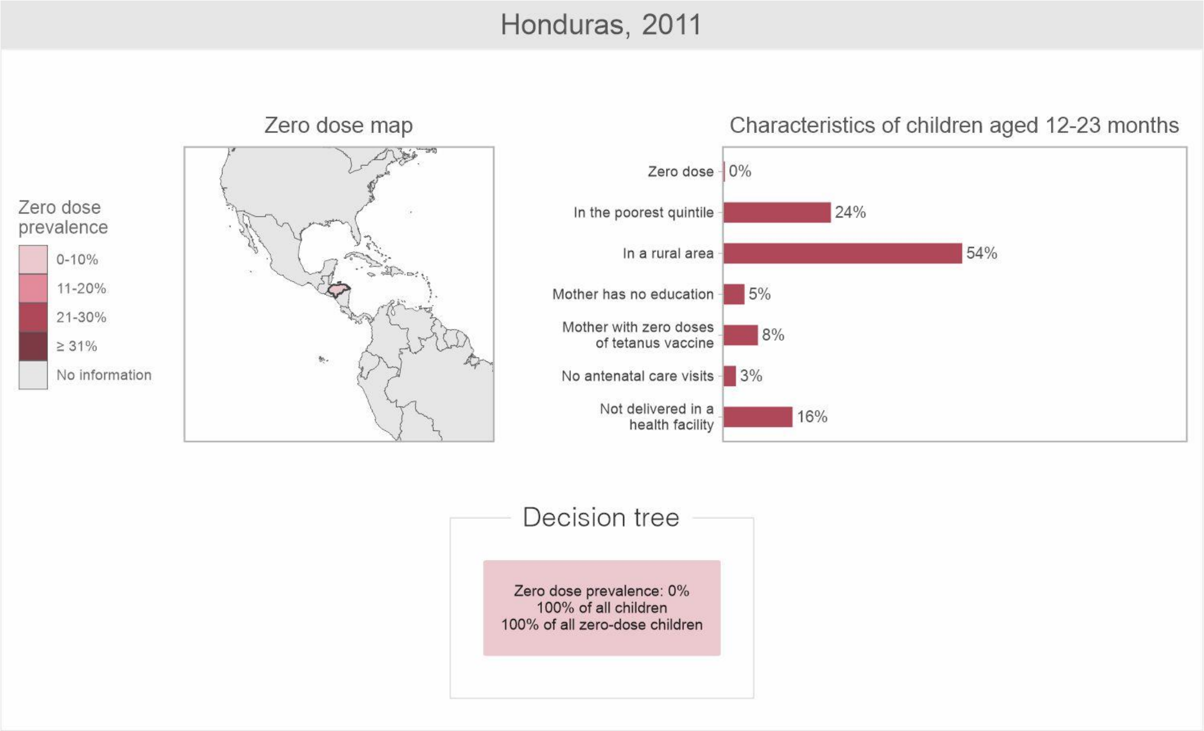

Figure S35. – Hondura's country profile.

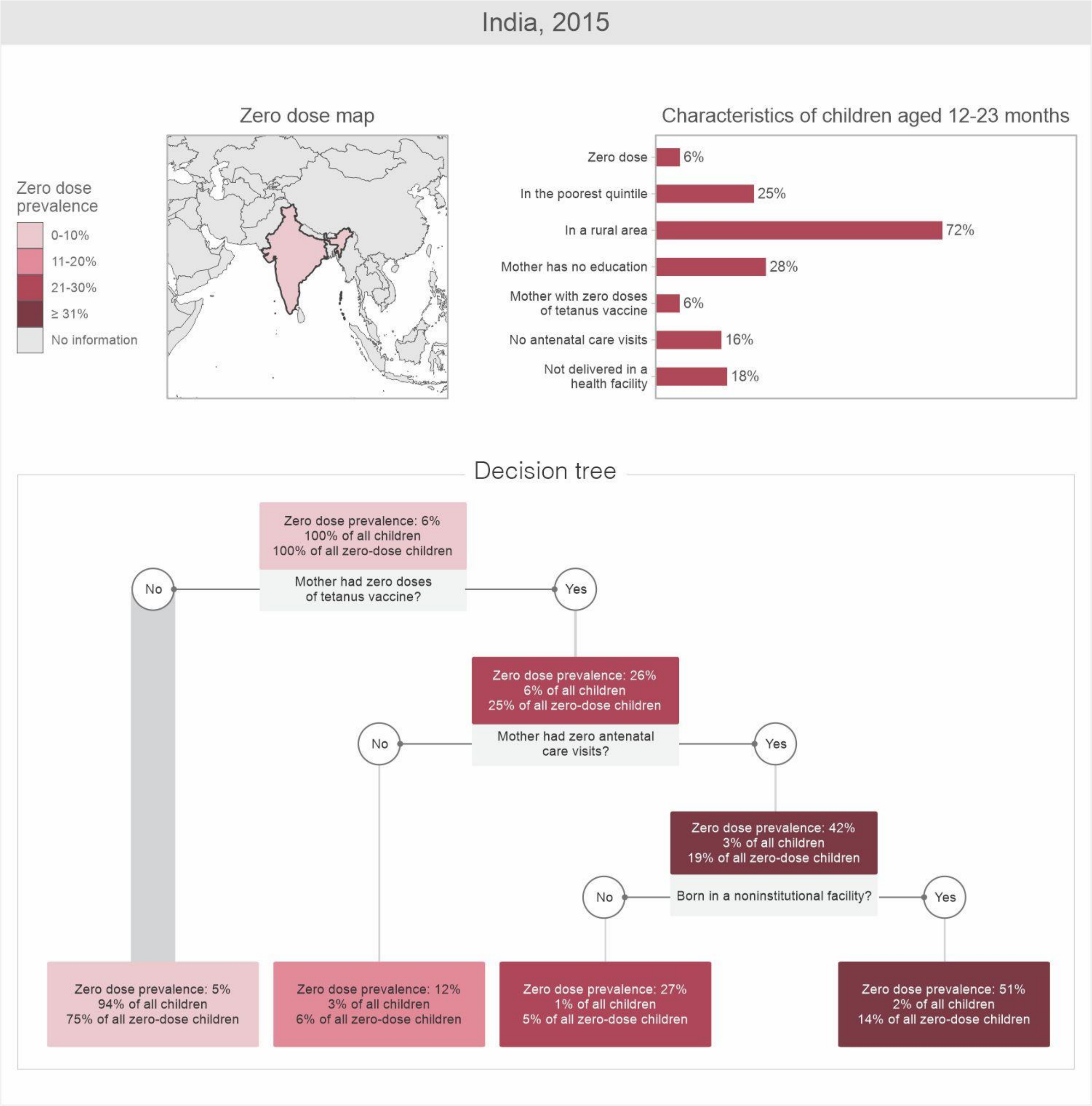

Figure S36. – India's country profile.

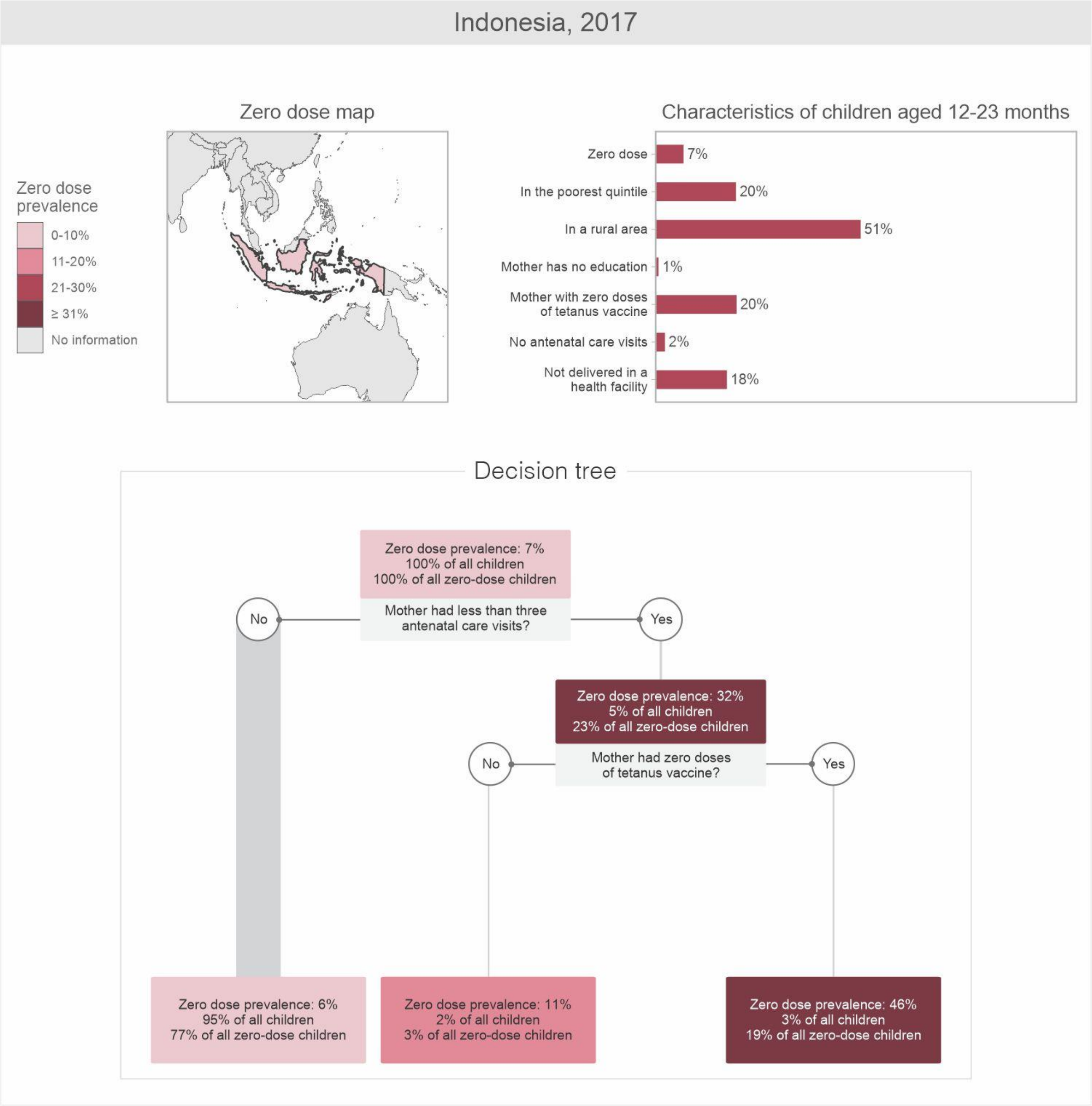

Figure S37. – Indonesia's country profile.

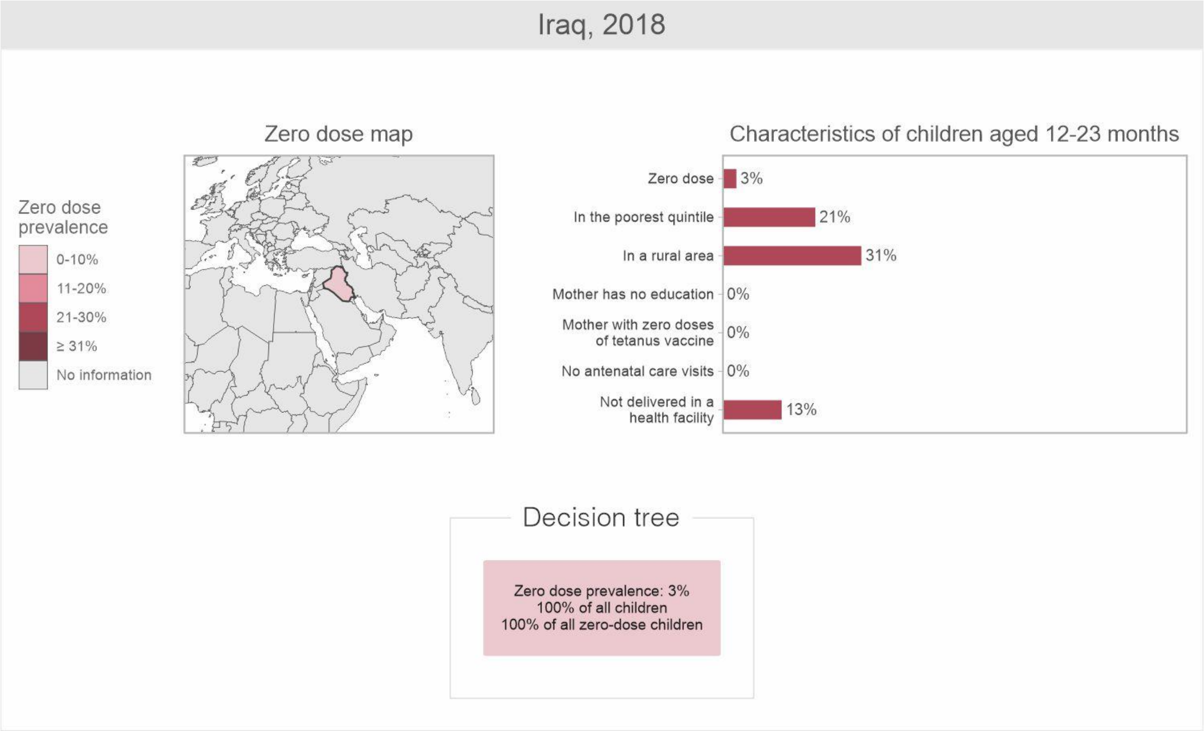

Figure S38. – Iraq's country profile.

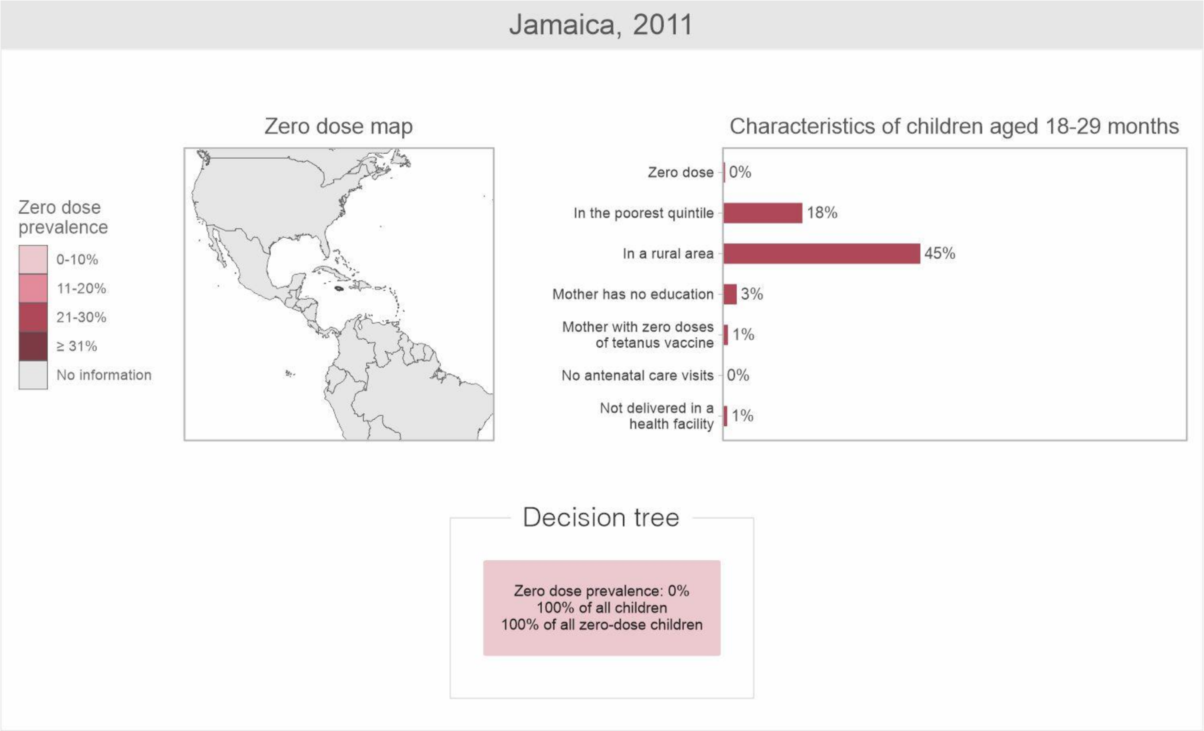

Figure S39. – Jamaica's country profile.

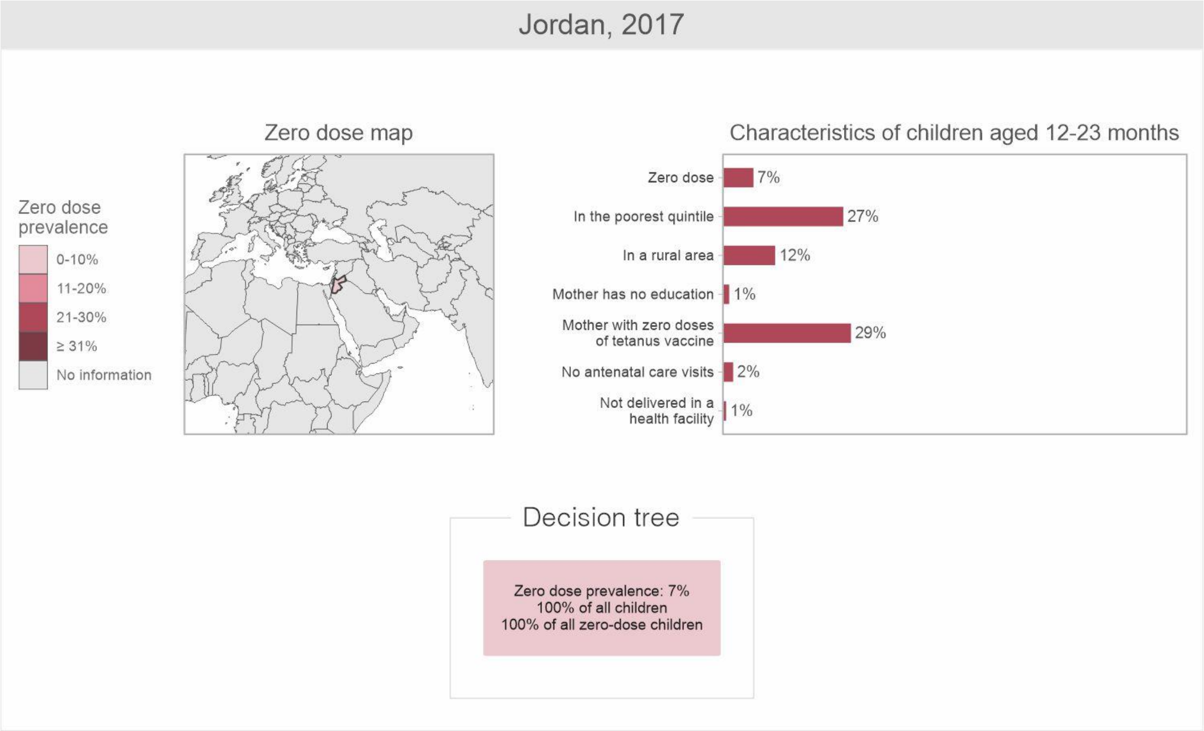

Figure S40. – Jordan's country profile.

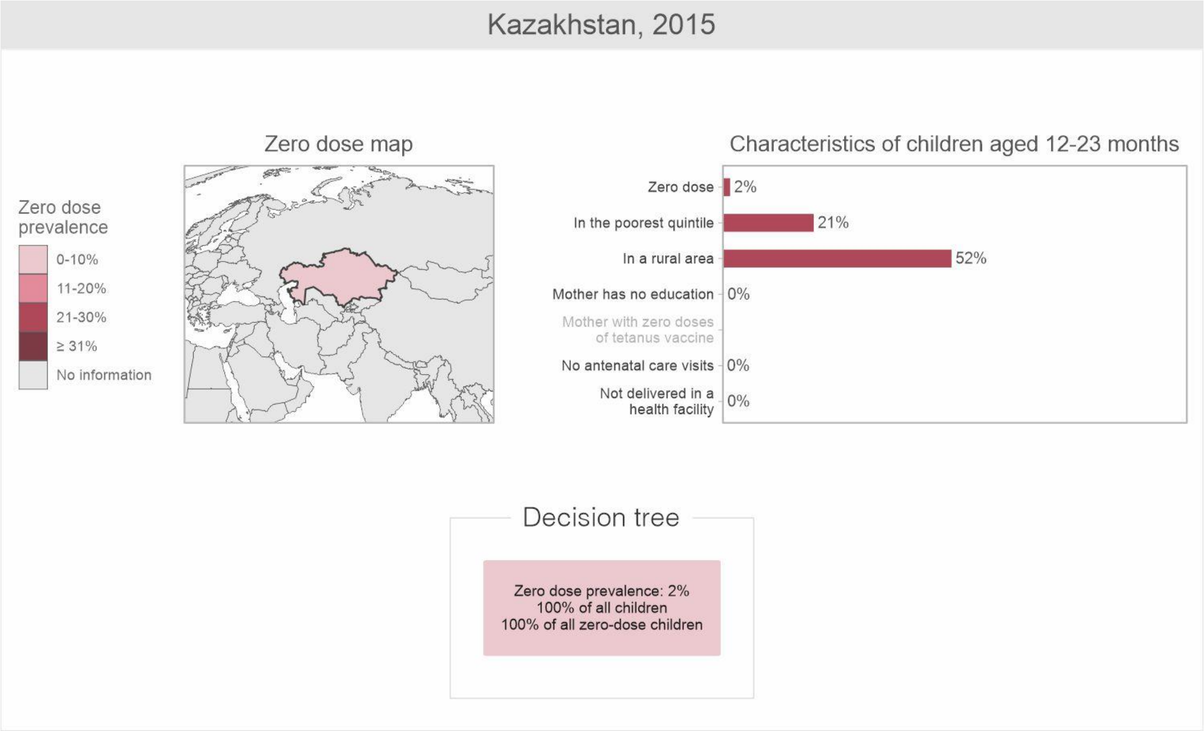

Figure S41. – Kazakhstan's country profile.

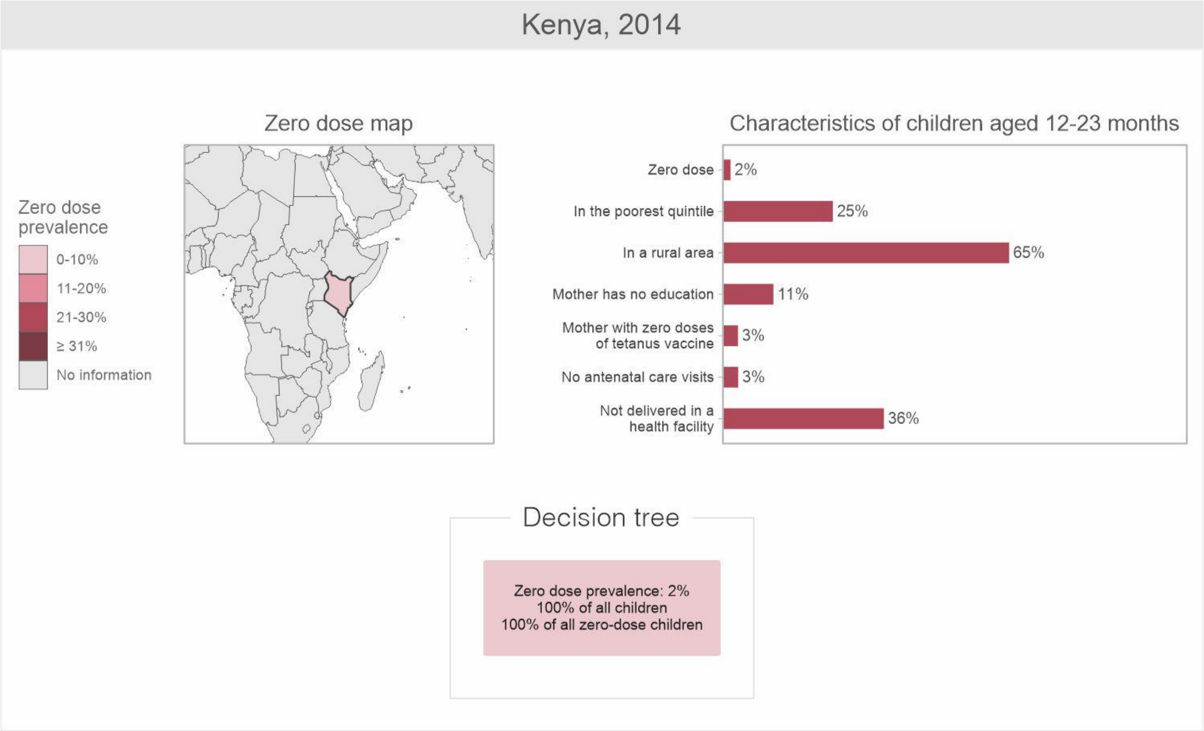

Figure S42. – Kenya's country profile.

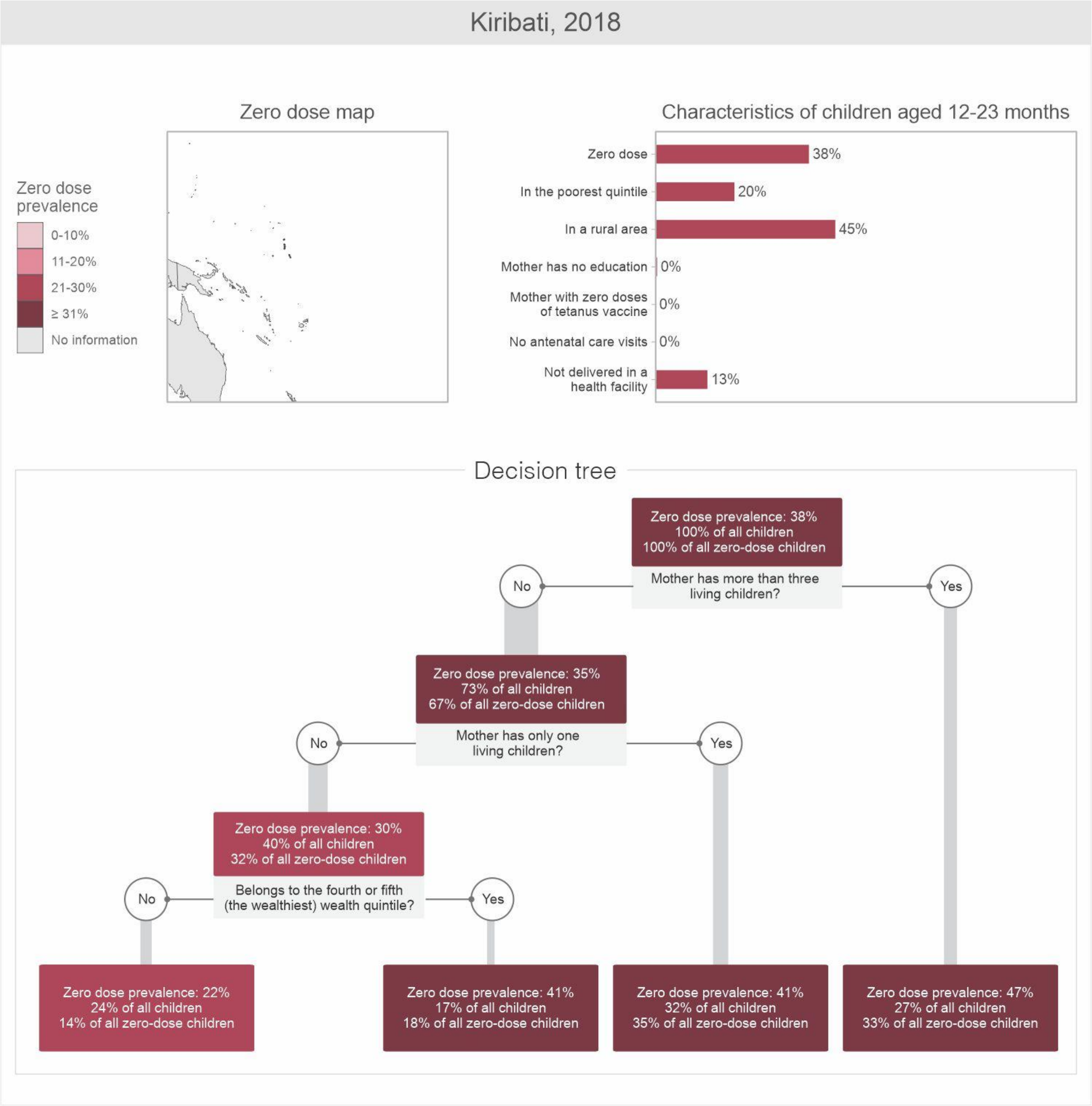

Figure S43. – Kiribati's country profile.

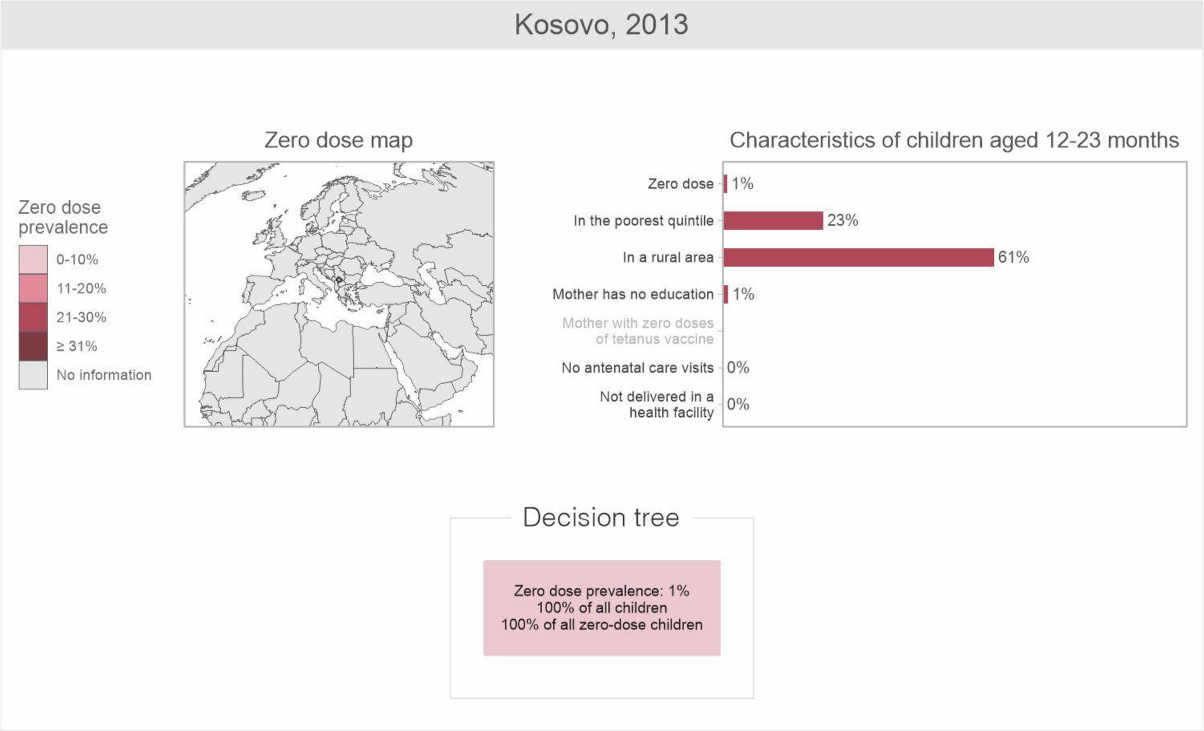

Figure S44. – Kosovo's country profile.

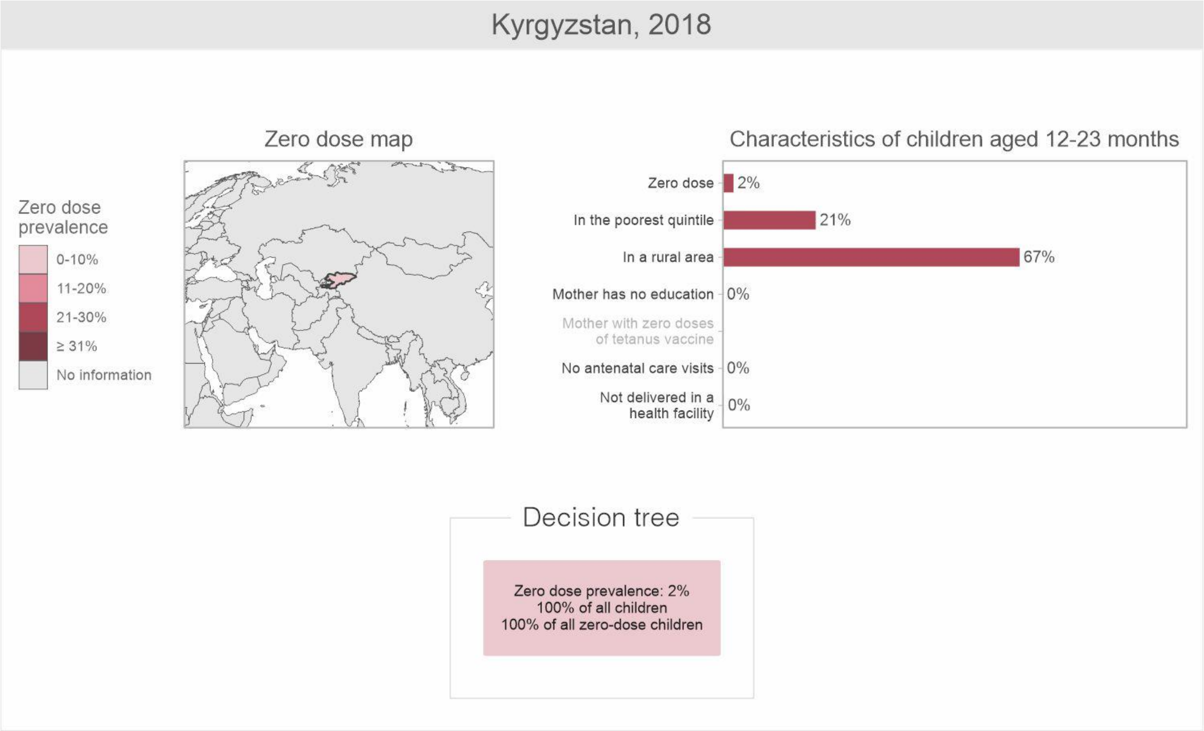

Figure S45. – Kyrgystan's country profile.

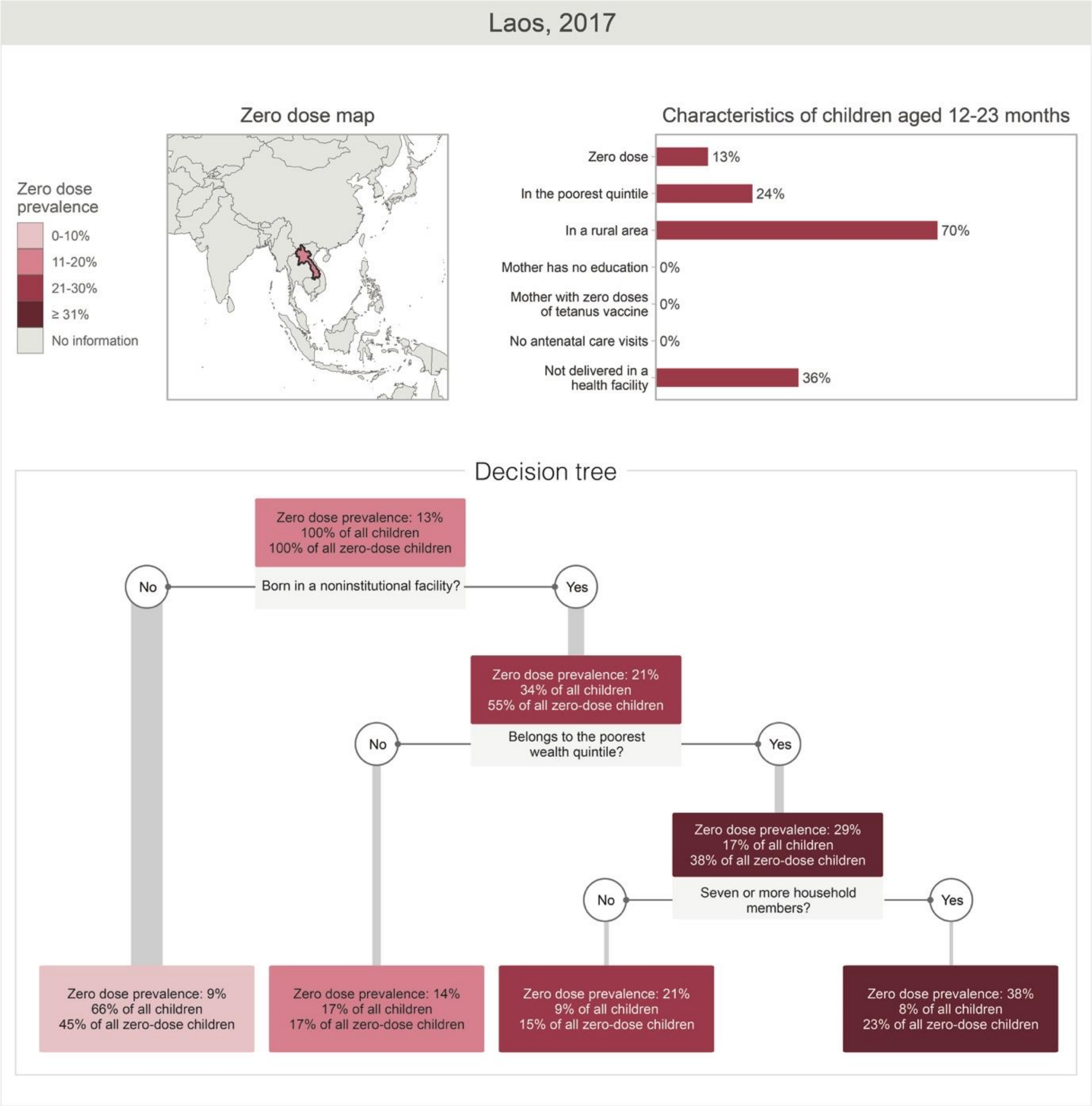

Figure S46. – Laos' country profile.

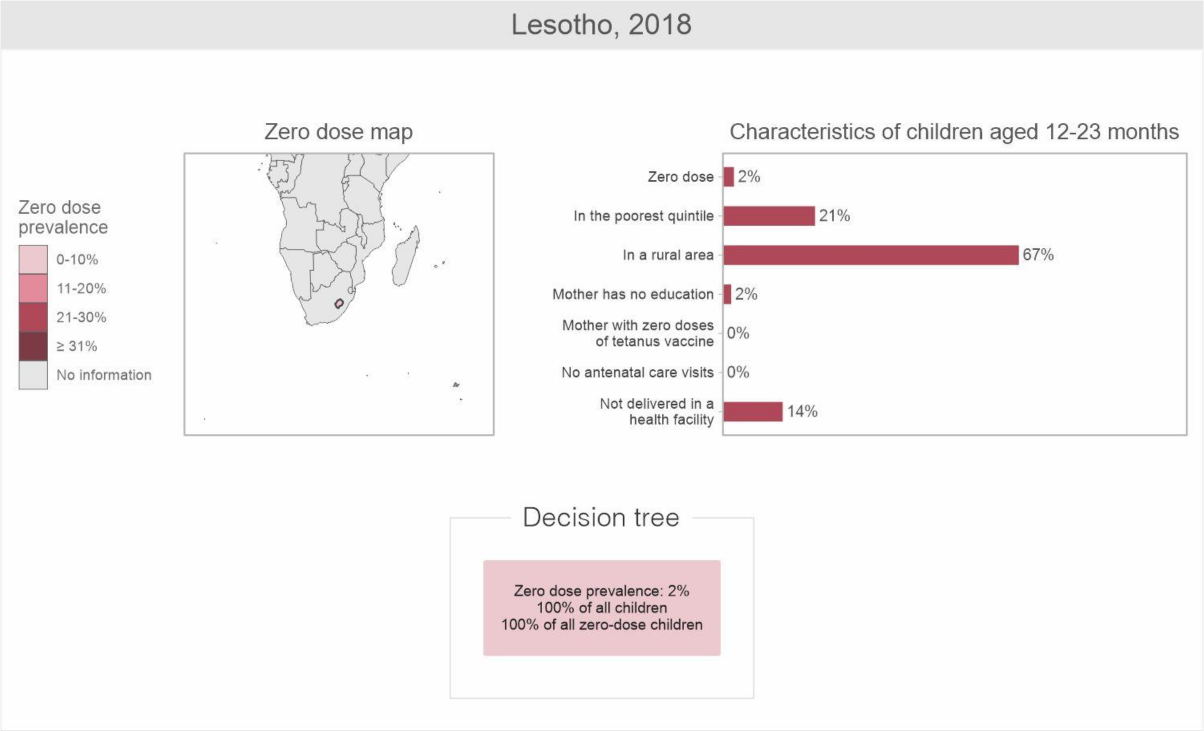

Figure S47. – Lesotho's country profile.

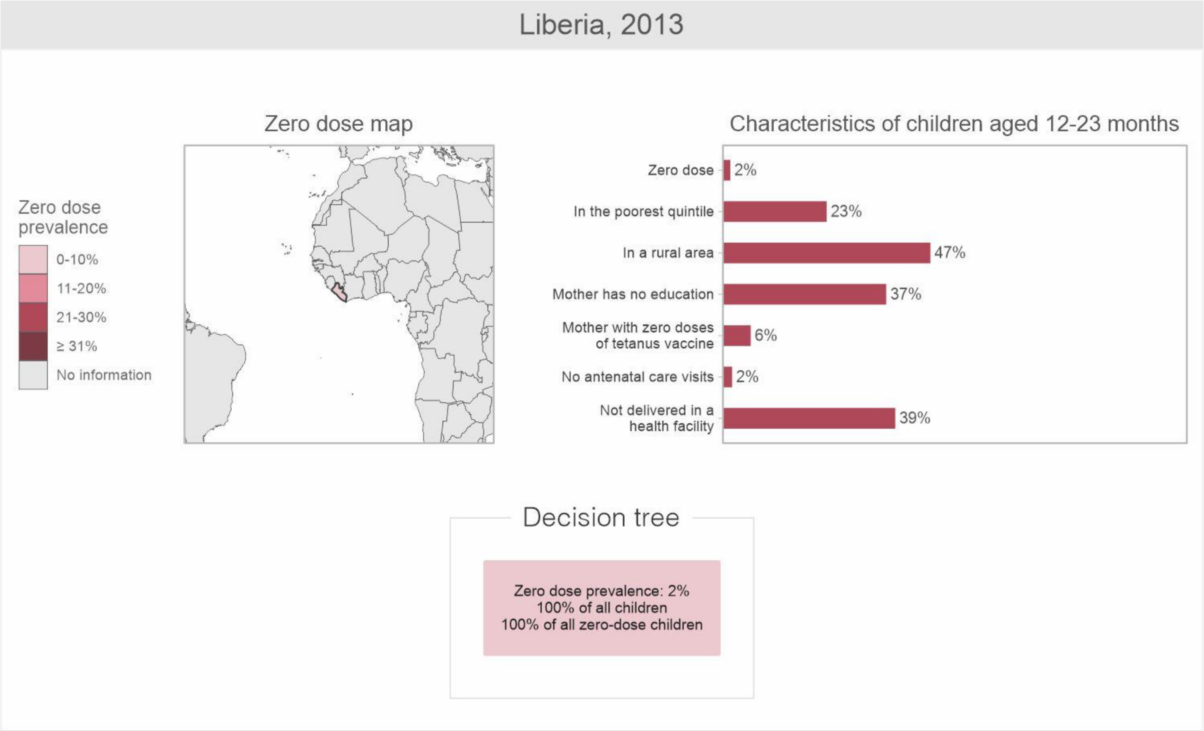

Figure S48. – Liberia's country profile.

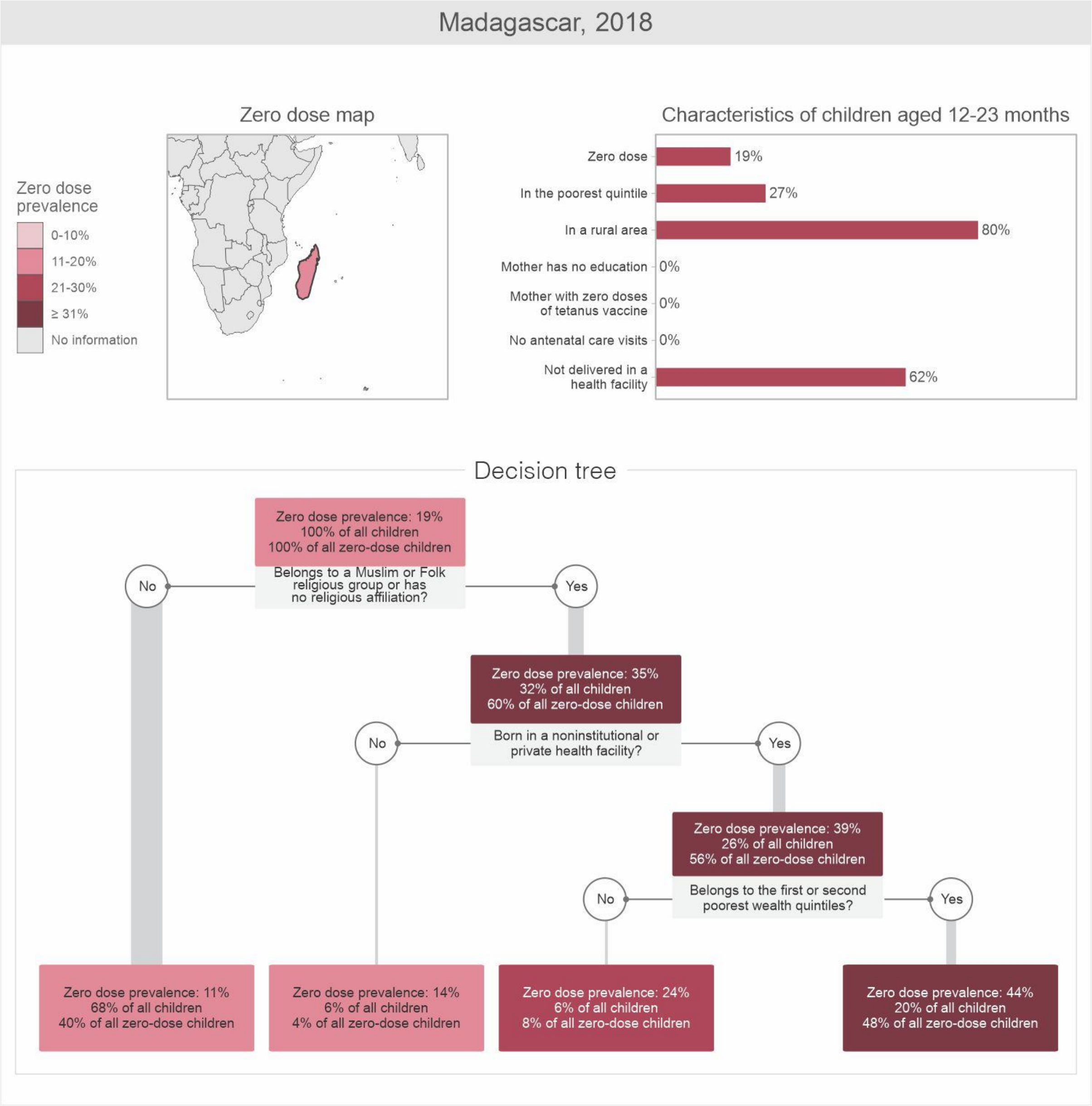

Figure S49. – Madagascar's country profile.

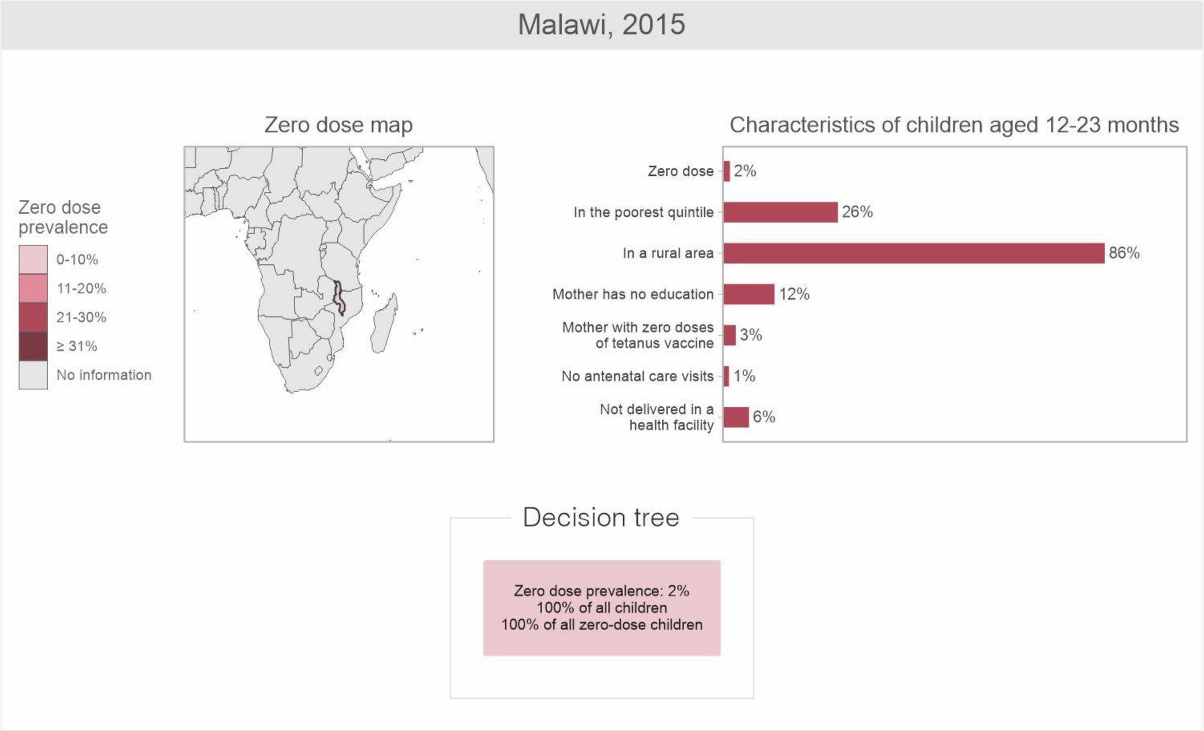

Figure S50. – Malawi's country profile.

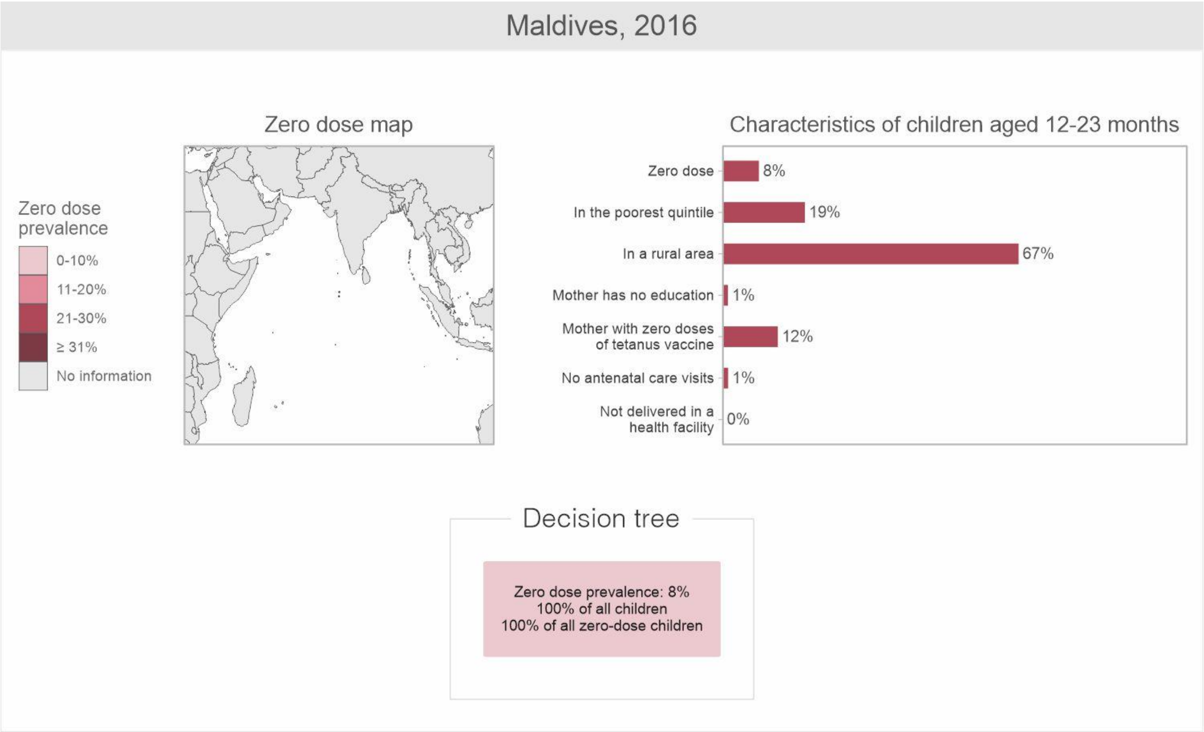

Figure S51. – Maldives' country profile.

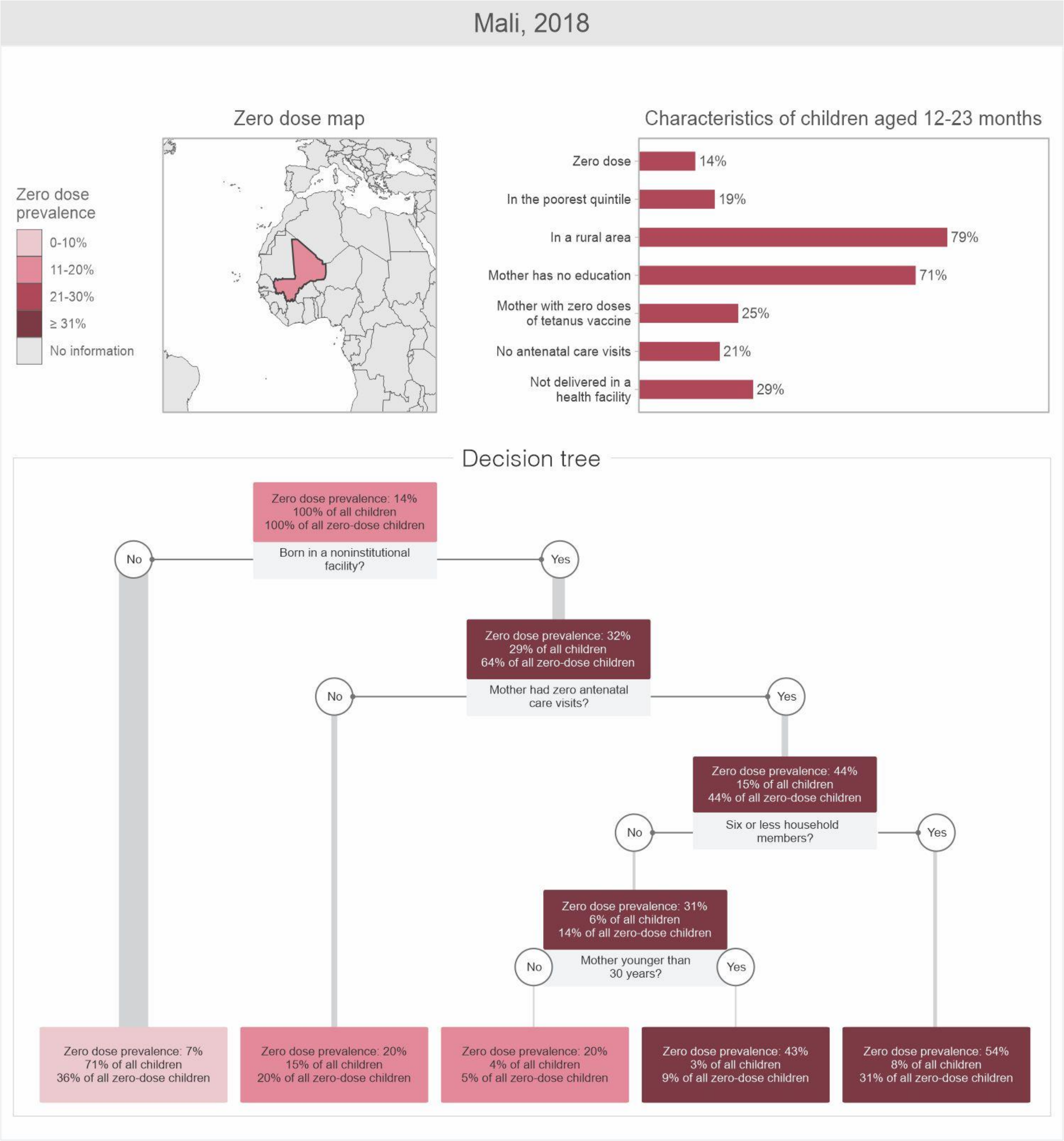

Figure S52. – Mali's country profile.

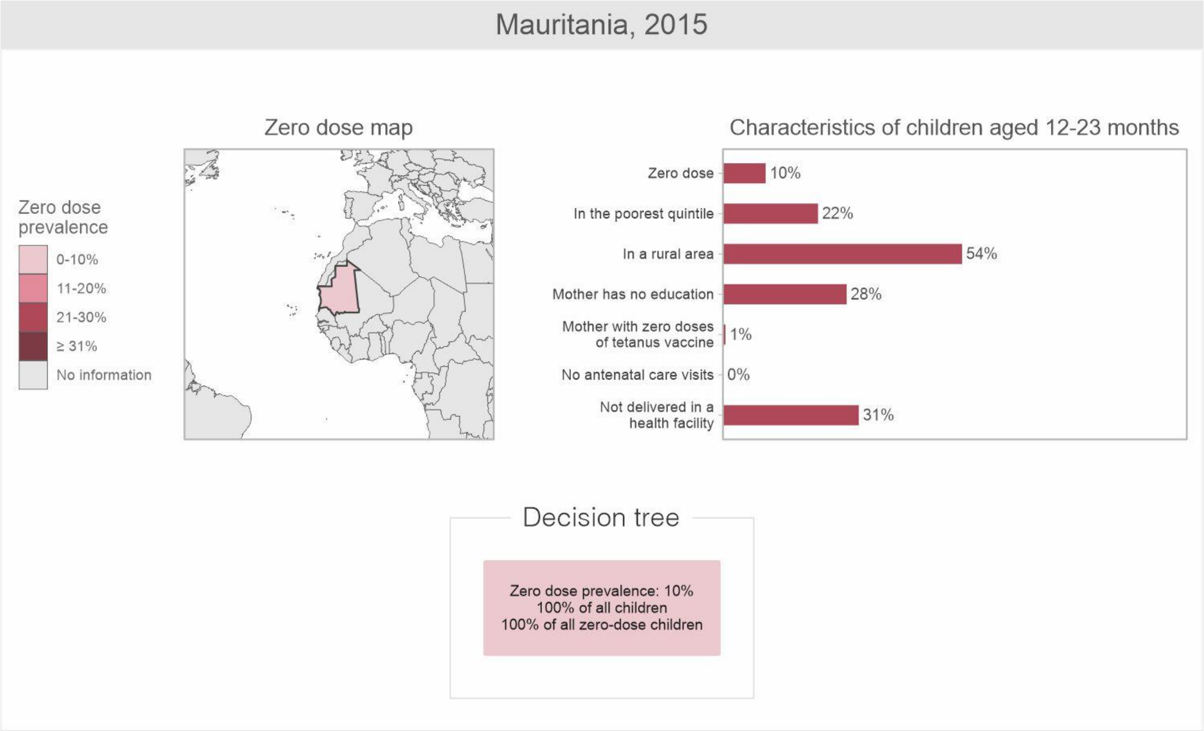

Figure S53. – Mauritania's country profile.

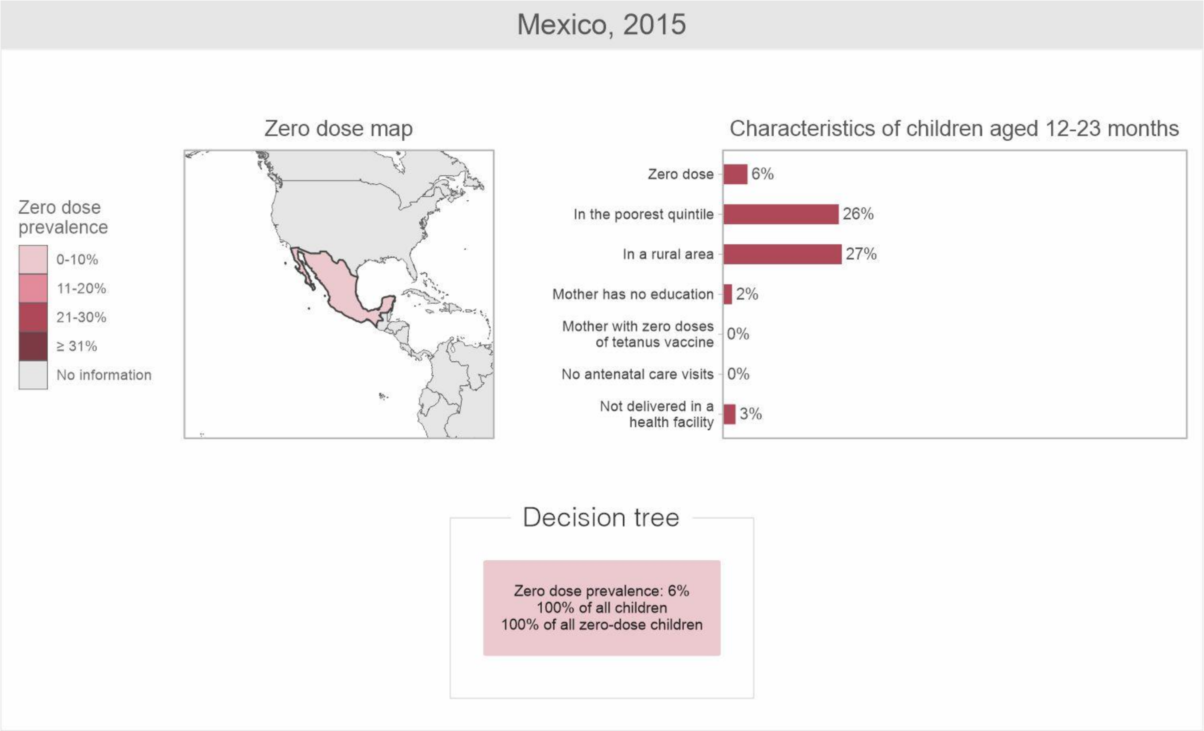

Figure S54. – Mexico's country profile.

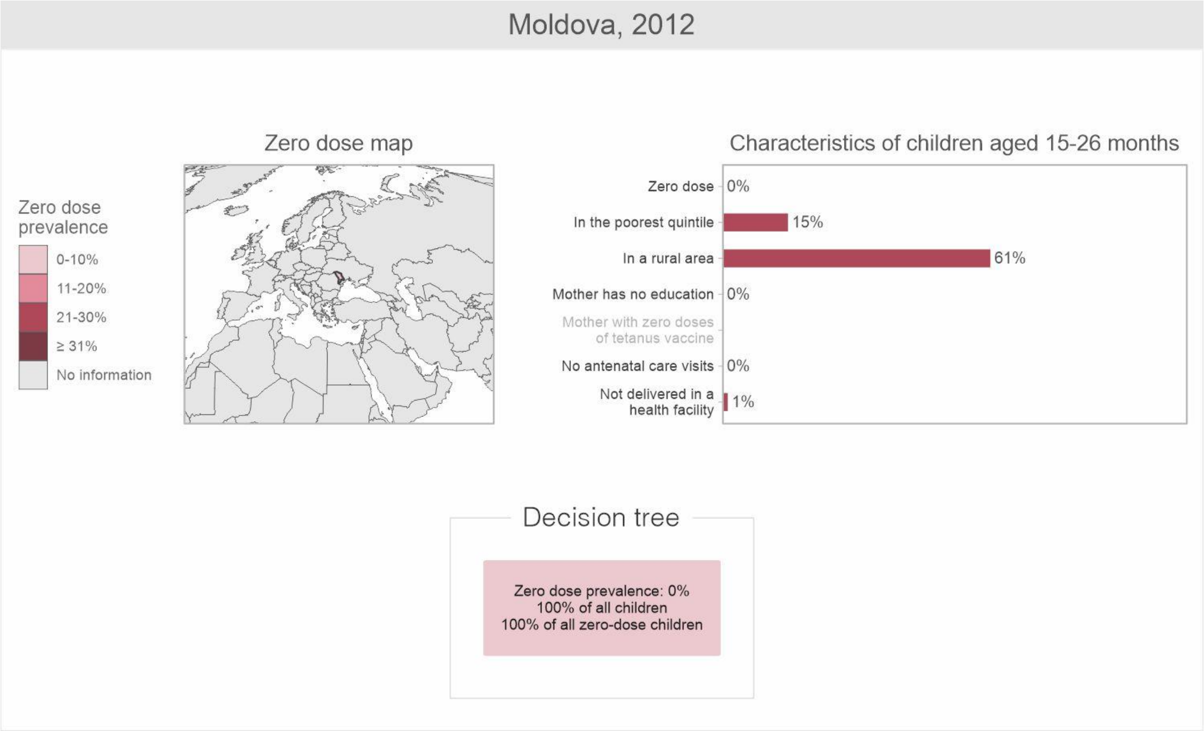

Figure S55. – Moldova's country profile.

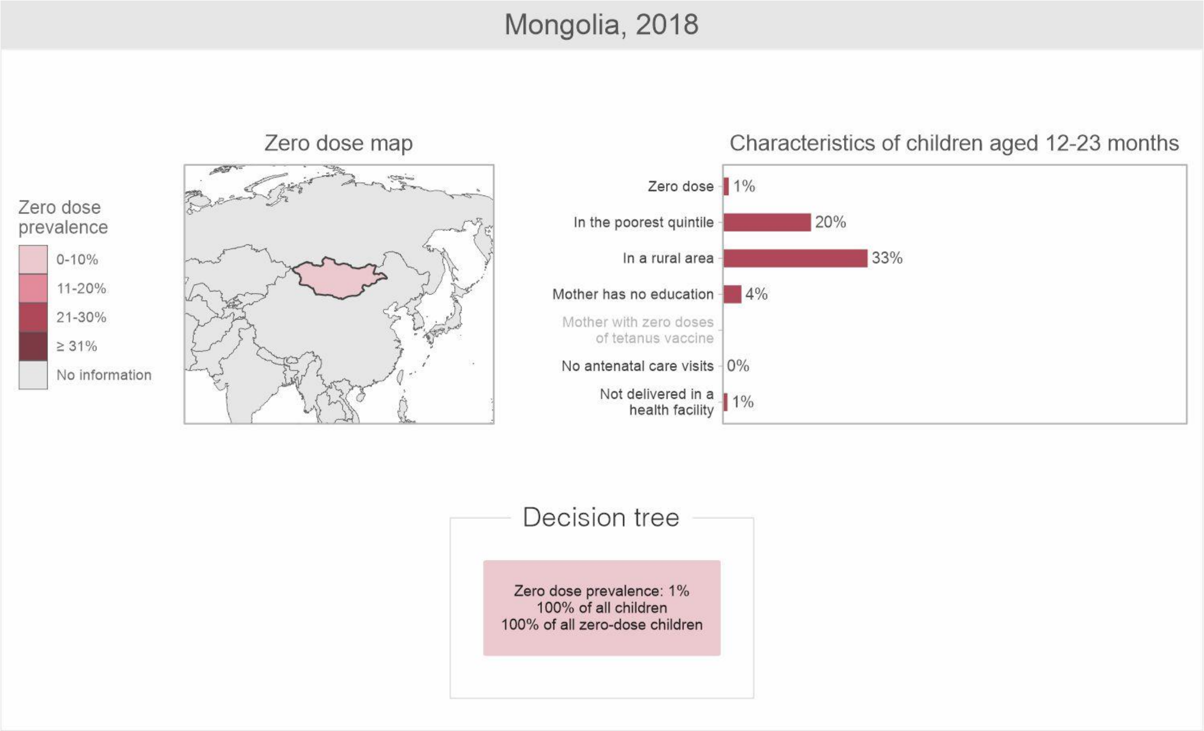

Figure S56. – Mongolia's country profile.

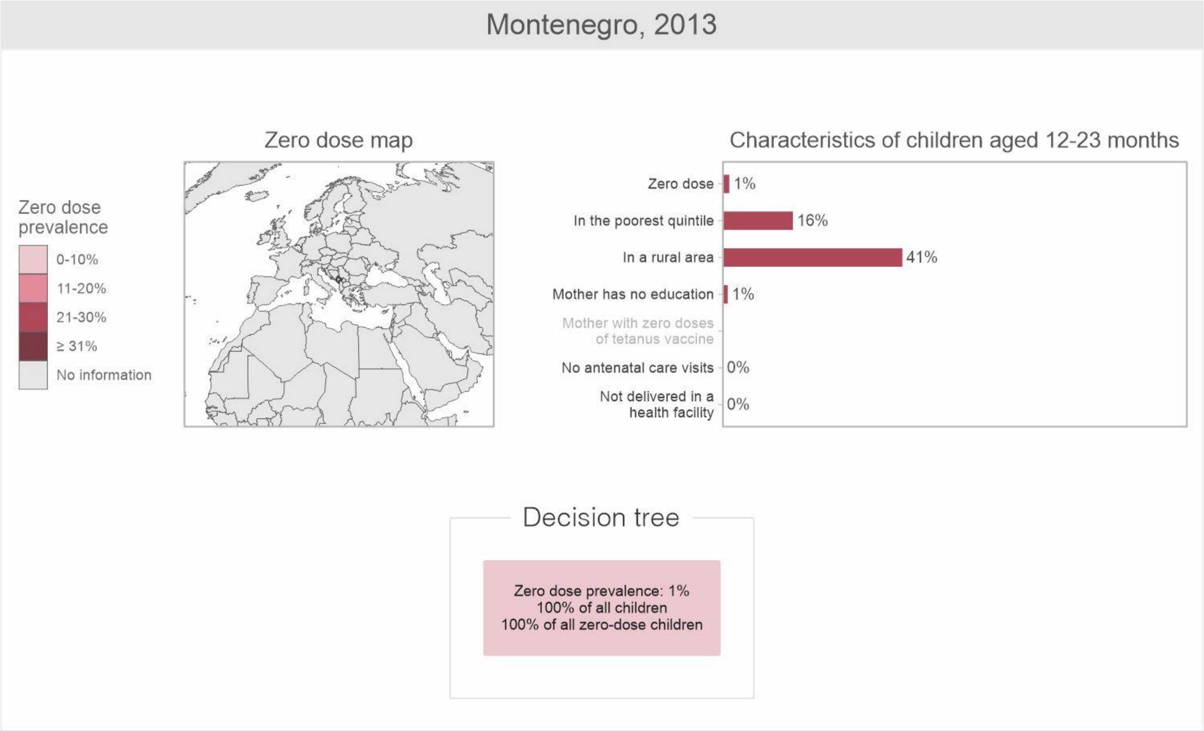

Figure S57. – Montenegro's country profile.

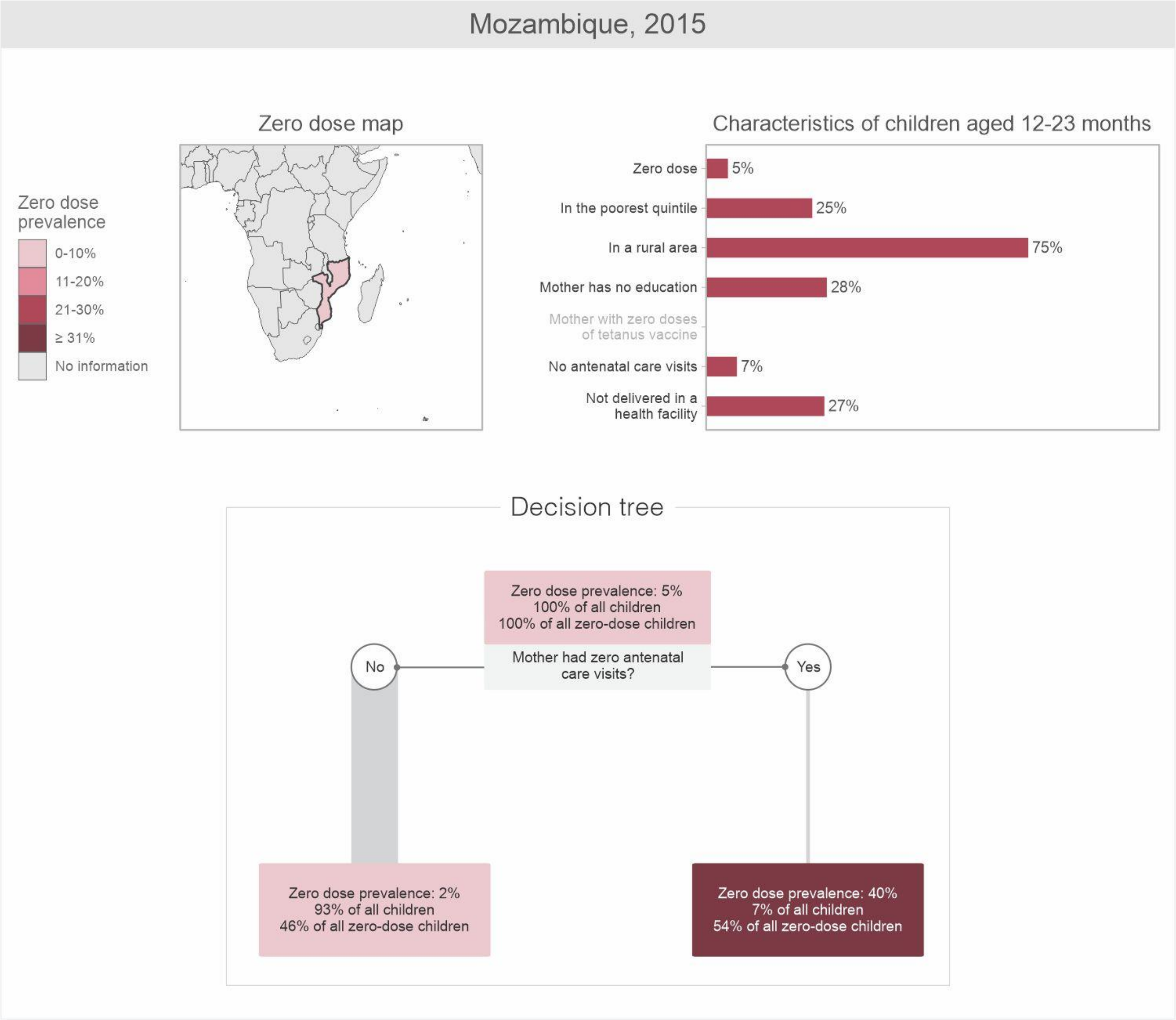

Figure S58. – Mozambique's country profile.

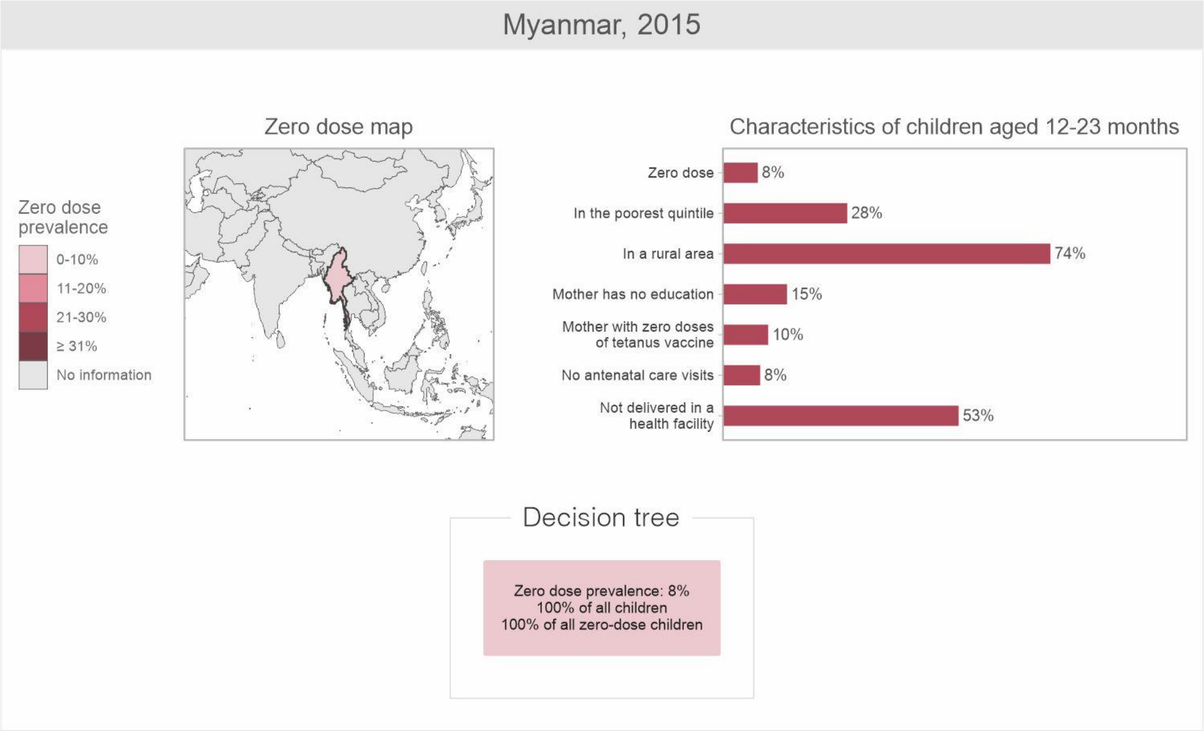

Figure S59. – Myanmar's country profile.

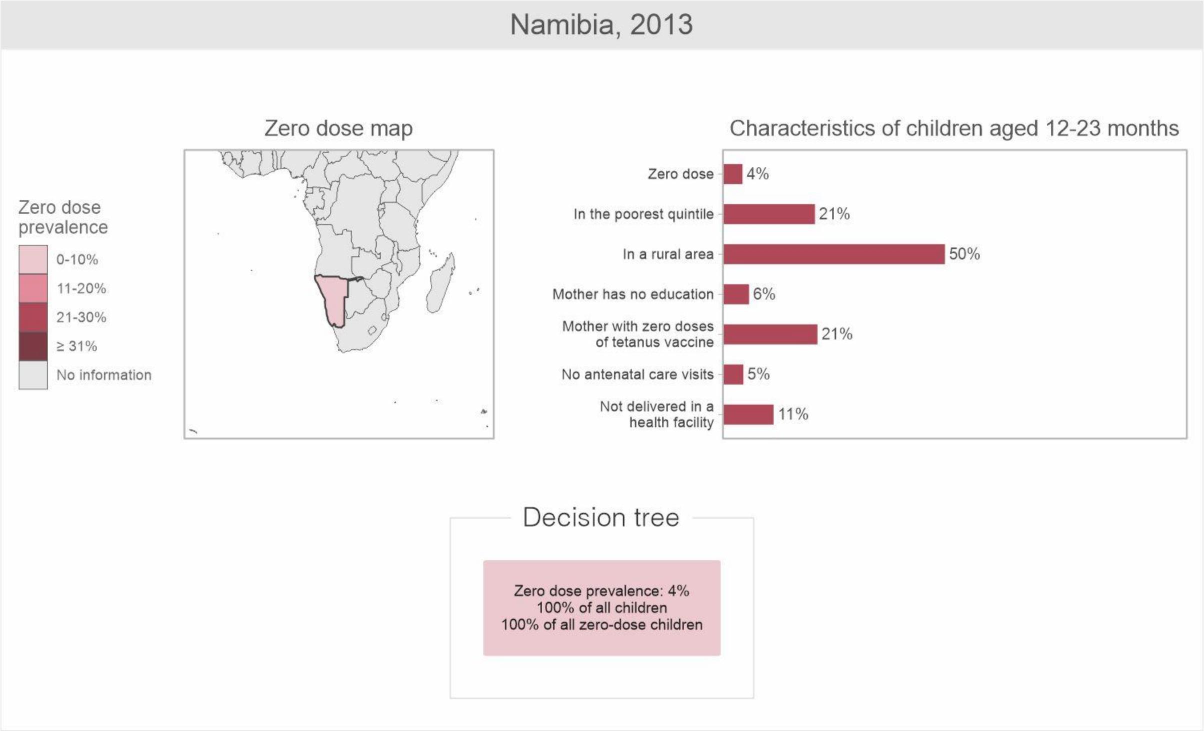

Figure S60. – Namibia's country profile.

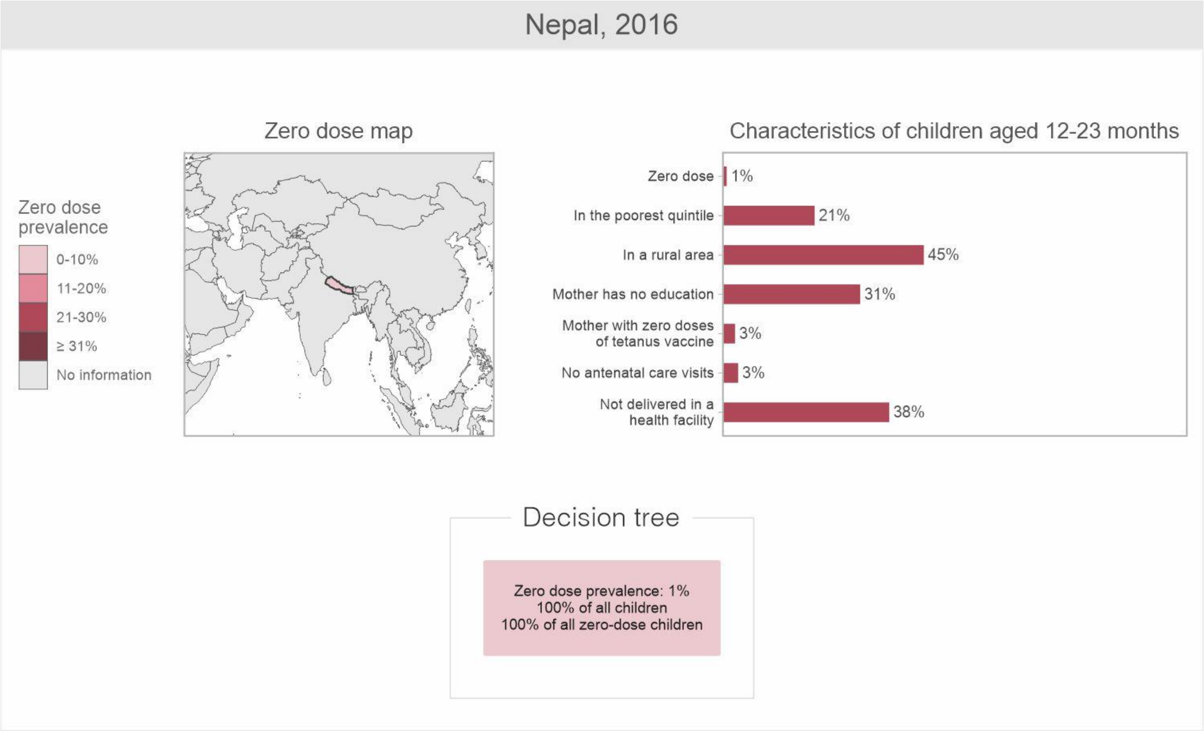

Figure S61. – Nepal's country profile.

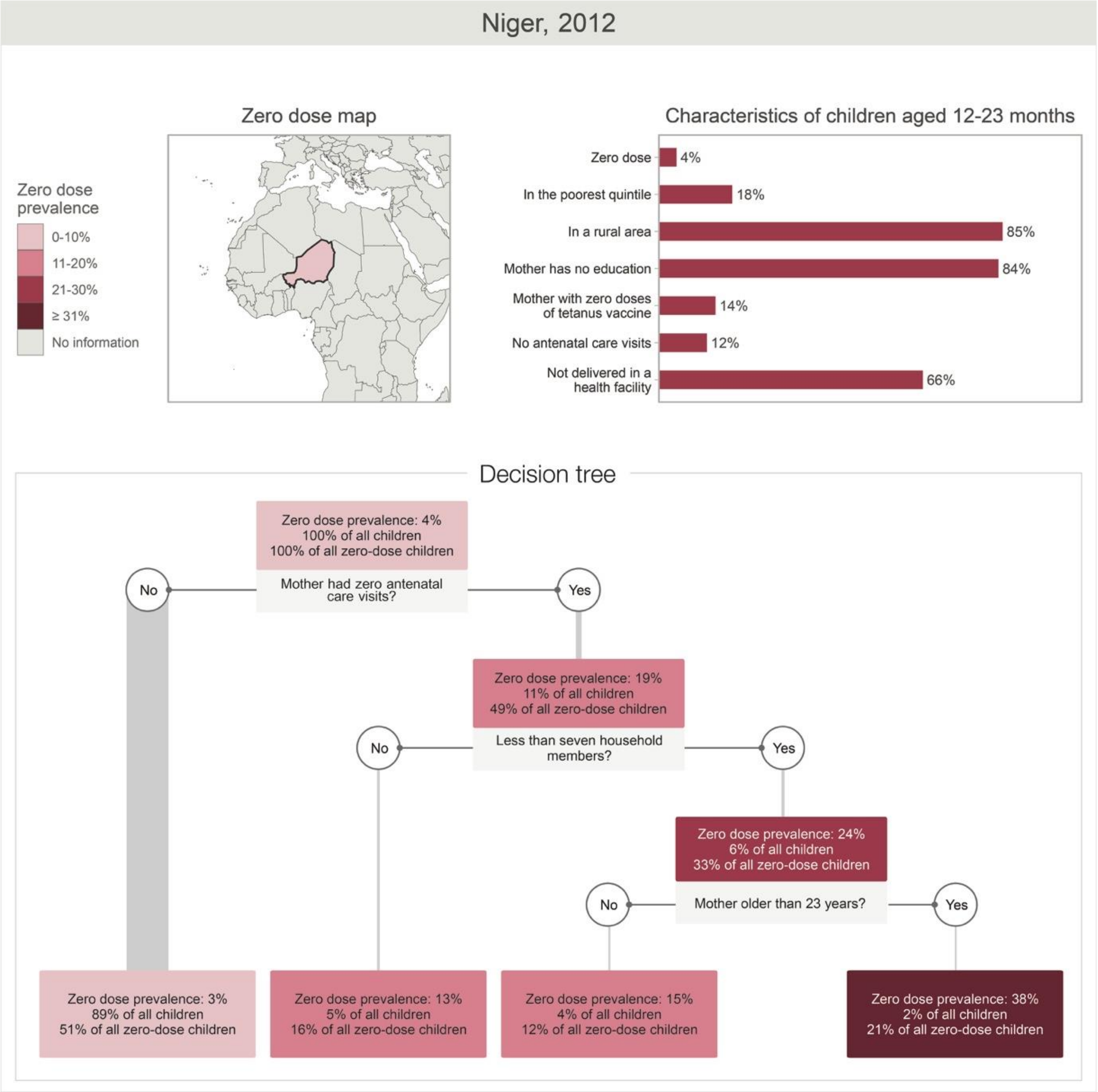

Figure S62. – Niger's country profile.

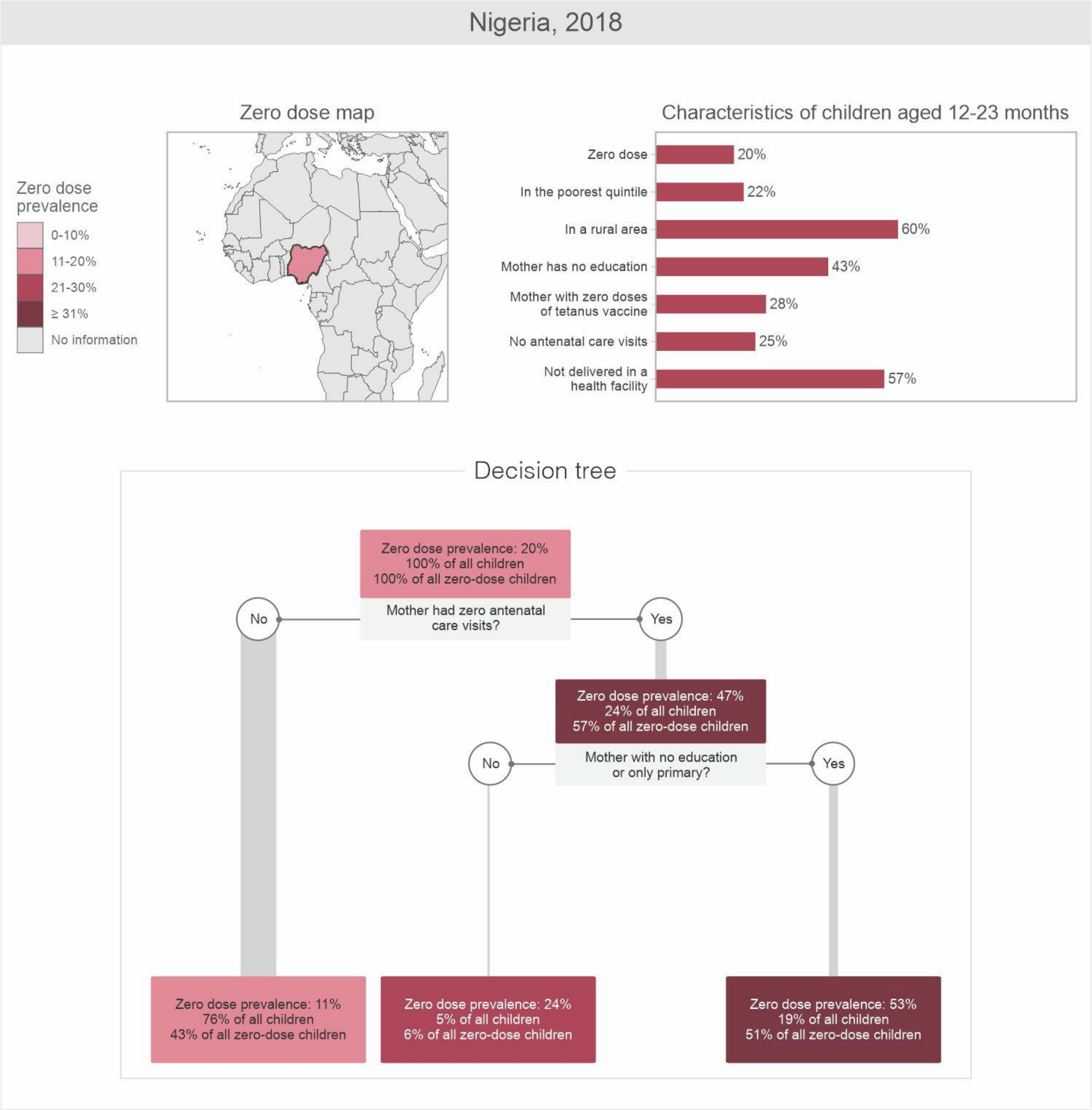

Figure S63. – Nigeria's country profile.

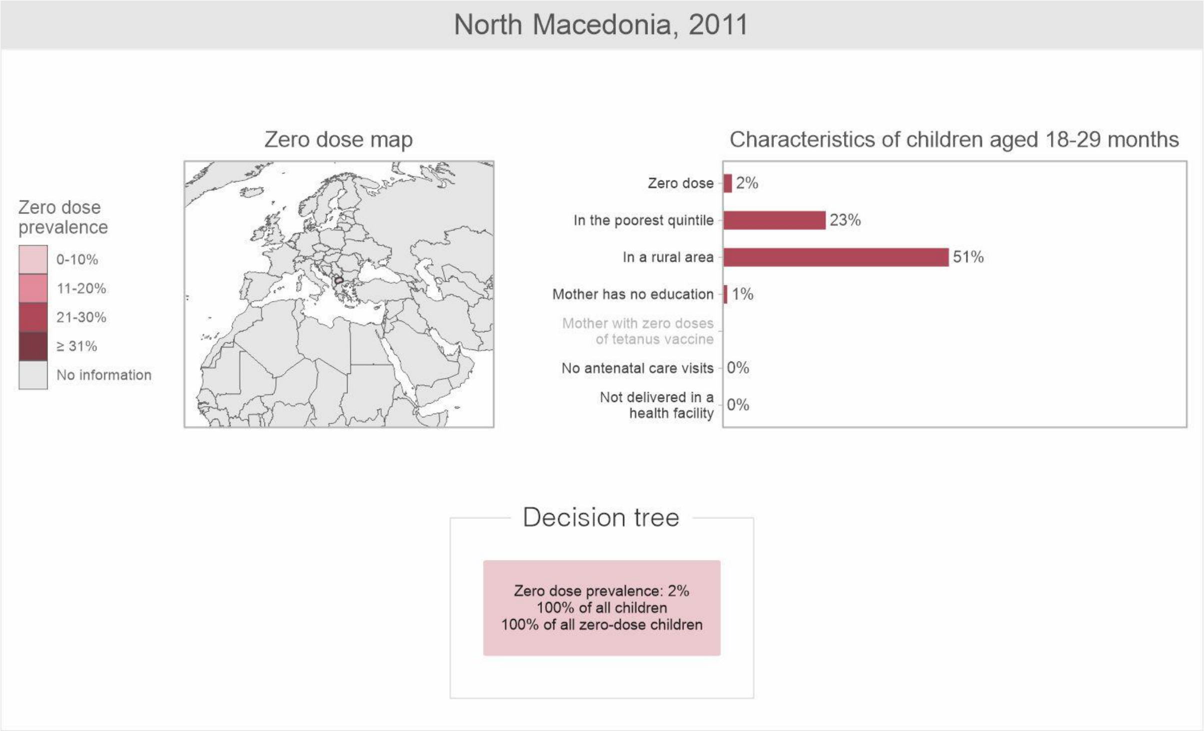

Figure S64. – North Macedonia's country profile.

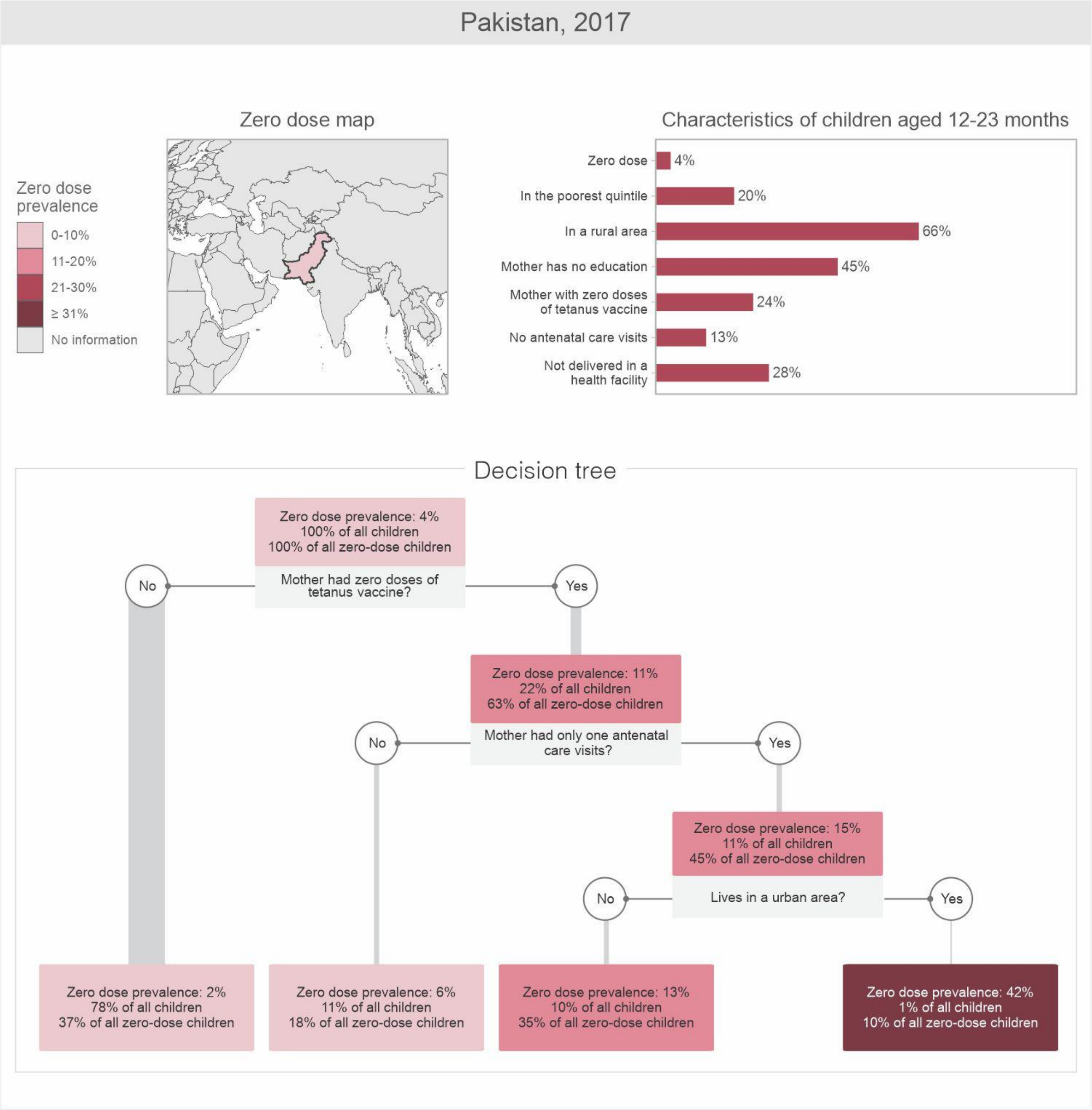

Figure S65. – Pakistan's country profile.

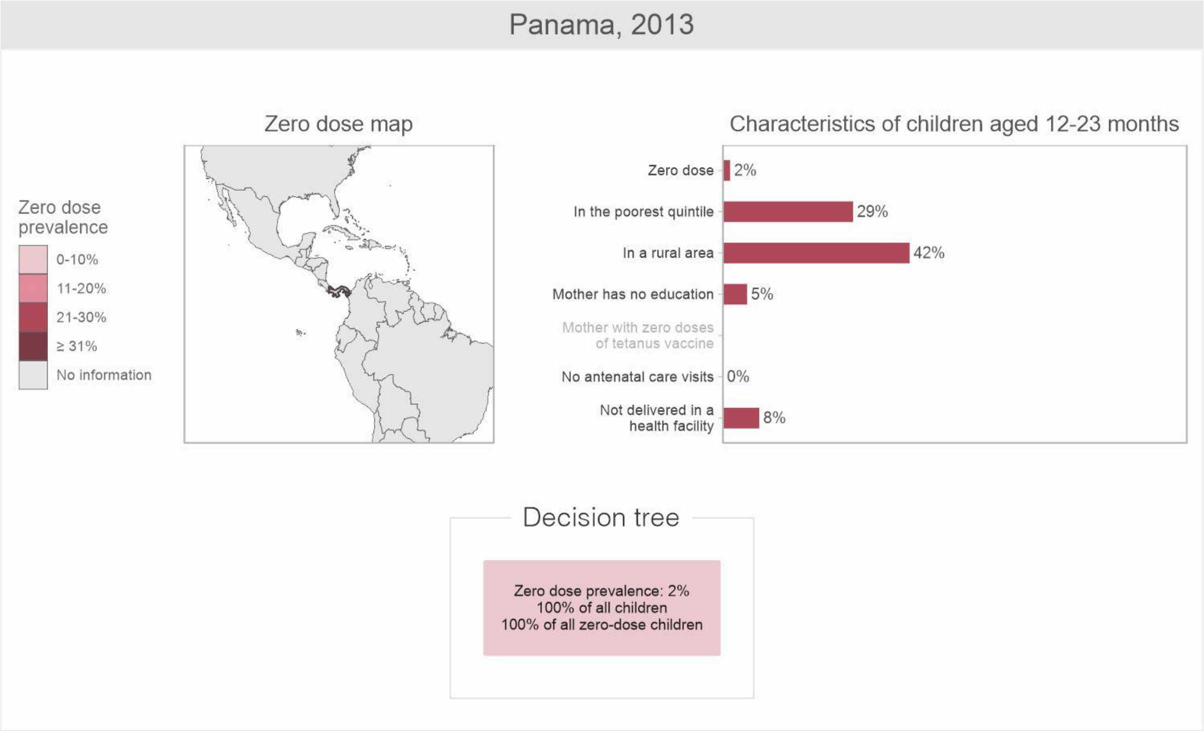

Figure S66. – Panama's country profile.

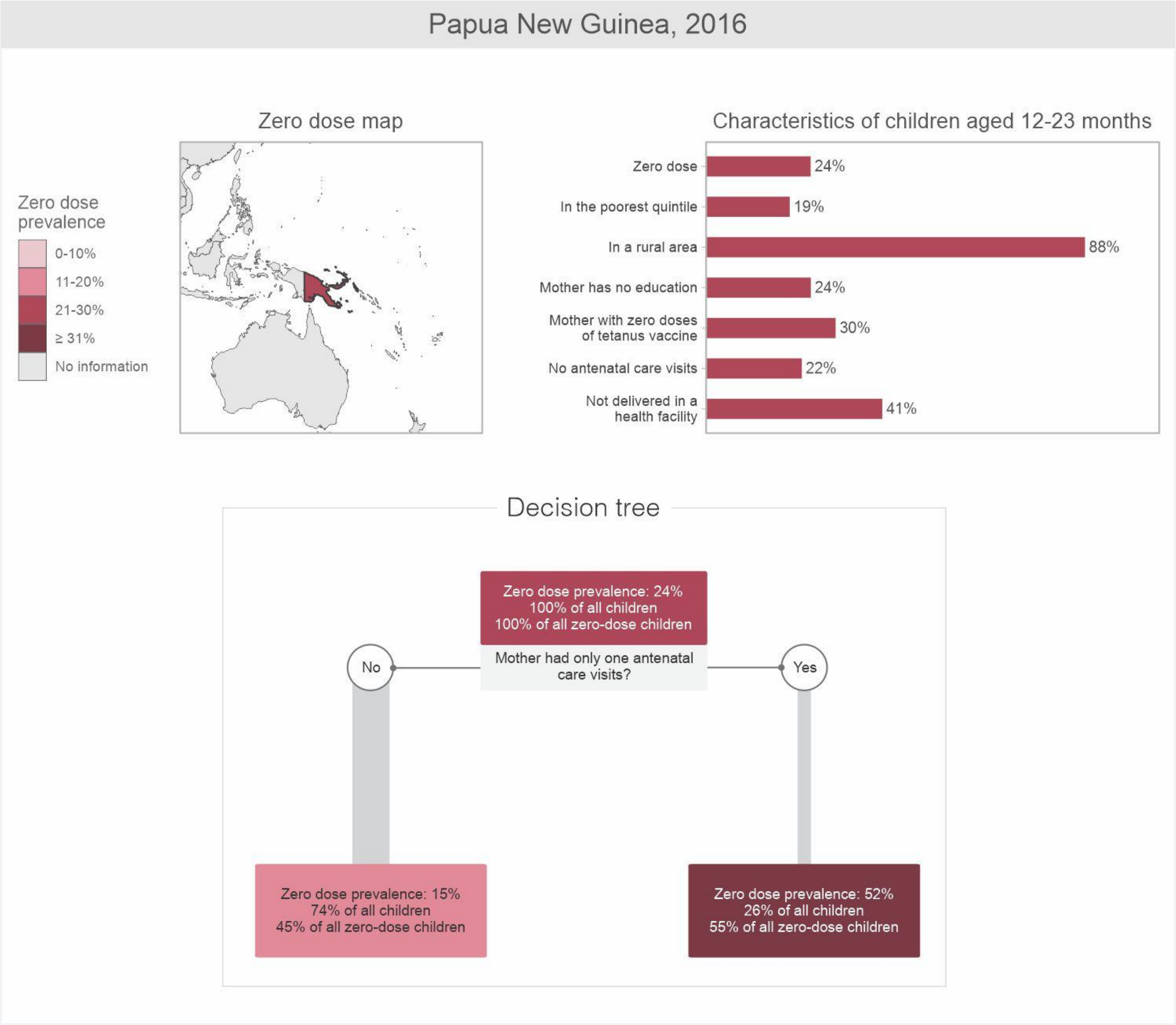

Figure S67. – Papua New Guinea's country profile.

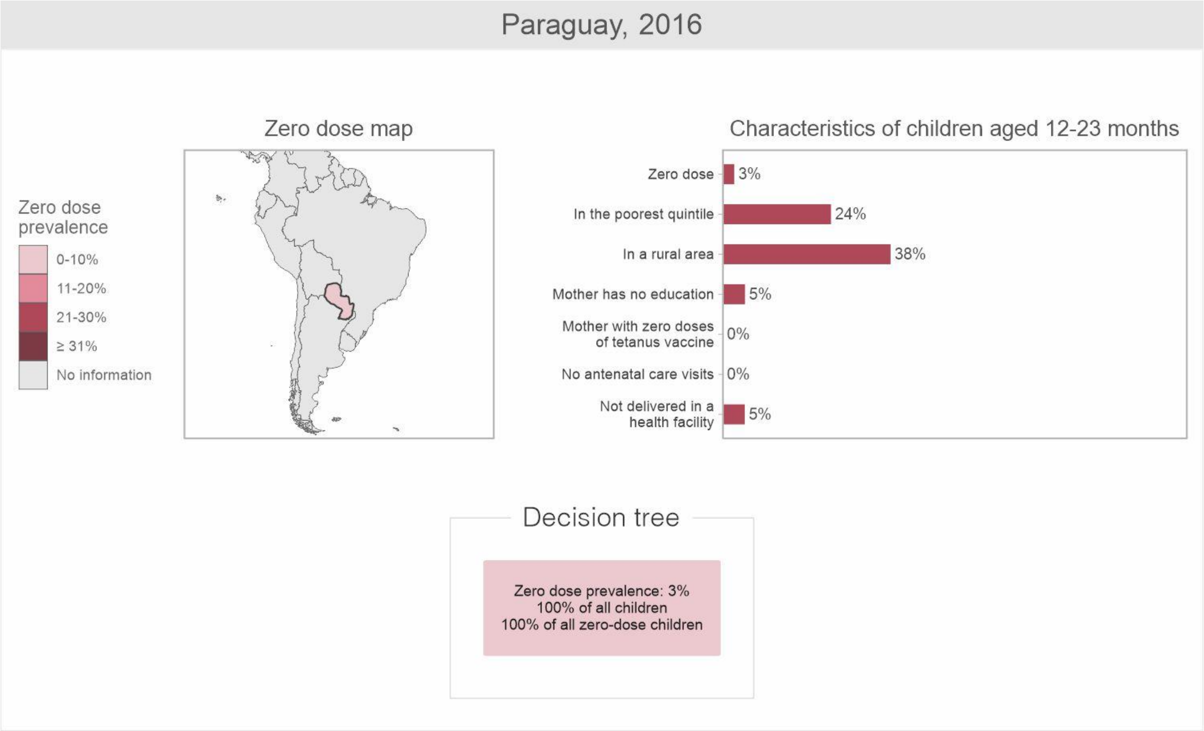

Figure S68. – Paraguay's country profile.

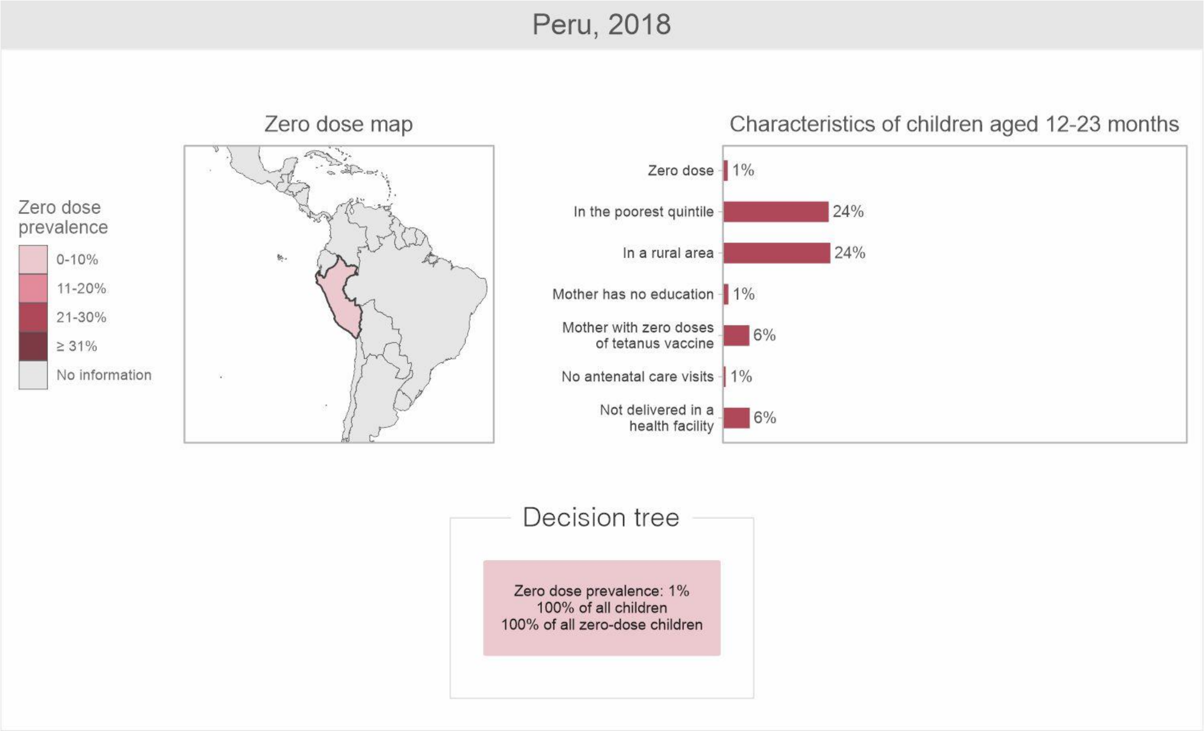

Figure S69. – Peru's country profile.

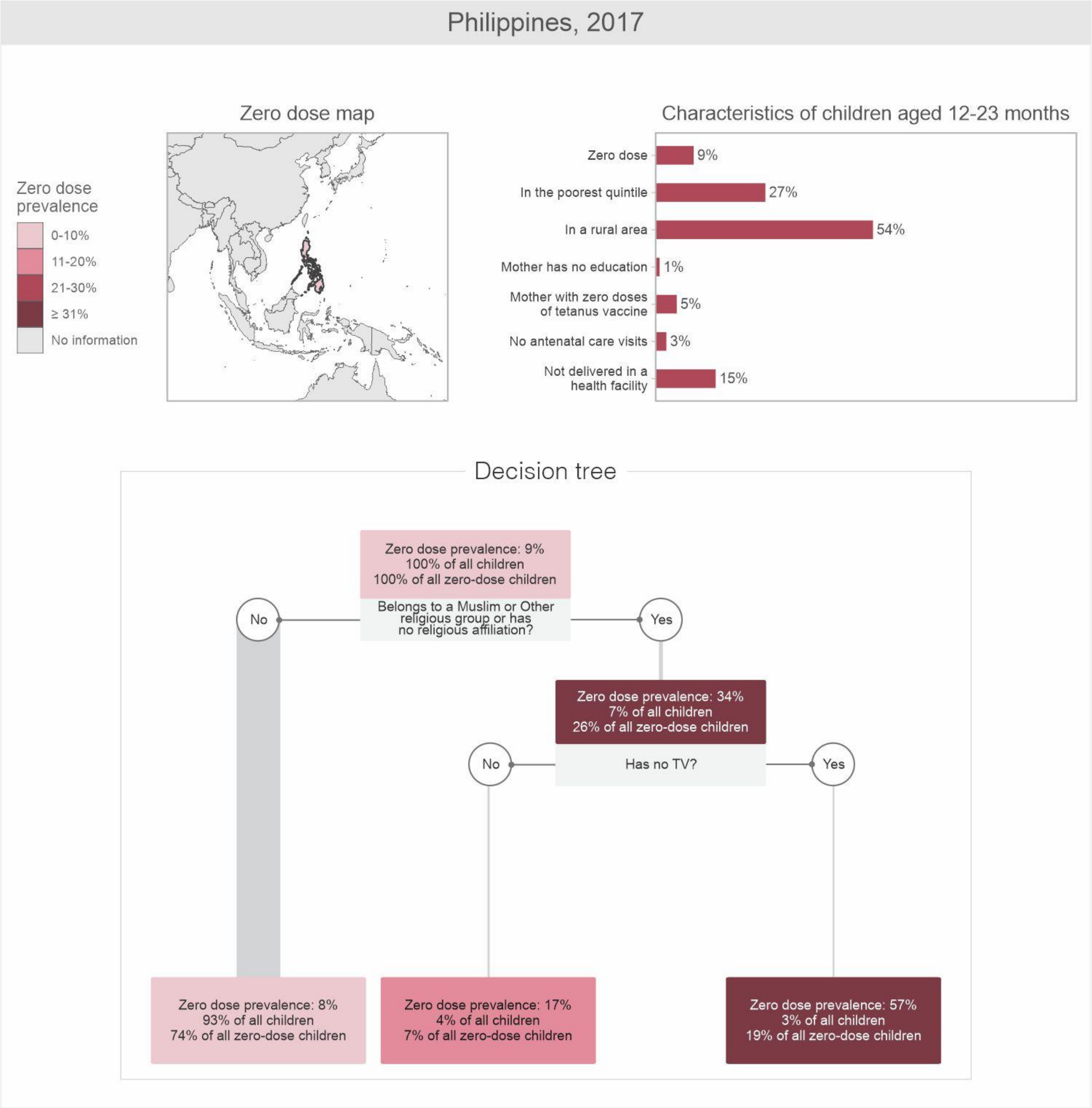

Figure S70. – Philippines' country profile.

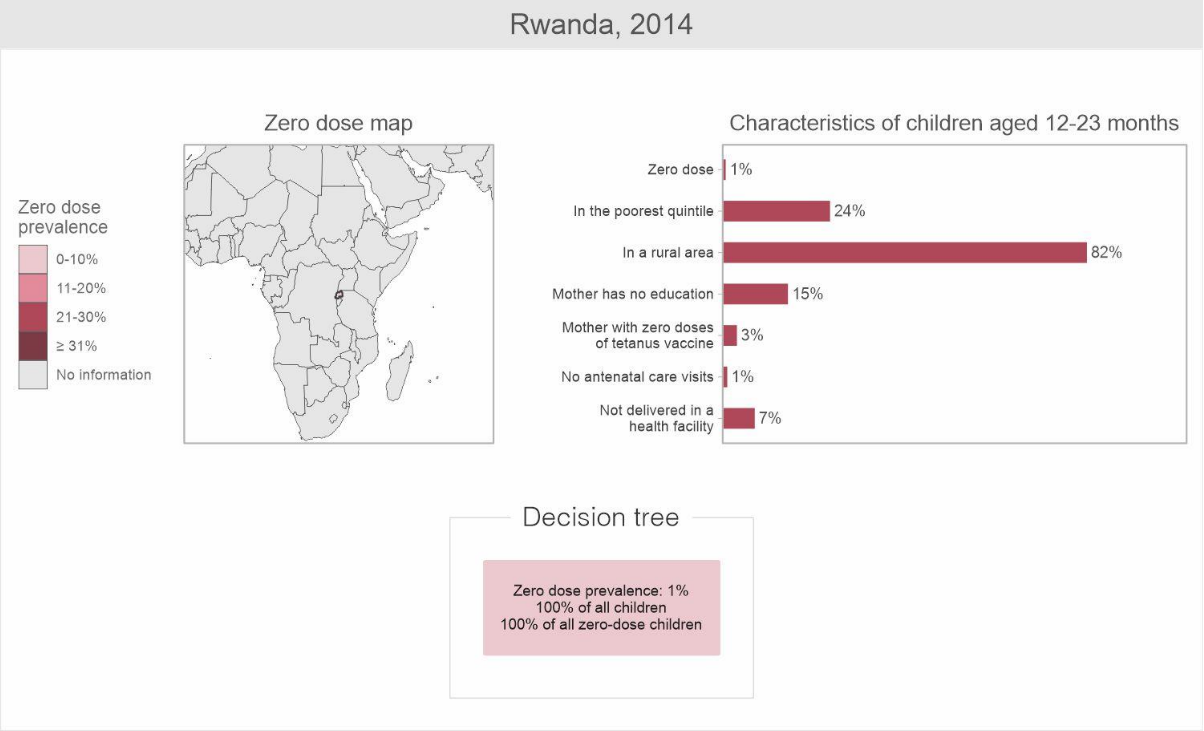

Figure S71. – Rwanda's country profile.

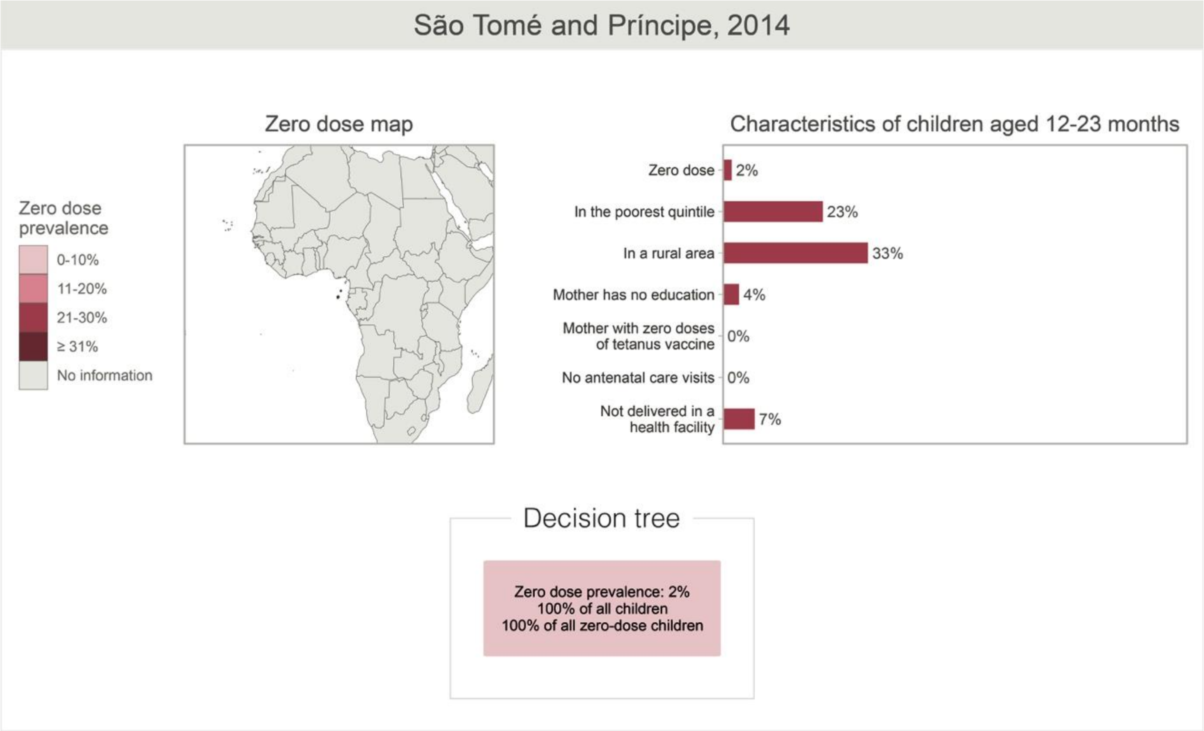

Figure S72. – São Tomé and Príncipe's country profile.

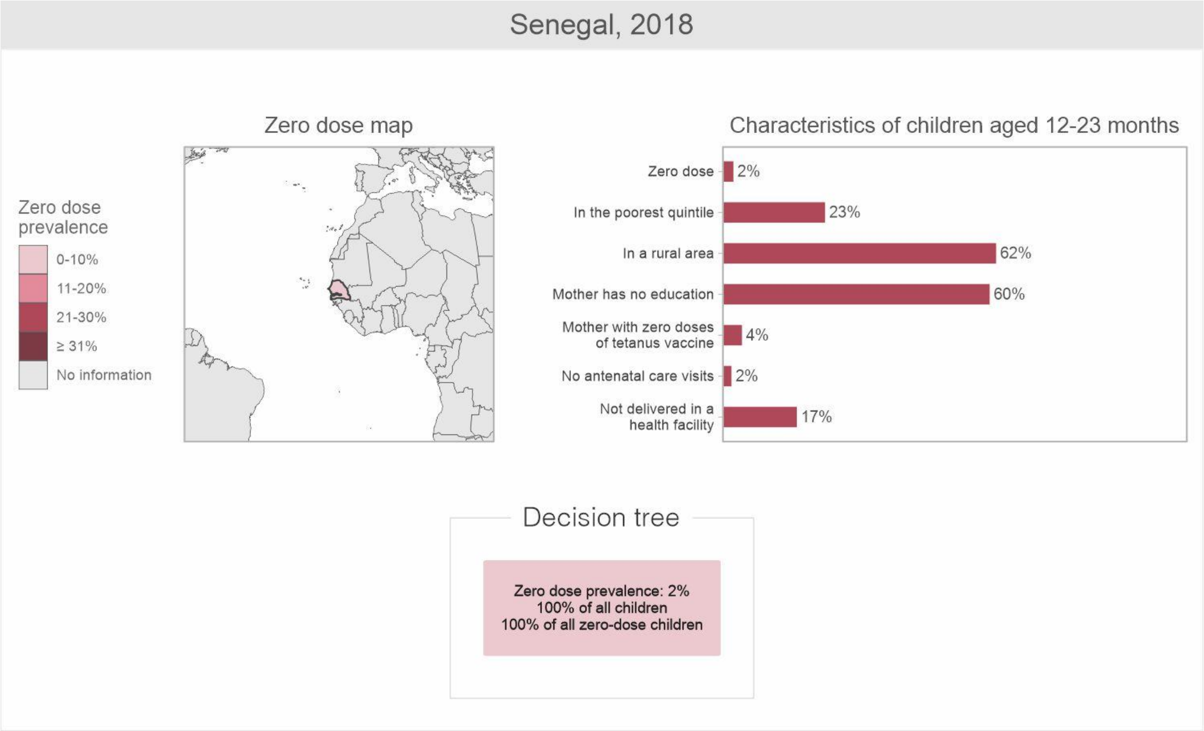

Figure S73. – Senegal's country profile.

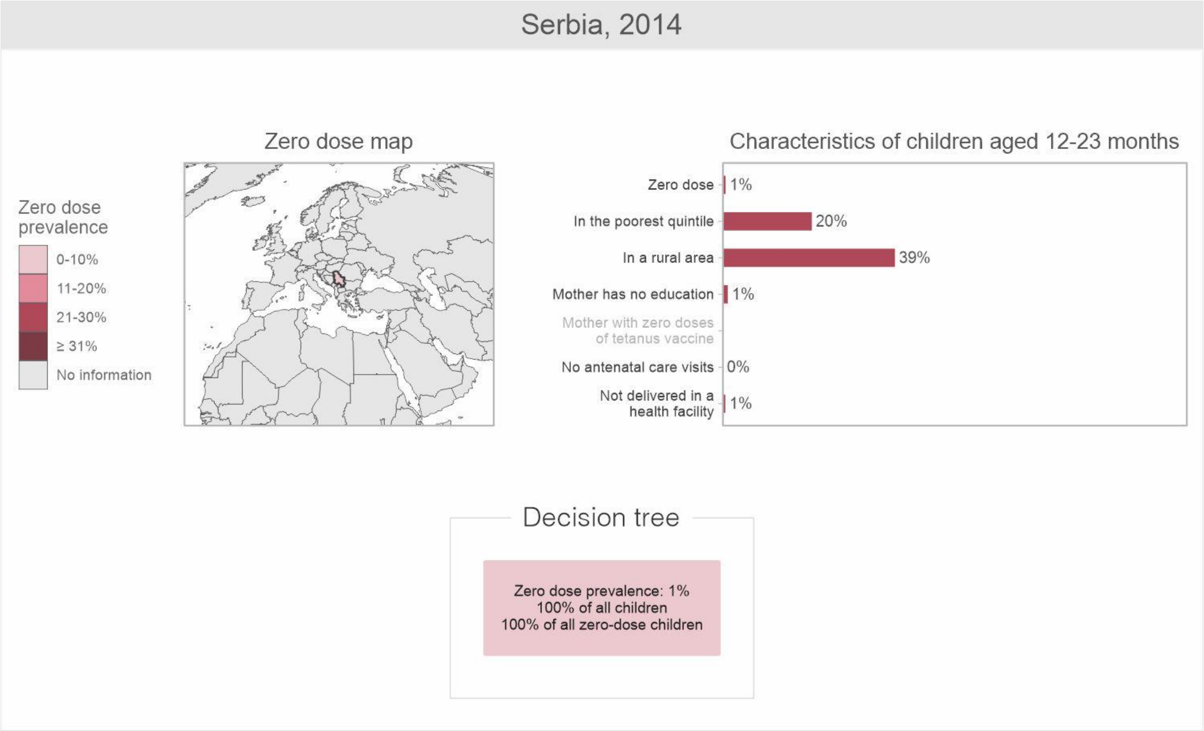

Figure S74. – Serbia's country profile.

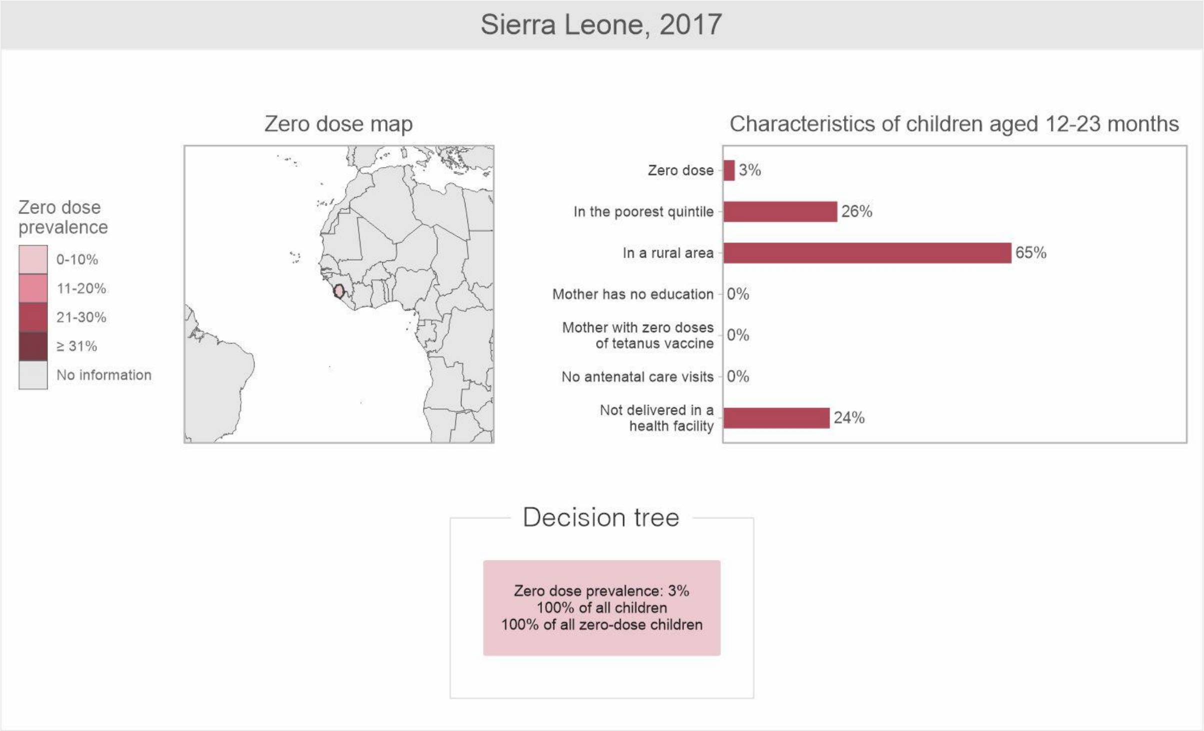

Figure S75. – Sierra Leone's country profile.

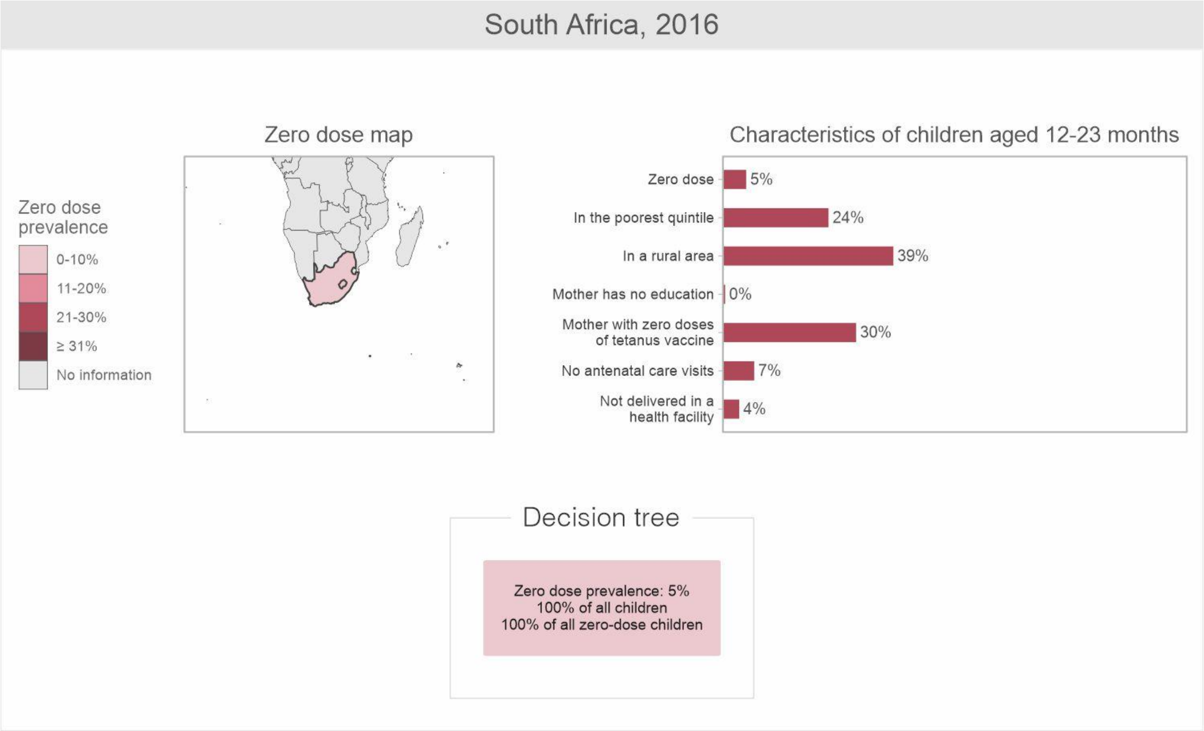

Figure S76. – South Africa's country profile.

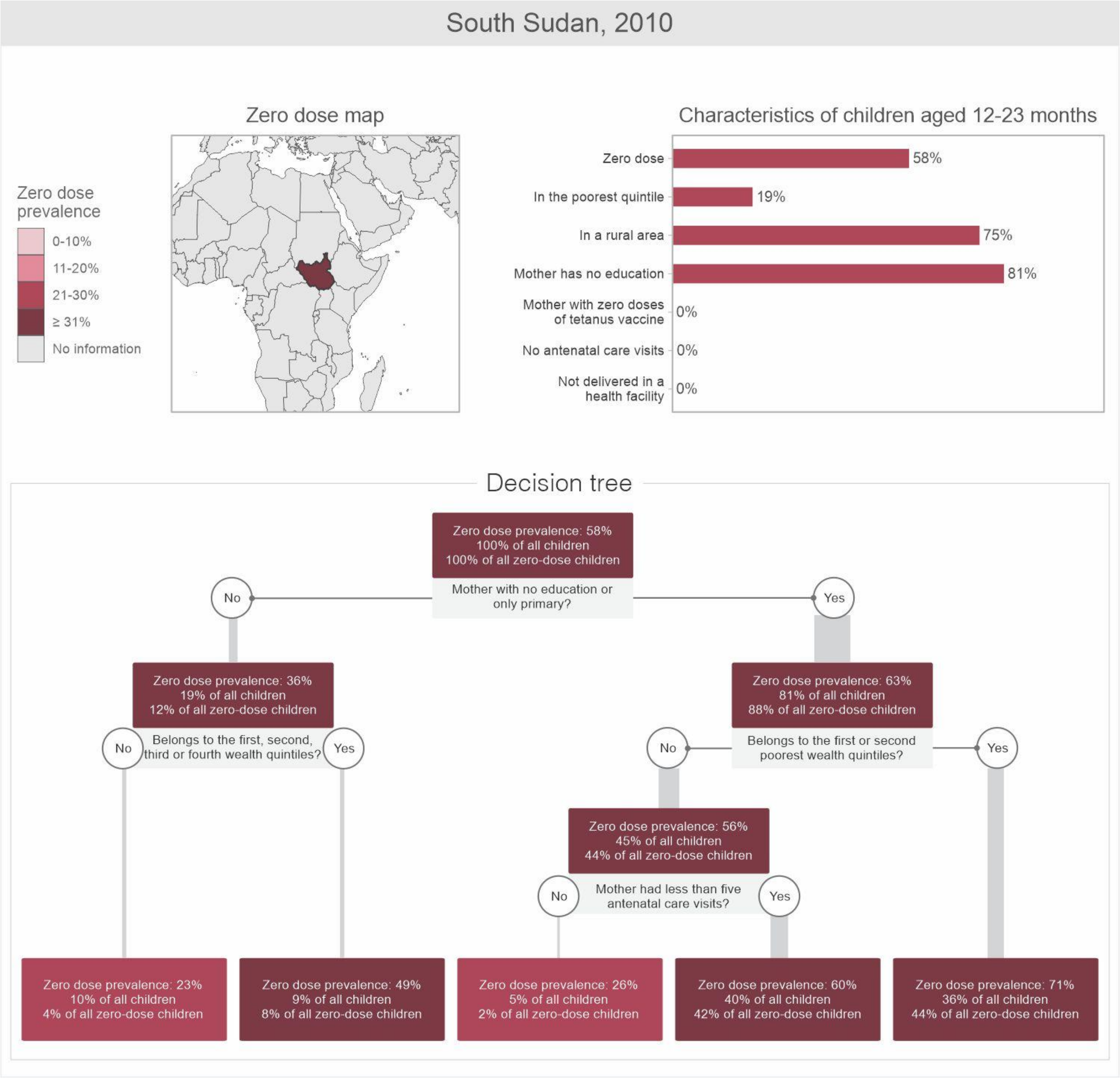

Figure S77. – South Sudan's country profile.

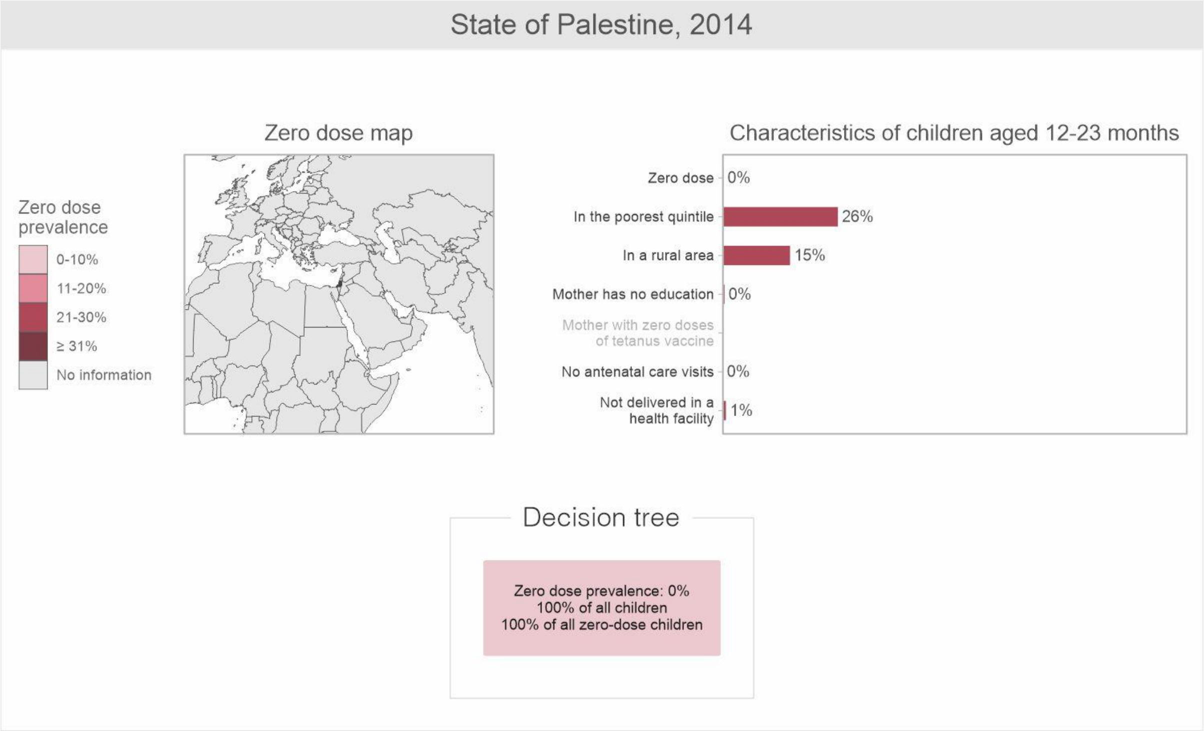

Figure S78. – State of Palestine's country profile.

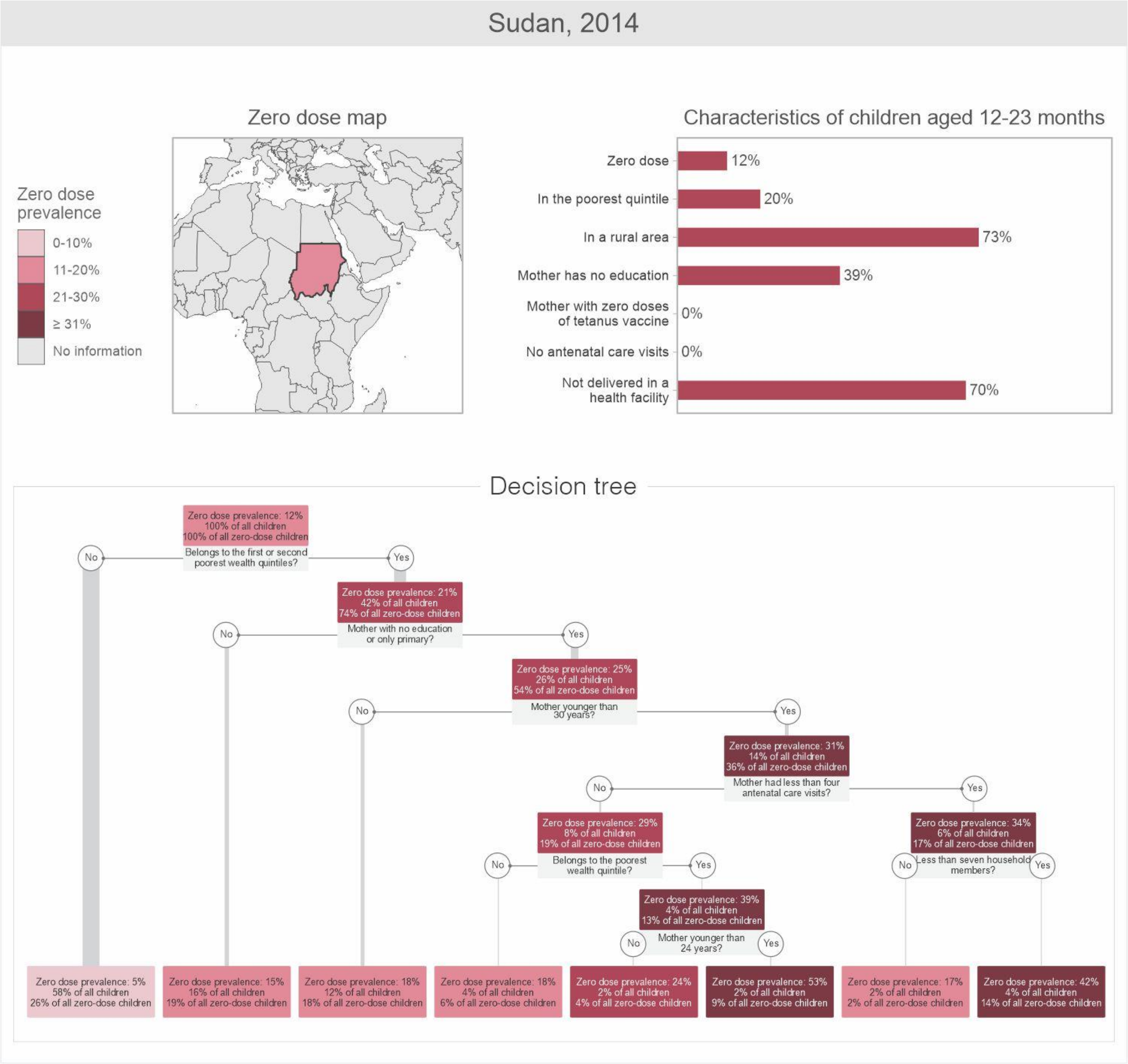

Figure S79. – Sudan's country profile.

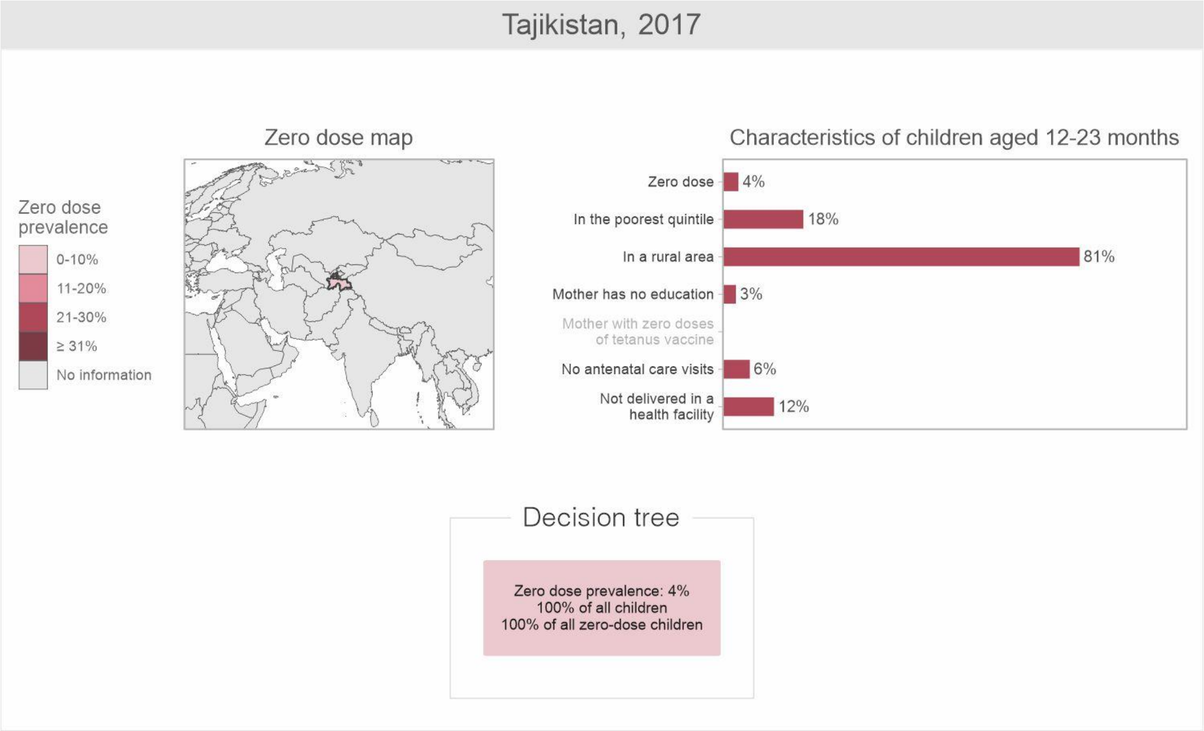

Figure S80. – Tajikistan's country profile.

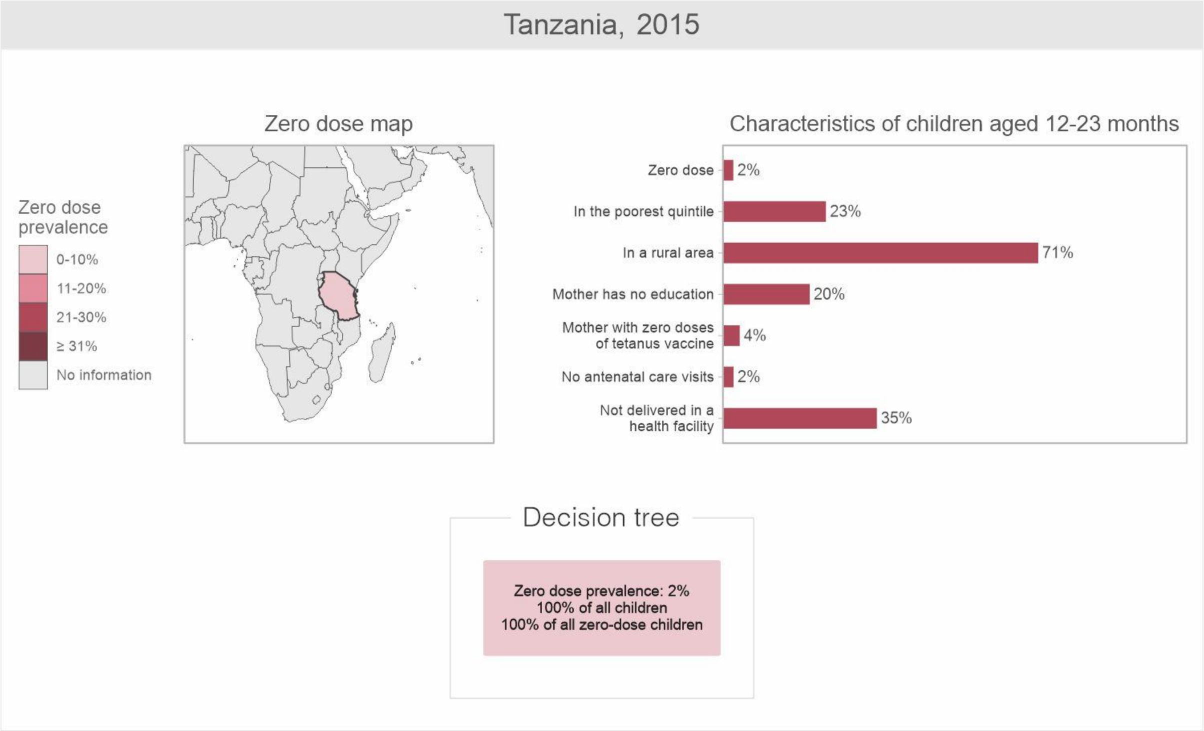

Figure S81. – Tanzania's country profile.

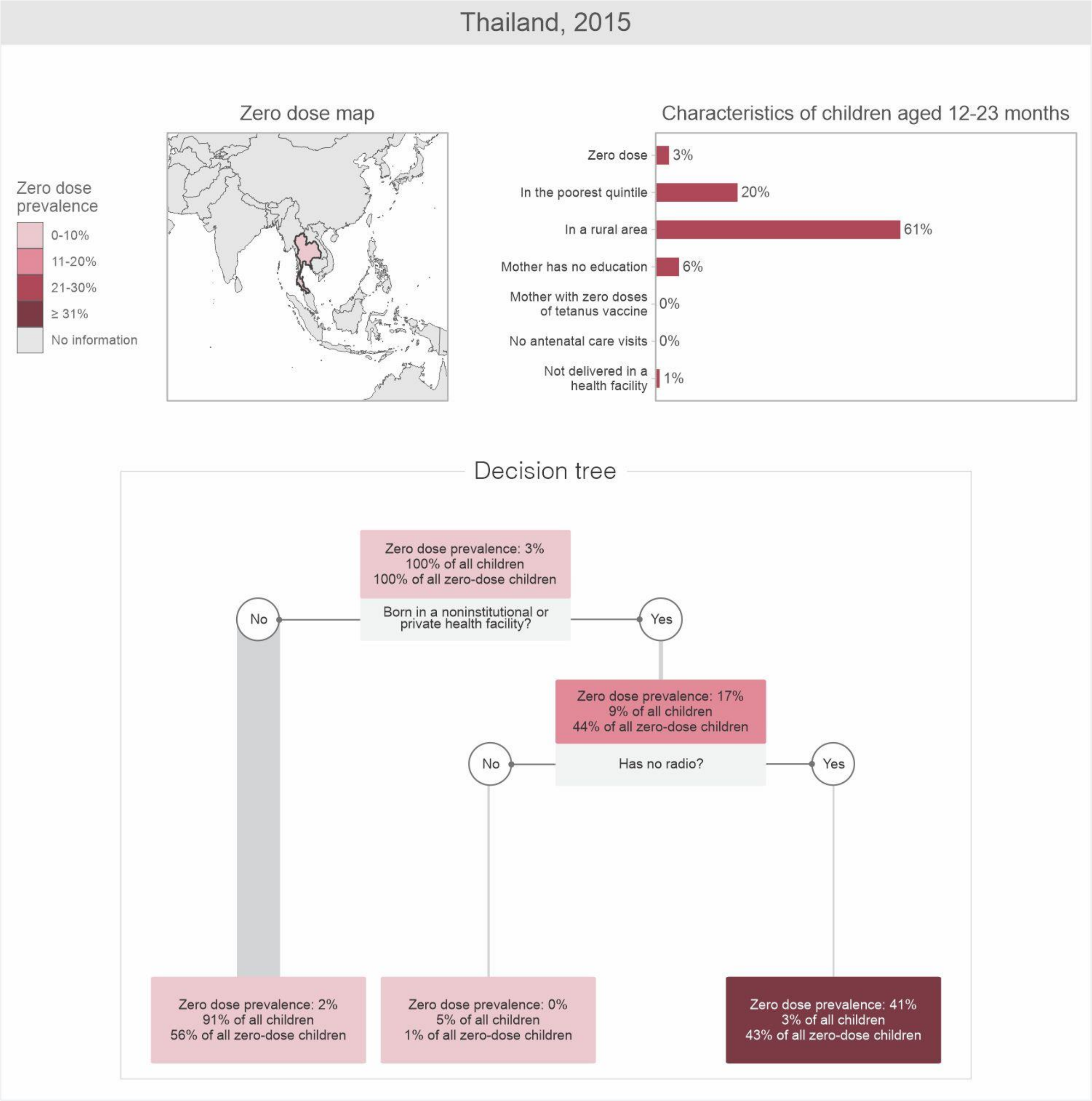

Figure S82. – Thailand’s country profile.

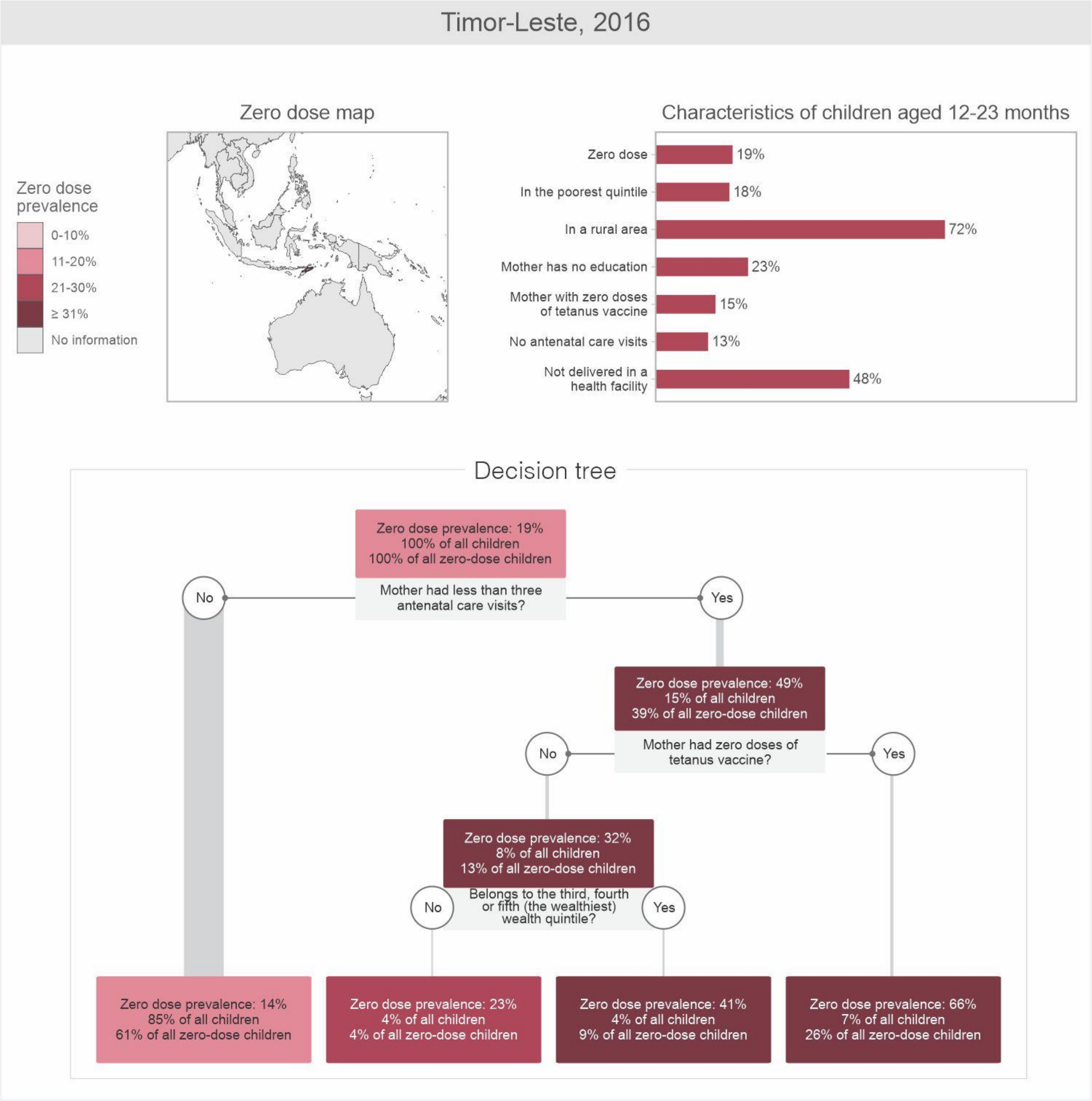

Figure S83. – Timor-Leste's country profile.

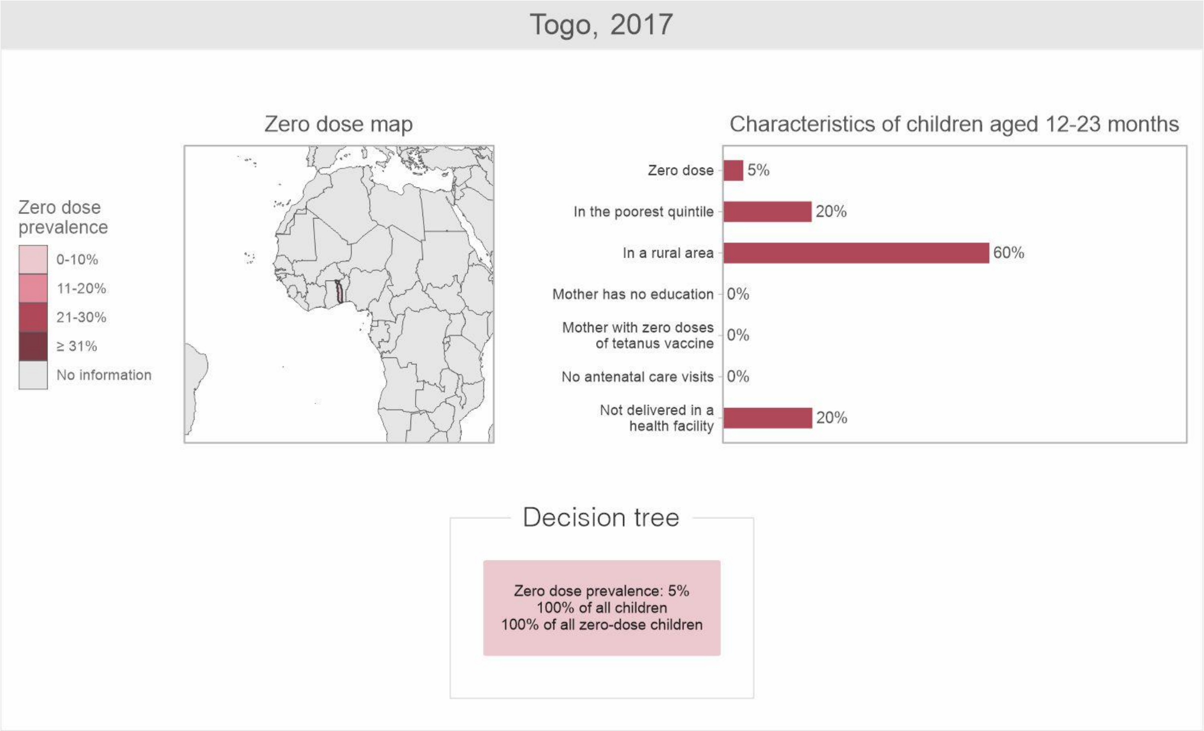

Figure S84. – Togo's country profile.

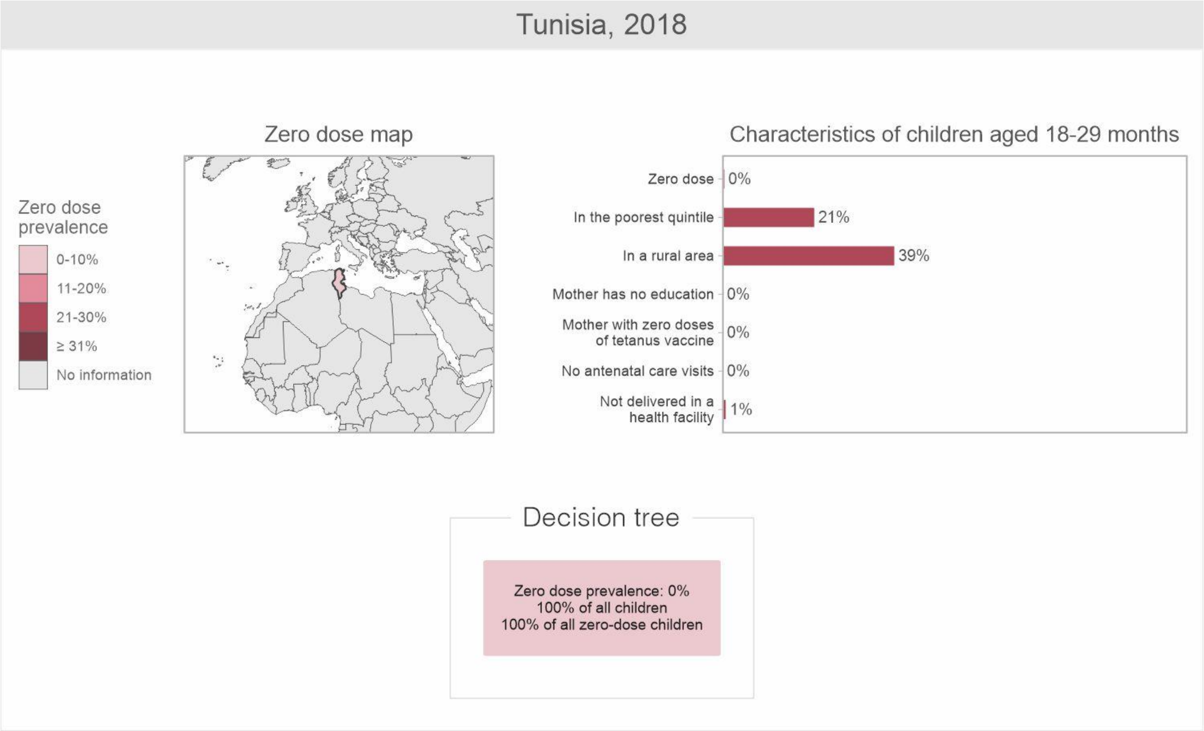

Figure S85. – Tunisia's country profile.

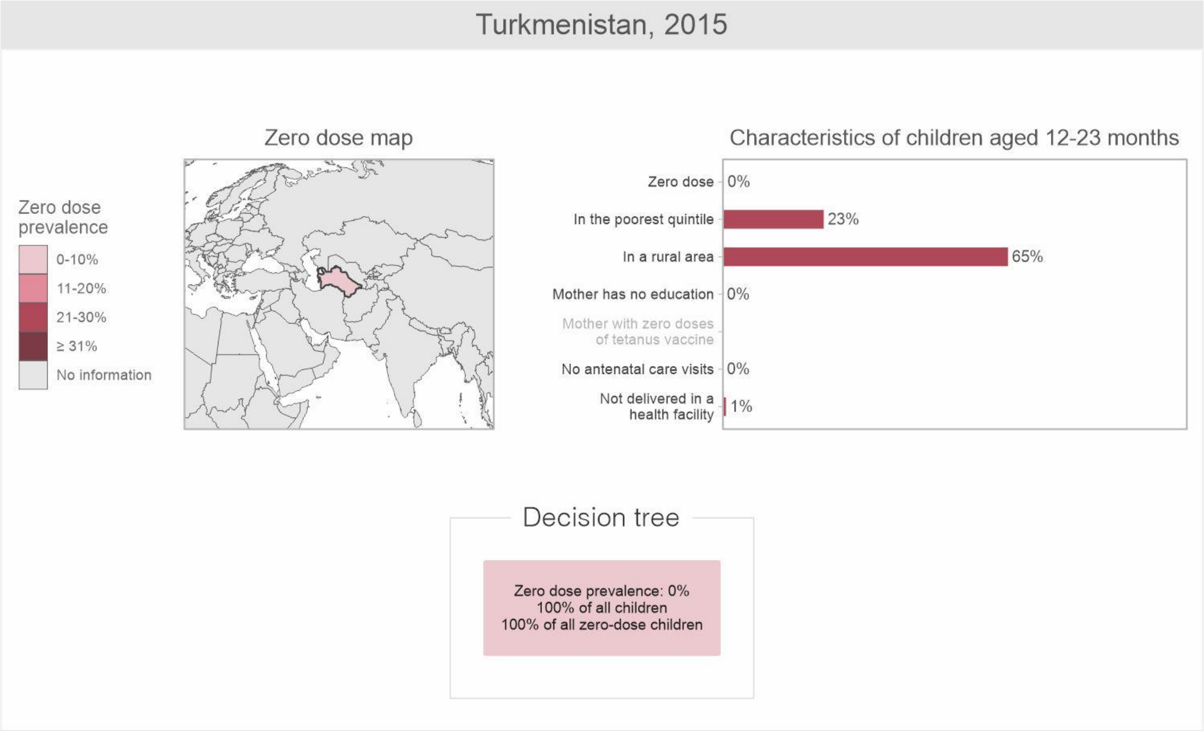

Figure S86. – Turkmenistan's country profile.

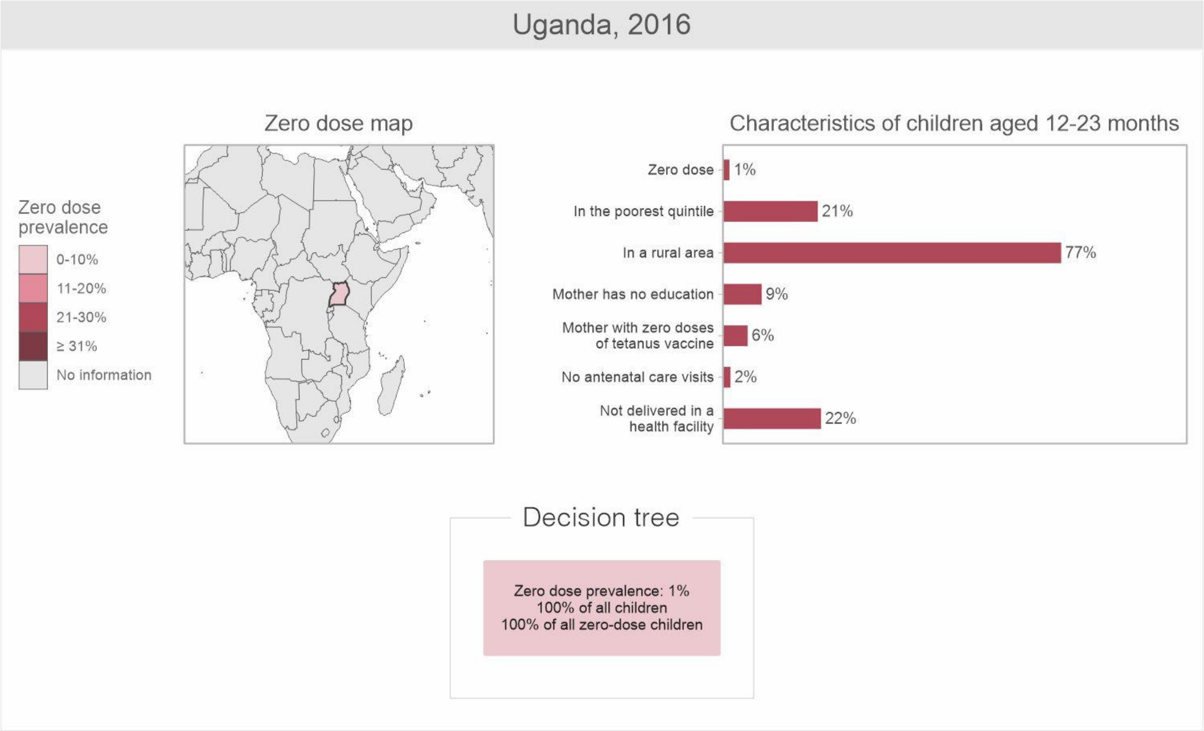

Figure S87. – Uganda's country profile.

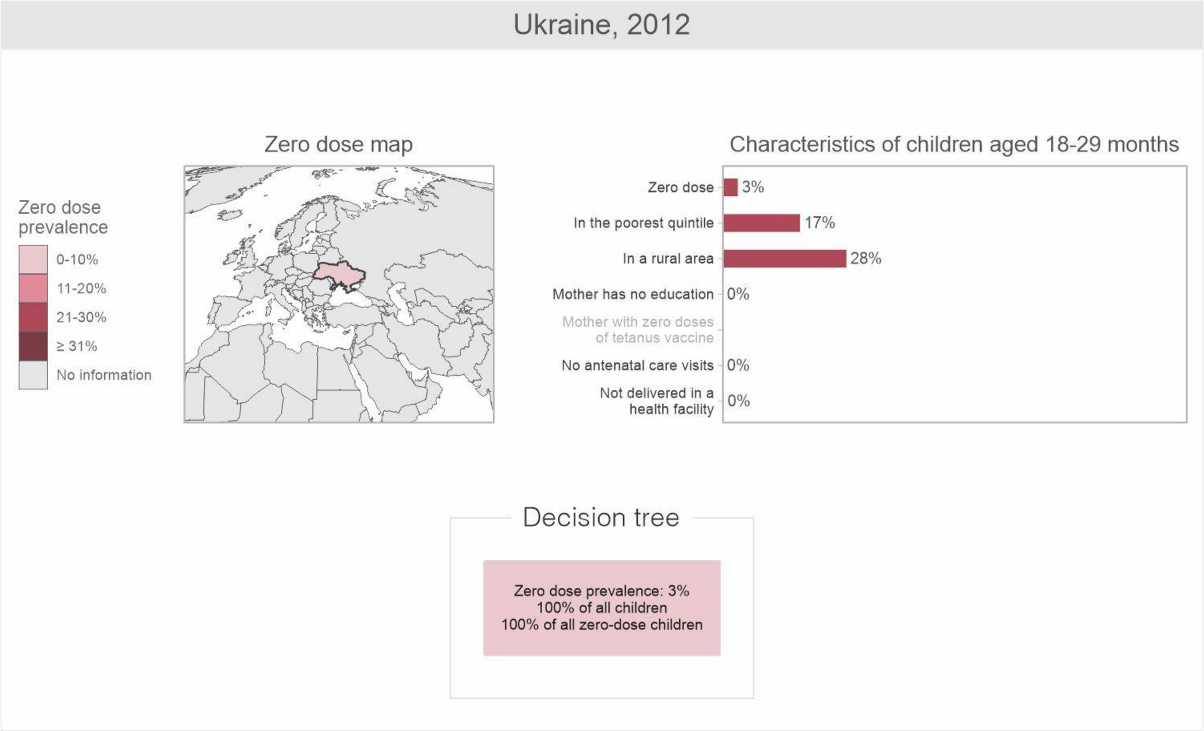

Figure S88. – Ukraine's country profile.

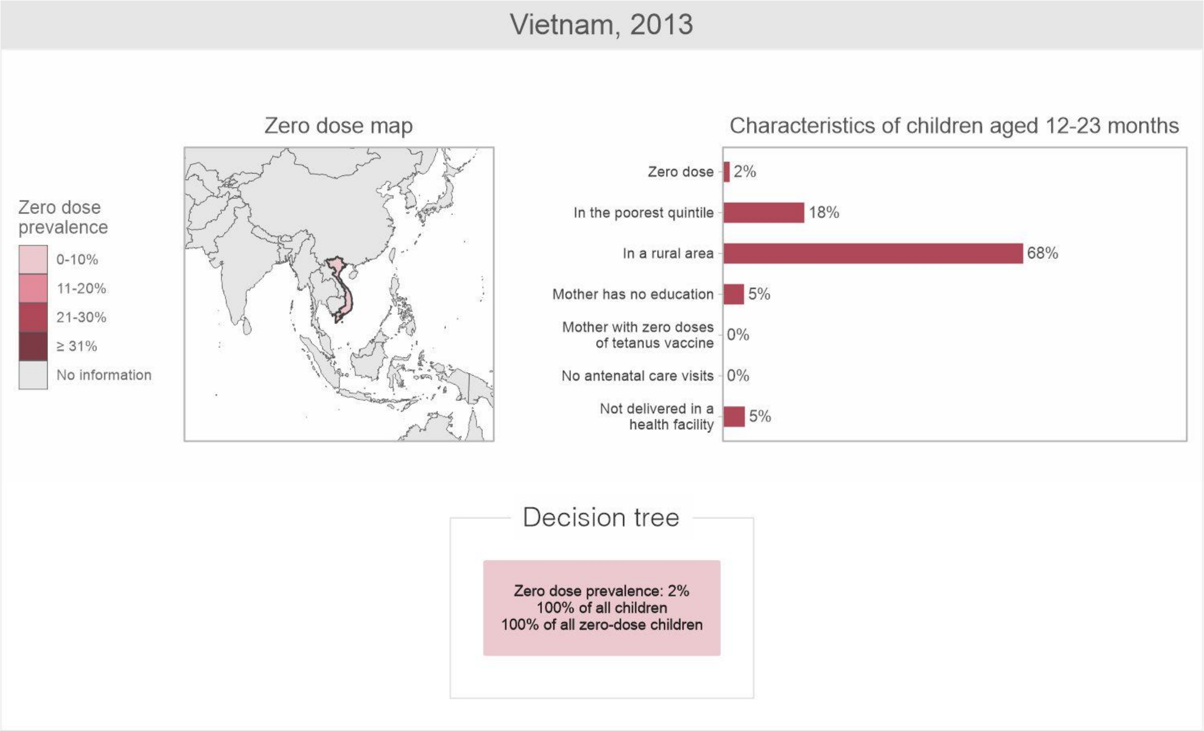

Figure S89. – Vietnam's country profile.

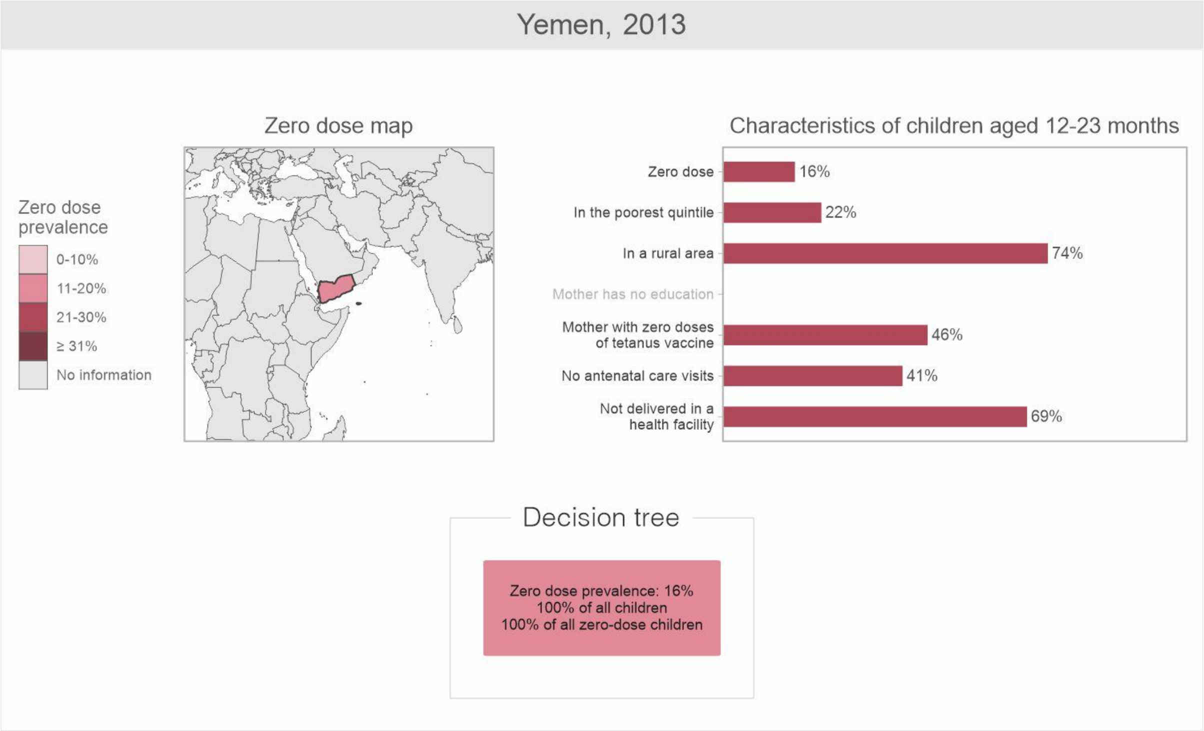

Figure S90. – Yemen's country profile.

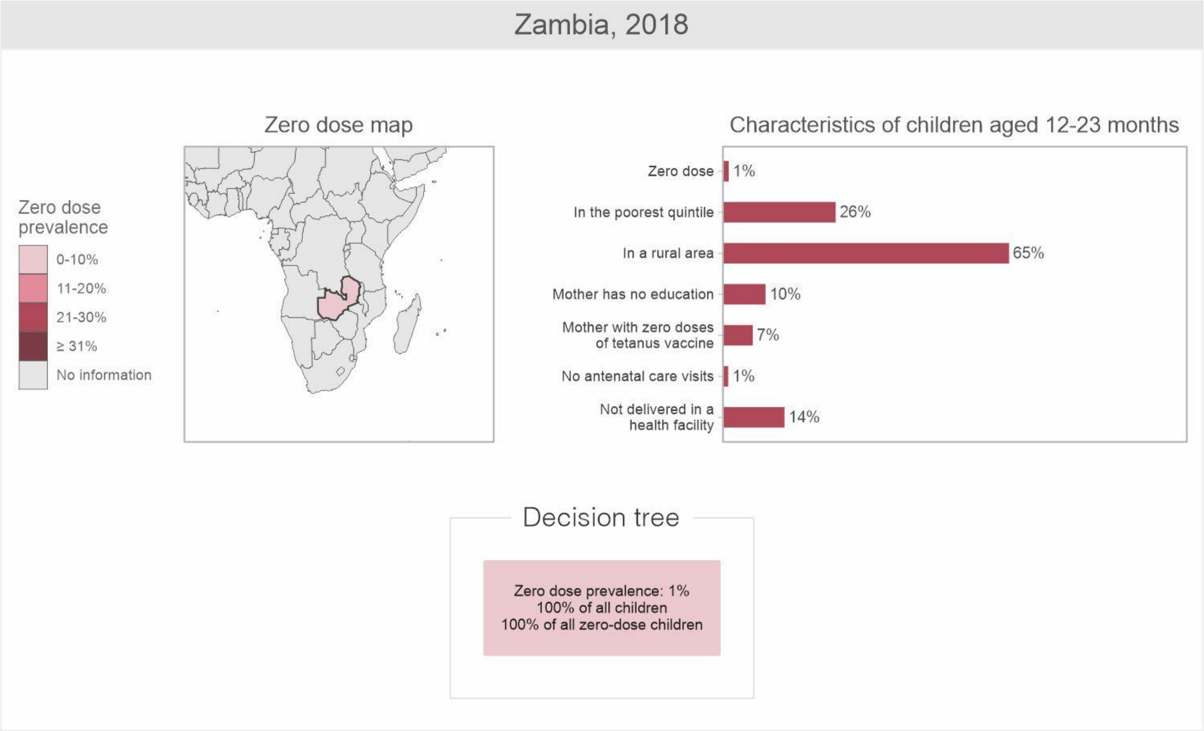

Figure S91. – Zambia's country profile.

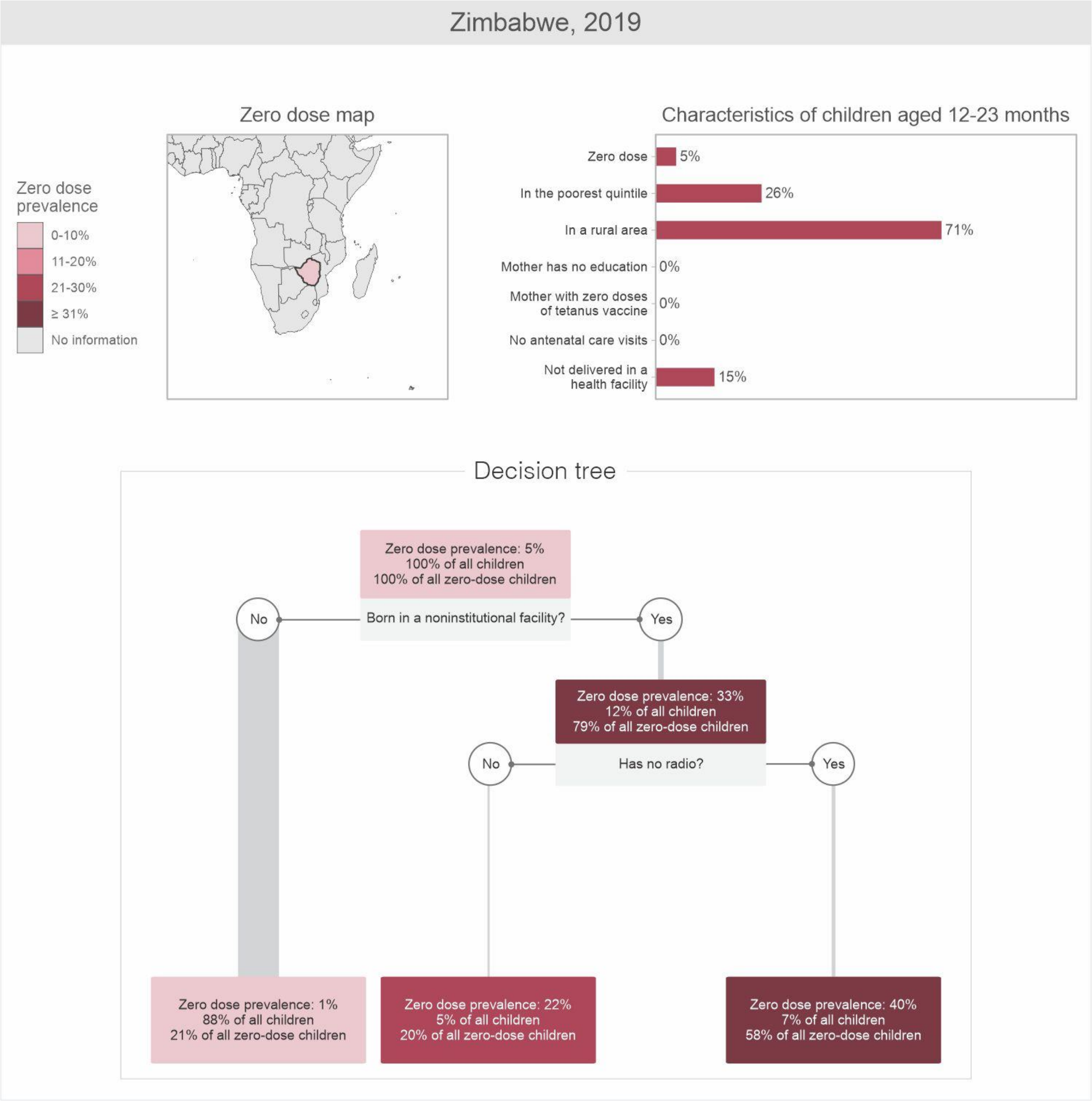

Figure S92. – Zimbabwe's country profile.
